# Systems biology prioritizes drug candidates for an interferon-response-high monocyte state in idiopathic normal pressure hydrocephalus

**DOI:** 10.3389/fnagi.2026.1971059

**Published:** 2026-10-23

**Authors:** Md. Masudul Haque, Jongwan Bae, Irin Sultana Nila, Kyunghun Kang, Janghyeok Yoon, Sohee Kim, Ki-Su Park

**Affiliations:** 1 DGIST InnoCORE Initiative, Multiscale Brain Digital Twin Convergence (BDT) Research Group, Daegu Gyeongbuk Institute of Science and Technology (DGIST), Daegu, Republic of Korea; 2 Department of Neurosurgery, Kyungpook National University School of Medicine, Daegu, Republic of Korea; 3 Finance School of Business Administration, Kyungpook National University, Daegu, Republic of Korea; 4 Department of Neurology, Kyungpook National University School of Medicine, Daegu, Republic of Korea; 5 Department of Industrial Engineering, Konkuk University, Seoul, Republic of Korea; 6 Department of Robotics and Mechatronics Engineering, Daegu Gyeongbuk Institute of Science and Technology (DGIST), Daegu, Republic of Korea

**Keywords:** drug repurposing, idiopathic normal pressure hydrocephalus, LINCS L1000, monocytes, neuroinflammation, single-cell RNA sequencing, transcriptomics, type I interferon

## Abstract

**Background:**

Idiopathic normal pressure hydrocephalus (iNPH) is an age-associated neurological disorder characterized by gait disturbance, cognitive impairment, and urinary dysfunction. Although cerebrospinal fluid dynamics are central to its pathophysiology, the contribution of peripheral immune alterations remains poorly understood. We characterized an interferon-response-high monocyte state observed in iNPH and evaluated computational compound prioritization.

**Methods:**

We used a fixed set of marker criteria to characterize the IFN-response-high monocyte state. We then compared monocyte clusters 5 and 0 and used 150 upregulated and 150 downregulated genes to screen compounds in LINCS. We tested whether candidate rankings changed with different state definitions, comparison groups, gene-list sizes, clustering settings, and inclusion of all patients. We also repeated the queries after excluding each donor while retaining the original cell-state labels and compared the results with 100 expression-matched random gene signatures. External blood datasets and paired blood–ventricular CSF samples provided additional context.

**Results:**

The marker rule classified 3,195 of 22,197 canonical monocytes as IFN-response-high. The cluster contrast identified 1,231 FDR-significant genes. Of 1,376 THP1 compounds, 138 met the operational criteria of normalized connectivity score (NCS) <−0.5 and WTCS FDR <0.05. Rosuvastatin and roflumilast ranked first and 14th in the primary screen and first and 15th in the integrated robustness ranking. Rosuvastatin met the operational criteria in all 11 core analyses; roflumilast did so in 10, returning NCS = 0 at resolution 0.7. Both met the criteria across all nine donor omissions. In exploratory comparisons performed after candidate selection, both observed scores were more negative than those from all 100 sampled expression-matched signatures. The blood-derived program was detectable in ventricular-CSF candidate monocytes, although unequal cell recovery limited inference about compartmental enrichment.

**Conclusion:**

This study characterizes an interferon-response-high classical-monocyte state observed within an iNPH cohort and nominates compounds with predicted inverse connectivity for experimental testing. Computational support was more consistent for rosuvastatin than for roflumilast. These findings support further investigation of peripheral innate immune states observed in an iNPH cohort and provide experimentally testable hypotheses for therapeutic repurposing.

## 1 Introduction

Idiopathic normal pressure hydrocephalus (iNPH) is a progressive, age-associated neurological disorder characterized by the clinical triad of gait disturbance, cognitive decline and urinary incontinence occurring in the context of ventricular enlargement without elevated intracranial pressure (Adams et al., 1965). The condition affects an estimated 5.9% of those over 80 years (Jaraj et al., 2014), making it one of the most prevalent potentially reversible causes of dementia in the elderly. Ventriculoperitoneal shunting, the current standard of care, produces meaningful clinical improvement in gait in the majority of treated patients; however, cognitive recovery remains incomplete in a substantial proportion, indicating an unmet need that may reflect irreversible injury, comorbid pathology, treatment timing, or biological processes not fully corrected by cerebrospinal fluid (CSF) diversion (Bonney et al., 2022; Costa et al., 2025). The pathophysiology of iNPH extends beyond mechanical CSF obstruction to encompass glymphatic dysfunction, blood-brain barrier disruption, cerebral hypoperfusion, and increasingly recognized neuroinflammatory mechanisms (Bonney et al., 2022; Costa et al., 2025; Perković and Borovečki, 2024). Understanding and therapeutically testing these inflammatory components is particularly relevant for patients with persisting or progressive cognitive impairment after surgical CSF diversion.

Recent advances in single-cell transcriptomics have revealed that peripheral immune cell states reflect and potentially contribute to central nervous system (CNS) pathology across a range of neurodegenerative conditions (Jacobs et al., 2025; Sirkis et al., 2023). Circulating monocytes can respond to and reflect systemic inflammatory signals, and have been proposed as accessible readouts of neuroinflammatory activity in CNS disease. In iNPH specifically, Duy and colleagues recently generated a single-cell RNA sequencing atlas of peripheral blood mononuclear cells and ventricular CSF from iNPH patients, identifying proinflammatory alterations in monocytes that were significantly enriched in patients with lower baseline cognitive function as measured by the Montreal Cognitive Assessment (MoCA) (Duy et al., 2025). This finding motivates further examination of peripheral immune states in iNPH. However, the source-study association does not establish that the particular monocyte state examined here contributes to cognitive impairment or that modifying this state would improve clinical outcomes.

Closer examination of the iNPH monocyte transcriptome reveals a distinct type I interferon-response-high subcluster within the classical monocyte population of patients with iNPH. Type I IFNs (IFN-α and IFN-β) signal through a common heterodimeric receptor (IFNAR), activating the Janus kinase and signal transducer and activator of transcription (JAK/STAT) pathway and driving transcription of a stereotyped panel of interferon-stimulated genes, including IFIT1, IFIT2, ISG15, MX1, OAS1, CXCL10, and SIGLEC1 (Roy et al., 2020; Viengkhou and Hofer, 2023). Although type I IFN responses were originally characterized as antiviral defense mechanisms, chronic dysregulation of interferon signaling in the ageing CNS is increasingly recognized in neuroinflammation and cognitive decline (Tan et al., 2022). IFN signaling is elevated in the choroid plexus in aged humans and mice, and pharmacological blockade of IFNAR reverses the aged transcriptomic phenotype in animal models (Baruch et al., 2014). Type I IFN has also been shown to drive neuroinflammation and synapse loss in Alzheimer disease (Roy et al., 2020); however, the present dataset does not establish direct pathway activation or causality in iNPH.

More than six decades after iNPH was first described, no pharmacologic treatment has been approved; a small, uncontrolled acetazolamide study provided only Class IV evidence and did not establish an alternative to shunting (Adams et al., 1965; Alperin et al., 2014). This persistent therapeutic gap supports investigation of interferon-associated signaling as a pharmacologically tractable, experimentally testable hypothesis.

Computational drug repurposing offers a systematic and resource-efficient strategy to identify FDA-approved compounds predicted to reverse disease-associated transcriptional states without the time and cost of *de novo* drug development. The Connectivity Map framework, originally introduced by Lamb and colleagues (Lamb et al., 2006) and scaled by a factor of 1,000 through the NIH Library of Integrated Network-Based Cellular Signatures (LINCS) consortium (Subramanian et al., 2017), enables transcriptional signatures of disease to be queried against a database of pharmacologically induced gene expression profiles. Compounds whose transcriptional perturbation signature is inversely correlated with the cell-state signature are identified as candidate reversal compounds. This approach has been successfully applied to repurpose drugs across oncology, infectious disease, and neurological conditions, and offers particular advantages for rare or understudied diseases where clinical trial infrastructure is limited.

Here, we apply a computational drug-repurposing pipeline to an IFN-response-high monocyte state observed in patients with iNPH, using publicly available single-cell RNA sequencing data. We first characterize this state using marker-based scoring and clustering, and derive a within-iNPH cell-state signature through donor-paired comparison of an IFN-response-high subcluster with a low-IFN comparator. We then contextualize the signature against monocytes from patients with systemic lupus erythematosus and healthy controls, and query it against the LINCS2 perturbation database using weighted connectivity scoring. To assess the stability of compound prioritization, we examine alternative state definitions, comparator populations, signature sizes, and clustering resolutions, together with donor-inclusion and leave-one-donor-out analyses. We further compare the highlighted compounds’ connectivity scores with expression-matched random signatures and examine whether the blood-derived program is detectable in paired ventricular-CSF monocytes. Rosuvastatin emerged as the strongest primary THP1 candidate and ranked first in the integrated robustness analysis, while roflumilast was retained as a *post hoc* candidate with a complementary pharmacological rationale but less consistent rankings. A separate mechanistic review identified baricitinib as a secondary candidate with significant inverse THP1 connectivity. Together, these analyses characterize a monocyte state observed in iNPH and assess the strength and limitations of compound-prioritization hypotheses for subsequent experimental testing.

## 2 Materials and methods

### 2.1 Study design

This secondary computational study used publicly available single-cell RNA sequencing data. The workflow comprised a canonical nine-donor PBMC monocyte population, a cluster-5-versus-cluster-0 paired pseudobulk contrast, and a primary 150-up/150-down gene connectivity query. Further analyses added a frozen marker-defined state, alternative state/comparator and signature-size queries, clustering-resolution sensitivity, a uniform ten-donor reconstruction, candidate-level leave-one-donor-out connectivity, expression-matched null comparisons, and ventricular-CSF contextualization. Donor-level ventricular-CSF and paired PBMC–CSF outputs are provided in Supplementary Data 1; query-level candidate-robustness outputs are provided in Supplementary Data 2. No new participants were recruited. All analyzed data were deidentified and publicly available; the ethics and consent procedures reported by the source studies are summarized in the Ethics statement.

### 2.2 Single-cell RNA sequencing dataset

Raw data were available for ten patients, whereas the released canonical monocyte object contained nine donors and did not include Patient 6. Patient 6 also lacked publicly linked MoCA information, but missing cognitive information was not treated as a biological exclusion criterion. We evaluated this donor in a separate, uniform ten-donor reconstruction while preserving the provenance of the canonical nine-donor analysis. For the nine-donor PBMC reconstruction, 124,047 cells were available before quality control; cells with fewer than 200 detected genes or more than 20% mitochondrial transcripts were excluded, leaving 122,724 cells. No three-median-absolute-deviation total-count filter was applied. Publicly linkable donor characteristics and analysis-specific cell counts are reported in Supplementary Table 1.

### 2.3 PBMC annotation and classical monocyte identification

The 22,197 classical monocytes were defined by the released canonical monocyte object, and their exact cell identifiers were mapped to the reconstructed PBMC embedding. The remaining 100,527 retained PBMCs were labeled as other cells for Supplementary Figure 1. Classical-monocyte identity was evaluated using CD14, LYZ, S100A8, S100A9, VCAN, and FCN1 together with T-cell, B-cell, natural-killer-cell, and dendritic-cell markers.

### 2.4 Identification of the ISG-high monocyte cluster

Classical monocytes were analyzed in Seurat (Hao et al., 2021). NormalizeData used default settings; FindVariableFeatures selected 2,000 genes; ScaleData and principal-component analysis used 30 components. Harmony integrated by patient (Korsunsky et al., 2019). FindNeighbors used Harmony dimensions 1–30; FindClusters used resolution 0.5, Louvain algorithm 1, and random seed 0. UMAP used the uwot cosine implementation with random seed 42. Fixed-seed deterministic reruns were compared for exact equality.

### 2.5 Marker-defined state and population sensitivity

The primary descriptive state definition was a frozen 15-gene IFN-selector rule, independent of cluster membership. The selector comprised ISG15, IFI6, IFI44, IFI44L, MX1, MX2, OAS1, OAS2, OAS3, IFIT1, IFIT3, RSAD2, STAT1, USP18, and LY6E. Cells were classified as IFN-response-high when their saved AUCell selector score was at least the donor-balanced external healthy-control threshold (approximately 0.0973), using the full-precision stored threshold. This is an operational definition, not a validated diagnostic cutoff. We assessed its overlap with cluster 5 and applied the same rule to the uniform ten-donor reconstruction. The marker-defined population characterized state frequency and supplied an alternative differential-expression query; it did not replace the cluster-derived PRIMARY_150 query.

To assess clustering dependence, we repeated Louvain clustering at resolutions 0.3, 0.5, 0.7, and 1.0 within the retained analysis framework, selected the IFN-response-high cluster using the existing IFN-marker scoring rule, and compared cell counts and Jaccard overlap with cluster 5. Resolution-specific drug queries compared the selected cluster with all remaining candidate monocytes. Patient 6 sensitivity used a uniform reconstruction across all ten donors, followed by the frozen marker rule and a marker-high-versus-marker-low pseudobulk contrast. These analyses test selected analytical choices; they do not constitute an exhaustive sensitivity analysis of integration methods or a full donor-omission reconstruction of the discovery pipeline.

### 2.6 ISG scoring and cognitive-group comparison

For each cell, the diagnostic ISG score was the mean log-normalized expression of IFITM3, IFITM2, ISG15, MX1, OAS1, IRF7, and STAT1; cluster values were cell-level means. This seven-gene cluster-description panel is distinct from the frozen 15-gene selector used for the marker-defined state. Cluster 5 frequency was calculated per patient as the percentage of canonical monocytes assigned to cluster 5. High-MoCA (*n* = 5; scores 23–29) and Low-MoCA (*n* = 4; scores 19–20) groups were compared using a two-sided Wilcoxon rank-sum test. Exact patient-linked MoCA scores were unavailable.

### 2.7 Paired pseudobulk differential expression and signature construction

The primary differential-expression contrast compared cluster 5 with cluster 0 within the same nine donors. Cluster 0 was retained as the original low-IFN comparator and supplied sufficient cells for paired pseudobulk analysis in every donor; its use does not imply that it is the only biologically valid comparator or that it represents healthy or functionally resting monocytes. Counts were summed by patient and state using scuttle::aggregateAcrossCells. Patient-state samples required at least 30 cells, and all 18 samples were retained. DESeq2 used design ∼ patient + state (Love et al., 2014), and apeglm was used for log_2_ fold-change shrinkage (Zhu et al., 2019). Genes with FDR <0.05 were ranked separately by shrunken fold change; the first 150 positive and 150 negative genes formed PRIMARY_150. Recovery of 32 members of an internal 35-gene ISG panel was treated as a workflow-coherence check rather than independent validation.

To evaluate comparator dependence, we additionally compared cluster 5 with all remaining monocytes, cluster 5 with non-cluster-5 marker-low monocytes, and marker-defined IFN-high with marker-low monocytes. Alternative-resolution and ten-donor marker contrasts were analyzed using the same paired pseudobulk framework and minimum-cell rule. The original contrast was queried at 50, 100, 150, and 200 genes per direction; alternative contrasts used balanced positive and negative sets of up to 150 significant genes each, reducing both directions to the smaller available number when necessary. Query-specific NCS, WTCS FDR, average-tie ranks, and represented query sizes for the integrated top-20 compounds are provided in Supplementary Data 2, together with all-compound summary statistics and donor-omission results for the two highlighted candidates. These alternatives explicitly test the consequences of the original comparator choice rather than assuming equivalent signatures.

### 2.8 Cross-dataset contextualization

Classical monocytes from the Perez SLE atlas (Perez et al., 2022) comprised 307,429 cells. The 15 selector genes were ISG15, IFI6, IFI44, IFI44L, MX1, MX2, OAS1, OAS2, OAS3, IFIT1, IFIT3, RSAD2, STAT1, USP18, and LY6E. To avoid direct reuse, these genes were removed from the positive query, leaving 135 positive and 150 negative genes. AUCell was run on raw counts in blocks of 10,000 cells with aucMaxRank = 1,500 (Aibar et al., 2017); the score was AUC_positive_ − AUC_negative_. A sensitivity score used mean positive minus mean negative expression on log-normalized counts.

Within SLE and healthy-control groups separately, interferon-high cells were selected as the top quartile of the 15-gene score; complete iNPH cluster 5 was retained without further selection. Donor means were compared with two-sided Wilcoxon rank-sum tests after excluding donors with fewer than 10 qualifying cells. The AUCell analysis retained 143 SLE donors, whereas the mean-difference analysis retained 139 because qualification was score-specific; both included nine iNPH and 99 healthy-control donors. Reported *P*-values are nominal and unadjusted.

For burden, each healthy-control donor contributed its own 75th-percentile 15-gene score, and the median donor threshold was applied across groups (AUCell threshold 0.0973; mean-difference threshold 0.2517). Burden analyses included 162 SLE, 99 healthy-control, and nine iNPH donors. The same external healthy controls calibrated and evaluated the threshold. Diagnosis is fully confounded with dataset and processing, so external healthy-control contrasts were not interpreted as disease effects.

The complete iNPH cluster 5 and externally selected IFN-high cells are not identically ascertained populations. Removing the selector genes from signature scoring reduces direct gene reuse but does not eliminate selection effects, correlated gene programs, or dataset confounding. External comparisons were therefore interpreted as descriptive contextualization, not as independent validation or estimates of an iNPH disease effect. The shared threshold was also applied to the full canonical iNPH monocyte population for the marker-defined state description. Pooled cell percentages and medians of donor-level percentages were reported separately.

### 2.9 LINCS2 transcriptional connectivity scoring

The LINCS2 reference was accessed through ExperimentHub resource EH7297 and derives from the LINCS2020 beta release. It contains 136,460 Level-5 exemplar signatures representing 30,456 compounds across 58 cell lines. Level-5 signatures combine replicate profiles within an experimental condition. Separately, the reference selects a CLUE-designated exemplar for each represented compound-cell-line combination, generally based on transcriptional activity score. We used the resource’s designated exemplars rather than aggregating profiles across different doses or exposure durations.

Gene symbols were mapped to Entrez identifiers using org.Hs.eg.db. Of 150 positive genes, 143 mapped to Entrez identifiers and 114 were represented in LINCS2; of 150 negative genes, 137 mapped and 101 were represented. Overall, 215 of 300 genes (71.7%) entered the connectivity query. Weighted Connectivity Score (WTCS) and NCS were calculated with signatureSearch::gess_lincs (Duan et al., 2020). WTCS FDR values were Benjamini–Hochberg adjusted across all 136,460 returned perturbational entries.

THP1 was the primary monocytic screening context. It is a transformed leukemia-derived cell line, not a surrogate for primary monocytes from patients with iNPH. Negative NCS indicates inverse transcriptional connectivity; positive NCS indicates positive connectivity. After removal of non-BRD entries, the primary THP1 analysis contained 1,376 BRD-coded compounds. We used NCS <−0.5 together with WTCS FDR <0.05 as an operational screening rule. The NCS cutoff is heuristic and is not a validated efficacy threshold.

Exact-zero NCS values were retained in numerical accounting and ranking. Inspection of the installed scoring implementation showed that the two-sided WTCS calculation assigns zero when the up- and down-signature enrichment scores have the same sign. This identifies an algorithmic route to zero, but the saved compound-level results do not establish the individual cause of every zero entry. NCS = 0 was not treated as missing data or evidence of biological neutrality. The distribution panel displays nonzero values and explicitly reports the omitted zero count. Ranks were calculated among all 1,376 compounds, ordered by increasing NCS, with average ranks assigned to ties.

The top-30 primary-screen review and choice of highlighted compounds were *post hoc*. Regulatory status and biological rationale informed which compounds were discussed but were not a prospectively specified quantitative score. To make the computational comparison reproducible, a separate integrated robustness ranking was calculated. Compound identity was tracked by BRD identifier, with curated names and stereochemical qualifications retained where required. Original reference signatures were recovered by matching the compound identifier, cell line, and perturbation type to records designated as exemplars in the LINCS2020 siginfo_beta.txt metadata. All 136,460 saved reference identifiers matched this mapping, including the 1,376 THP1 entries. Original signature identifiers, doses, exposure durations, replicate identifiers, and signature-quality metrics are reported in Supplementary Data 2. The project lockfile records R 4.5.3, signatureSearch 1.24.0, signatureSearchData 1.24.0, ExperimentHub 3.0.0, AnnotationHub 4.0.0, and rhdf5 2.54.1. These records document the declared lockfile and provenance-audit environments but do not independently establish which package versions were loaded during every original query execution.

### 2.10 Hallmark interferon-α pathway sensitivity analysis

The MSigDB Hallmark IFN-α-response set M5911 contained 97 genes (Liberzon et al., 2015). Forty-two genes overlapped with the positive iNPH signature and one overlapped with the negative signature, giving 43 unique exclusions and 54 non-overlapping genes. Forty-nine of those 54 genes were represented in LINCS2 and were queried as an up-only sensitivity signature. THP1 NCS and FDR were extracted for a curated report set containing the two highlighted compounds, baricitinib, approved JAK inhibitors, and conventional disease-modifying antirheumatic drugs. Because the query shares no genes with the primary iNPH signature but uses the same perturbational resource and scoring framework, it is described as a non-overlapping-gene sensitivity analysis, not independent validation. The Hallmark query tests related pathway-level connectivity in the same perturbational resource. Gene-set non-overlap does not establish biological independence or distinguish IFN-selective effects from broader anti-inflammatory transcriptional effects.

### 2.11 Robustness assessment

*Conditional donor-omission and candidate robustness*. For each of the nine canonical donors, we removed that donor from both cluster 5 and cluster 0 and refit the paired pseudobulk model. The top 150 significant positive and negative genes were selected using the primary ranking rules. Gene-set overlap was summarized by Jaccard similarity; 0.70 was an operational visual reference, not an externally validated stability threshold. Persistence was assessed for the original 150 positive genes. Each donor-omission signature was queried against the full LINCS reference, and THP1 NCS, saved WTCS FDR, and average-tie rank were extracted. The state labels were retained, so these analyses assess conditional signature and candidate stability rather than repeating the entire single-cell discovery pipeline.

The core robustness analysis comprised 11 queries: PRIMARY_150; the original contrast at 50, 100, and 200 genes per direction; cluster 5 versus all remaining monocytes; cluster 5 versus non-cluster-5 marker-low monocytes; marker-high versus marker-low monocytes; selected IFN-high clusters at resolutions 0.3, 0.7, and 1.0 versus all remaining monocytes; and the ten-donor marker contrast including Patient 6. For each query, rank percentile was calculated as (average-tie rank − 1)/(1,376 − 1), with lower values indicating stronger relative inverse connectivity. Compounds were ordered by their median percentile across the 11 core queries. Remaining ties were resolved by a higher fraction of negative core NCS values, a lower primary-query percentile, and BRD identifier. Donor-omission, Hallmark, and matched-null queries were excluded from this integrated ranking and reported separately. This ranking describes computational consistency, not clinical utility or independent validation.

*Expression-matched null signatures*. We generated 100 random signatures with 150 genes in each direction, excluding all genes in PRIMARY_150. Matching used the primary DESeq2 baseMean expression values on the log(1 + expression) scale and preserved the direction-specific numbers of LINCS-represented, Entrez-mapped-but-unrepresented, and unmapped genes. For each target slot, one gene was sampled from up to 25 eligible nearest-expression candidates within the same mapping class, without reuse within a query or overlap between its up and down sets. The random seed was 20260903. Each null signature was queried against the 1,376 THP1 compound exemplars from the same LINCS2 resource. These queries supplied compound-specific NCS distributions; subset-adjusted FDR values were not used for inference. The primary, biological-sensitivity, donor-omission, and Hallmark queries used the full reference. For each highlighted compound, the nominal lower-tail empirical probability was (1 + number of null NCS values ≤ observed PRIMARY_150 NCS)/101. Because the highlighted compounds were selected after inspection of the primary screen, these are exploratory, post-selection comparisons. Their nominal empirical probabilities are limited by the 100-null resolution and do not account for candidate selection or multiple comparisons. They provide neither selection-adjusted significance nor independent validation.

*Ventricular-CSF contextualization*. Ventricular-CSF cells from the same source cohort were processed with the same quality-control and Harmony/Louvain framework, using 30 components and resolution 0.5. Candidate classical-monocyte clusters were selected without IFN genes, using CD14, S100A8, S100A9, S100A12, VCAN, and FCN1; clusters meeting the relative marker-score threshold of 0.75 of the maximum were retained. We applied the frozen AUCell transfer score using the selector-excluded positive signature and the original negative signature, with aucMaxRank = 1,500. The transferred signature comprised 135 positive and 150 negative genes. A sensitivity analysis removed overlapping monocyte-selection genes, leaving the positive set unchanged and removing S100A8 from the negative set. We compared the original and modified CSF scores and examined lineage-marker panels across the CSF clusters. A targeted sensitivity analysis additionally excluded cluster 13 because it showed greater T-cell and natural-killer-cell marker-panel expression than cluster 1. The 15-gene selector and stored IFN-high threshold were transferred without recalibration. Interpretation emphasized the six canonical donor pairs with at least 30 recovered CSF candidate cells; the complete nine-pair analysis was retained as supplementary context. Paired donor-median score differences were evaluated using two-sided Wilcoxon signed-rank tests with a normal approximation and continuity correction (exact = FALSE; correct = TRUE). *P*-values were nominal and unadjusted. Patient 6 contributed descriptive CSF information. Donor-level cell counts and conditional Wilson intervals were reported to make cell-yield limitations explicit. Effective donor number was calculated as the inverse sum of squared donor cell-contribution fractions; it describes concentration of cell recovery, not the number of biological replicates in the paired test.

## 3 Results

### 3.1 An IFN-response-high monocyte state is observed across the iNPH cohort

We obtained single-cell RNA sequencing data from the publicly available iNPH PBMC atlas (GSE292141) (Duy et al., 2025). Although raw data were available for ten patients, the canonical monocyte object used for the analysis contained nine donors and did not include Patient 6. Patient 6 also lacked publicly linked MoCA information, but missing cognitive information was not treated as a biological exclusion criterion and was evaluated separately in the ten-donor reconstruction. The nine canonical donors were described using the source-defined High-MoCA group (reported scores 23–29; *n* = 5) and Low-MoCA group (reported scores 19–20; *n* = 4). The narrow Low-MoCA range makes this a descriptive dichotomous comparison rather than a continuous cognitive analysis. After quality-control filtering, 122,724 high-quality cells were retained. The released canonical monocyte object comprised 22,197 classical monocytes (range, 1,343–4,541 per patient), which defined the canonical population; the uniform ten-donor reconstruction was evaluated separately. For PBMC-level visualization in Supplementary Figure 1, these exact cell identifiers were mapped onto the reconstructed PBMC embedding. Their classical-monocyte identity was supported by high expression of CD14, LYZ, S100A8, S100A9, VCAN, and FCN1 and near-absent expression of T-cell (CD3D, CD3E), B-cell (CD19, MS4A1), natural killer-cell (GNLY, NKG7), and dendritic-cell (FCER1A) markers (Supplementary Figure 1B). The wide inter-patient range in monocyte recovery was accommodated by pseudobulk library-size normalization and the paired donor design; effects of unequal cell recovery on precision cannot be excluded.

Subclustering of the 22,197 classical monocytes at resolution 0.5 resolved seven transcriptionally distinct subpopulations. Scoring each subcluster against a canonical type I interferon-stimulated gene (ISG) panel comprising IFITM3, IFITM2, ISG15, MX1, OAS1, IRF7, and STAT1 revealed one subcluster with markedly elevated IFN-response activity: subcluster 5 scored 1.28 versus ≤ 0.67 for all remaining subclusters (Figures 1A,B).

**FIGURE 1:**
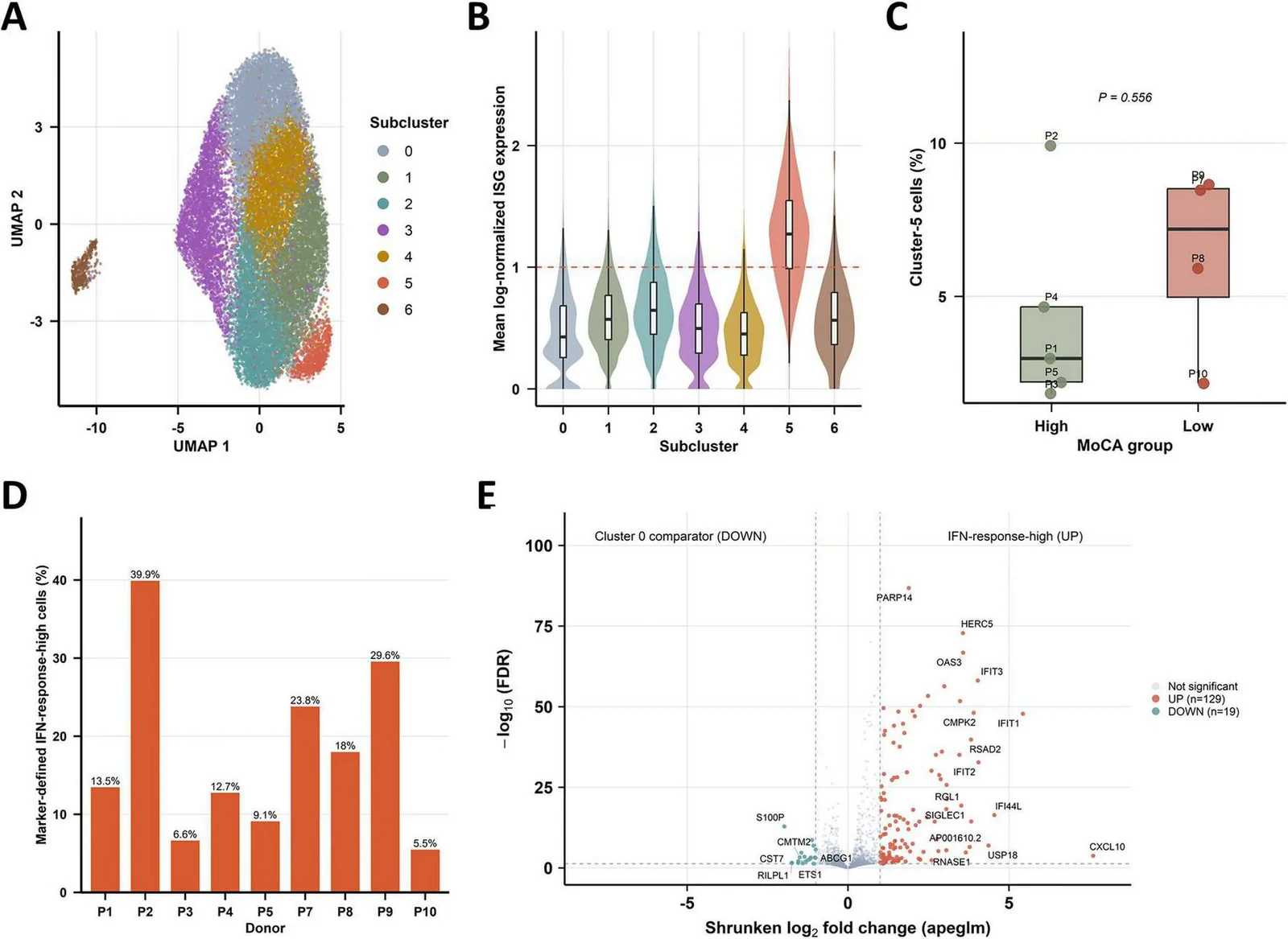
Characterization of an IFN-response-high monocyte state within iNPH. **(A)** UMAP of 22,197 canonical candidate monocytes from nine donors, colored by the Louvain subclusters at resolution 0.5. **(B)** Distribution of the mean log-normalized seven-gene IFN-panel score across subclusters; the panel comprises IFITM3, IFITM2, ISG15, MX1, OAS1, IRF7, and STAT1. The dashed line marks a score of 1.0 as a visual reference. **(C)** Donor-level frequency of cluster 5 in the source-reported High-MoCA (*n* = 5) and Low-MoCA (*n* = 4) groups. Each point represents a donor; the two-sided Wilcoxon rank-sum comparison was not statistically supported (nominal *P* = 0.556). **(D)** Percentage of canonical candidate monocytes meeting the frozen 15-gene marker-high rule within each donor. The rule classified 3,195 of 22,197 cells (14.4% pooled) as IFN-response-high. All nine donors met the ≥ 30-cell denominator criterion. **(E)** Paired pseudobulk comparison of cluster 5 with cluster 0 using DESeq2 design ∼ patient + state and apeglm shrinkage. Points represent genes; dashed lines mark FDR = 0.05 and shrunken log_2_ fold changes of ± 1. Colored points meet both FDR < 0.05 and —shrunken log_2_ fold change— > 1 (UP, *n = 129*; DOWN, *n = 19*); grey points include FDR-significant genes below the fold-change threshold. The marker-defined state in D is the descriptive definition; the cluster contrast in E supplied PRIMARY_150 and is not a marker-high-versus-marker- low comparison.

The source-defined MoCA groups showed no statistically supported difference in donor-level cluster-5 frequency (two-sided Wilcoxon rank-sum test, *W* = 7, nominal *P* = 0.556; Figure 1C). The small group comparison and unavailable exact donor-linked scores do not establish an association with cognition; neither do they establish equivalence between groups. Available donor characteristics and cell counts are provided in Supplementary Table 1.

Applying the frozen 15-gene marker rule to the canonical monocyte population identified 3,195 IFN-response-high cells among 22,197 monocytes (14.4% pooled across donors). Marker-high cells were detected in all nine donors, with donor-level frequencies shown in Figure 1D. This marker-based definition provided a broader descriptive characterization of the state, while the cluster-5-versus-cluster-0 contrast remained the source of the primary drug query and the marker-defined contrast was evaluated as a sensitivity analysis.

Alternative resolutions changed the size and membership of the selected IFN-response-high cluster, indicating that discrete cluster boundaries depended on the clustering specification (Supplementary Figures 2A,B). The uniform ten-donor reconstruction yielded 24,703 candidate monocytes and closely reproduced the canonical population among the nine shared donors (Jaccard overlap, 0.955). Patient 6 contributed 2,004 candidates, including 567 cells meeting the frozen marker-high rule (Supplementary Figure 2C). This sensitivity analysis retained Patient 6 without requiring cognitive metadata and was carried through to candidate connectivity analysis.

### 3.2 The cluster contrast defines an IFN-related cell-state query

To derive the within-iNPH IFN-response-high monocyte cell-state signature, we compared IFN-response-high cluster 5 (940 cells) with the low-IFN cluster-0 comparator (4,904 cells) using donor-paired pseudobulk differential expression in DESeq2 (∼ patient + state) and apeglm log_2_ fold-change shrinkage. This design accounts for within-patient correlation across cell states by treating each patient as a paired observation. The analysis identified 1,231 significant genes at FDR <0.05, comprising a biologically coherent landscape dominated by the type I interferon response (Figure 1E and Supplementary Table 2).

The most significantly upregulated gene was PARP14 (padj ≈ 1.58 × 10^–87^), encoding a type I IFN-inducible poly (ADP-ribose) polymerase with established roles in JAK/STAT-mediated macrophage activation. Genes with higher expression included HERC5, OAS3, IFIT3, ISG15, CMPK2, IFIT1, RSAD2, MX1, IFI6, IFIT2, LY6E, SIGLEC1, CXCL10, and USP18, consistent with an IFN-related transcriptional program. The final PRIMARY_150 query comprised the top 150 significant genes in each direction, ranked by shrunken log_2_ fold change. The leading positive and negative genes are displayed across the 18 donor-state pseudobulk samples in Supplementary Figure 3.

### 3.3 Signature scoring and external datasets provide descriptive contextualization

To examine how the signature was distributed across the canonical monocyte population, we compared scores for all 22,197 monocytes with those for the 940 cluster-5 cells. The mean composite score was −0.101 across all monocytes and +0.129 within cluster 5 (Supplementary Figure 4). The lower pooled score illustrates how the cluster-associated signal is diluted when the full monocyte population is considered. Because the all-monocyte population includes cluster 5 and these cells contributed to signature derivation, this comparison is descriptive rather than an independent validation.

To contextualize the magnitude of the iNPH ISG-high monocyte program, we compared the complete, unfiltered cluster 5 population with interferon-high classical monocytes from patients with systemic lupus erythematosus (SLE) in the Perez atlas (Perez et al., 2022). The primary AUCell analysis excluded the 15 genes used to define interferon-high cells from the scoring signature. The iNPH subcluster showed positive signature activity but lower activity than interferon-high SLE monocytes (median donor score, 0.0307 versus 0.0702; *n* = 9 iNPH and 143 SLE donors; two-sided Wilcoxon rank-sum test, nominal unadjusted *P* = 6.4 × 10^–6^). A mean-difference sensitivity analysis produced the same qualitative result (0.0725 versus 0.1886; *n* = 9 iNPH and 139 SLE donors; two-sided Wilcoxon rank-sum test, nominal unadjusted *P* = 1.5 × 10^–6^; Figure 2A and Supplementary Table 3). Healthy-control donors (*n* = 99) are shown as a descriptive reference in Figure 2A, with summary statistics in Supplementary Table 3. Within the current analytical framework, the selected iNPH population had lower signature scores than the selected SLE population. Differences in cell ascertainment and the confounding of diagnosis with dataset prevent interpretation of these differences as a measure of biological severity or disease specificity.

**FIGURE 2:**
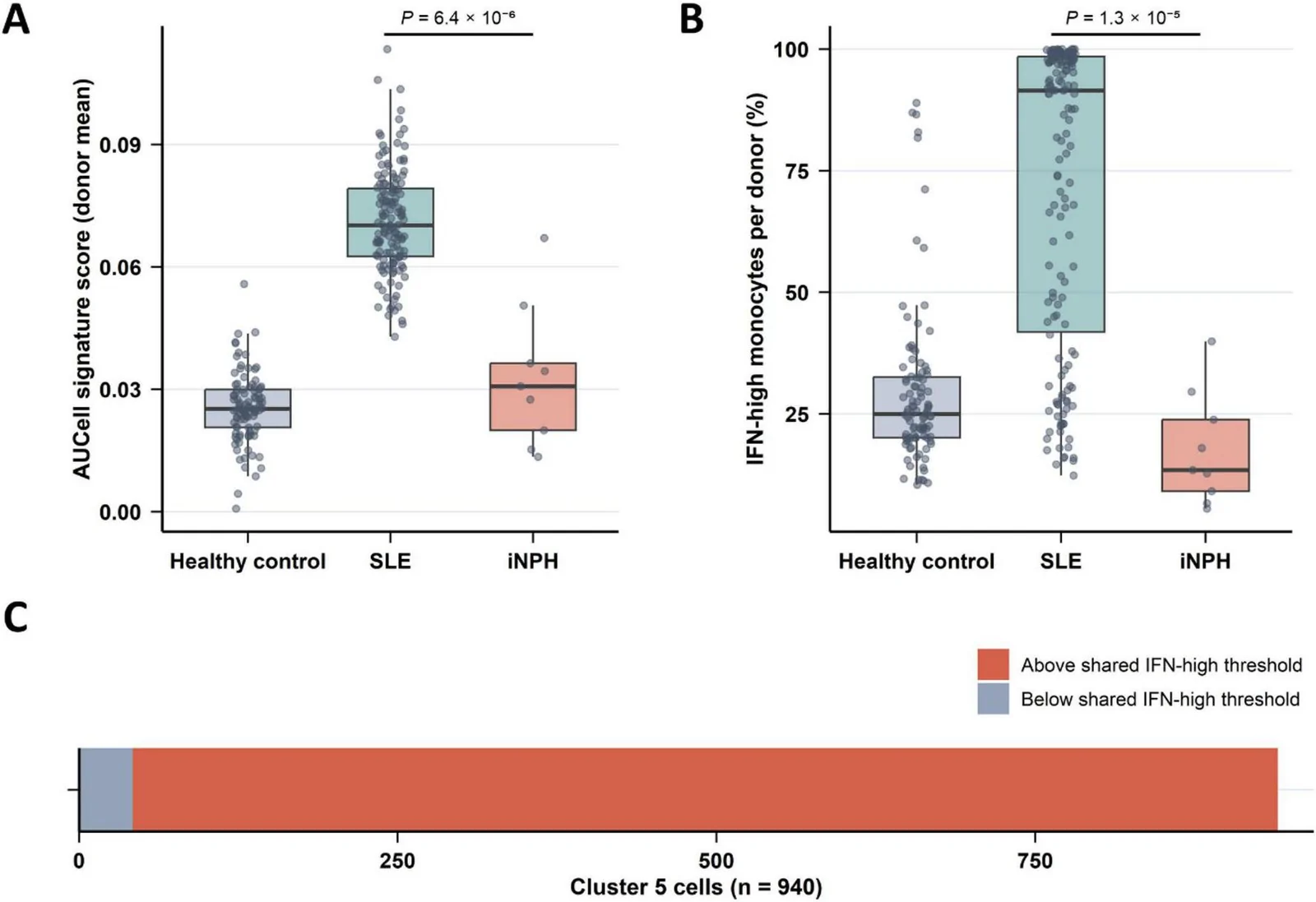
External contextualization and marker–cluster concordance. **(A)** Donor-level AUCell signature scores for the complete iNPH cluster 5 and externally selected IFN-high classical monocytes from the Perez SLE/healthy-control atlas. The 15 selector genes were excluded from the positive scoring signature. External IFN-high cells were selected separately within SLE and healthy-control groups, whereas iNPH cluster 5 was retained without further selection. Points represent donors (iNPH, *n* = 9; SLE, *n* = 143; healthy controls, *n* = 99). **(B)** Donor-level percentages above the shared donor-balanced healthy-control-anchored IFN-selector threshold, applied to the full monocyte populations (iNPH, *n* = 9; SLE, *n* = 162; healthy controls, *n* = 99). Boxes show medians and interquartile ranges; displayed *P*-values are from two-sided Wilcoxon rank-sum tests and are nominal. **(C)** Marker-high status within the 940 cluster-5 cells: 898 cells (95.5%) met the frozen AUCell rule. These cells constitute 28.1% of the broader 3,195-cell marker-high population. The panels describe related but nonidentical populations. Selection differences, external threshold calibration, and dataset confounding preclude an iNPH-specific interpretation; alternative scoring results are reported in Supplementary Table 3.

Using a shared, donor-balanced, healthy-control–anchored threshold, IFN-high monocyte burden was substantially lower in iNPH (*n* = 9 donors) than in SLE (*n* = 162 donors) under both scoring methods. AUCell estimated burdens of 13.5% in iNPH and 91.5% in SLE (two-sided Wilcoxon rank-sum test, nominal unadjusted *P* = 1.3 × 10^–5^), whereas mean-difference scoring estimated 30.1% and 91.8%, respectively (nominal unadjusted *P* = 0.001). The comparison with healthy controls (*n* = 99 donors) was scoring-method-dependent: AUCell estimated lower burden in iNPH than in healthy controls (13.5% versus 25.0%; nominal unadjusted *P* = 0.013), whereas mean-difference scoring did not support a difference (30.1% versus 25.0%; nominal unadjusted *P* = 0.49). We therefore treated the healthy-control comparison as exploratory and did not infer either increased or decreased IFN-high burden in iNPH (Figure 2B and Supplementary Table 3).

Marker-based thresholding supported the IFN-response-high annotation of cluster 5. Using the shared donor-balanced AUCell threshold, 95.5% of cluster-5 cells met the marker-high rule; the corresponding value was 99.5% using mean-difference scoring. Conversely, only 28.1% of the broader AUCell marker-high population belonged to cluster 5. Cluster 5 therefore represents a subset of the broader marker-defined state, and the two definitions should not be treated as equivalent (Figure 2C).

### 3.4 Primary THP1 connectivity screening and *post hoc* candidate selection

The primary query returned THP1 scores for 1,376 compounds, including 779 exact-zero and 597 nonzero NCS values. Among the nonzero results, 288 had NCS <−0.5 and 138 also had WTCS FDR <0.05. Figure 3A displays the nonzero distribution while explicitly accounting for the omitted zero values; all 1,376 entries were retained in ranking. The NCS cutoff defines an operational screening rule rather than a validated therapeutic threshold.

**FIGURE 3:**
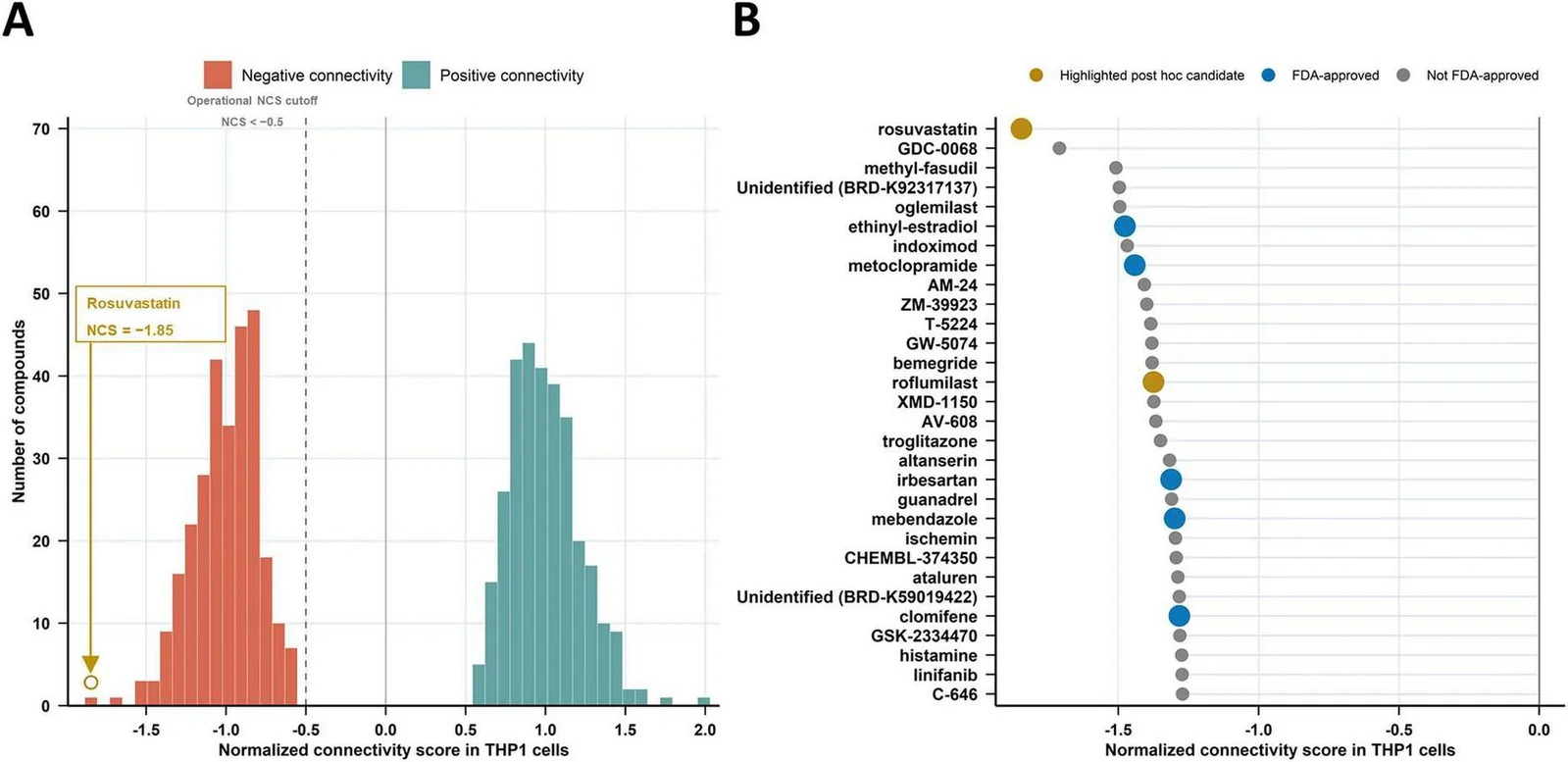
Primary THP1 connectivity screen for the within-iNPH cell-state signature. **(A)** Distribution of NCS values for the 597 compounds with nonzero THP1 connectivity. The 779 exact-zero entries among all 1,376 compounds are omitted from the histogram but retained in numerical accounting and ranking. NCS = 0 is not interpreted as biological neutrality. Rosuvastatin is highlighted at NCS approximately −1.85. The dashed vertical line marks −0.5, the heuristic cutoff used in the operational screening rule. **(B)** The 30 most negatively connected compounds in the PRIMARY_150 query. Colors identify rosuvastatin and roflumilast as the compounds selected for focused discussion, other compounds annotated as FDA-approved, and the remaining compounds under the reported regulatory classification. Selection of the two highlighted compounds was *post hoc* and does not imply that roflumilast was the second-ranked compound. Negative connectivity is a computational prediction rather than experimentally demonstrated reversal or clinical efficacy.

Rosuvastatin ranked first in the primary THP1 screen (NCS = −1.845; WTCS = −0.487; FDR = 6.14 × 10^–4^). Roflumilast ranked 14th (NCS = −1.375; FDR = 6.14 × 10^–4^). Roflumilast was selected for focused discussion after the screen because its PDE4 pharmacology provided a complementary experimental hypothesis, not because it was the second-ranked compound or had demonstrated superiority over higher-ranked compounds (Figure 3B).

Five additional FDA-approved compounds occurred among the top 30: ethinyl estradiol, metoclopramide, irbesartan, mebendazole, and clomifene. These were retained as other approved predicted reversal candidates but were not assigned the same highlighted status as rosuvastatin and roflumilast. Selected compounds are summarized in Table 1, and the annotated top-30 primary ranking, together with the separate baricitinib entry at rank 39, is presented in Supplementary Table 4.

**TABLE 1:** Selected approved compounds from the primary THP1 connectivity screen and *post hoc* mechanistic review.

Primary THP1 rank	Integrated robustness rank	Qualifying core queries	Compound	BRD ID	THP1 NCS	WTCS	FDR	Regulatory status	Pharmacologic class	Reporting category
1	1	11/11	Rosuvastatin	BRD-K82941592	−1.845	−0.487	6.14 × 10^–4^	Approved (FDA)	HMG-CoA reductase inhibitor (statin)	Highlighted *post hoc* candidate
6	4	11/11	Ethinyl estradiol	BRD-A02367930	−1.477	−0.390	6.14 × 10^–4^	Approved (FDA; ingredient)	Estrogen (synthetic)	Other approved predicted reversal candidate
8	169	5/11	Metoclopramide	BRD-K75641298	−1.442	−0.380	6.14 × 10^–4^	Approved (FDA)	Dopamine D2 receptor antagonist	Other approved predicted reversal candidate
14	15	10/11	Roflumilast	BRD-K03194791	−1.375	−0.363	6.14 × 10^–4^	Approved (FDA)	Phosphodiesterase-4 (PDE4) inhibitor	Highlighted *post hoc* candidate
19	8	10/11	Irbesartan	BRD-K60038276	−1.312	−0.346	6.14 × 10^–4^	Approved (FDA)	Angiotensin II receptor blocker	Other approved predicted reversal candidate
21	40	10/11	Mebendazole	BRD-K77987382	−1.299	−0.343	6.14 × 10^–4^	Approved (FDA)	Benzimidazole anthelmintic (microtubule inhibitor)	Other approved predicted reversal candidate
26	363	4/11	Clomifene	BRD-K29950728	−1.282	−0.338	6.18 × 10^–4^	Approved (FDA)	Selective estrogen receptor modulator	Other approved predicted reversal candidate
39	68	6/11	Baricitinib	BRD-K53581288	−1.231	−0.325	8.17 × 10^–4^	Approved (FDA/EMA)	Janus kinase 1/2 (JAK1/2) inhibitor	*Post hoc* secondary candidate

Primary rank refers to the PRIMARY_150 THP1 query. Integrated robustness rank summarizes placement across the 11 core queries using the procedure described in Materials and methods section “2.11 Robustness assessment.” Qualifying core queries met both NCS <−0.5 and saved WTCS FDR <0.05. Reporting categories were assigned after inspection of the screen and are not prospective clinical-prioritization criteria. Approval refers to established non-iNPH indications. Complete query-level results are provided in Supplementary Data 2.

### 3.5 Candidate prioritization varies across analytical choices, with stronger consistency for rosuvastatin

We completed a total of 121 queries: one primary query, ten biological sensitivity queries, nine donor-omission queries, one Hallmark query, and 100 matched-null queries. Across the 11 core analyses, rosuvastatin had a median average-tie rank of 2 (range, 1–73) and met NCS <−0.5 and WTCS FDR <0.05 in all 11 analyses. Roflumilast had a median rank of 35 (range, 8–713) and met the same criteria in ten analyses; it returned NCS = 0 for the resolution-0.7 contrast (Figure 4A). The integrated ranking placed rosuvastatin first and roflumilast 15th. Thus, the robustness assessment supported continued experimental interest in both compounds while distinguishing roflumilast’s less consistent placement. The zero at resolution 0.7 is an algorithmic connectivity result, not evidence that the compound is biologically inactive under that state definition.

**FIGURE 4:**
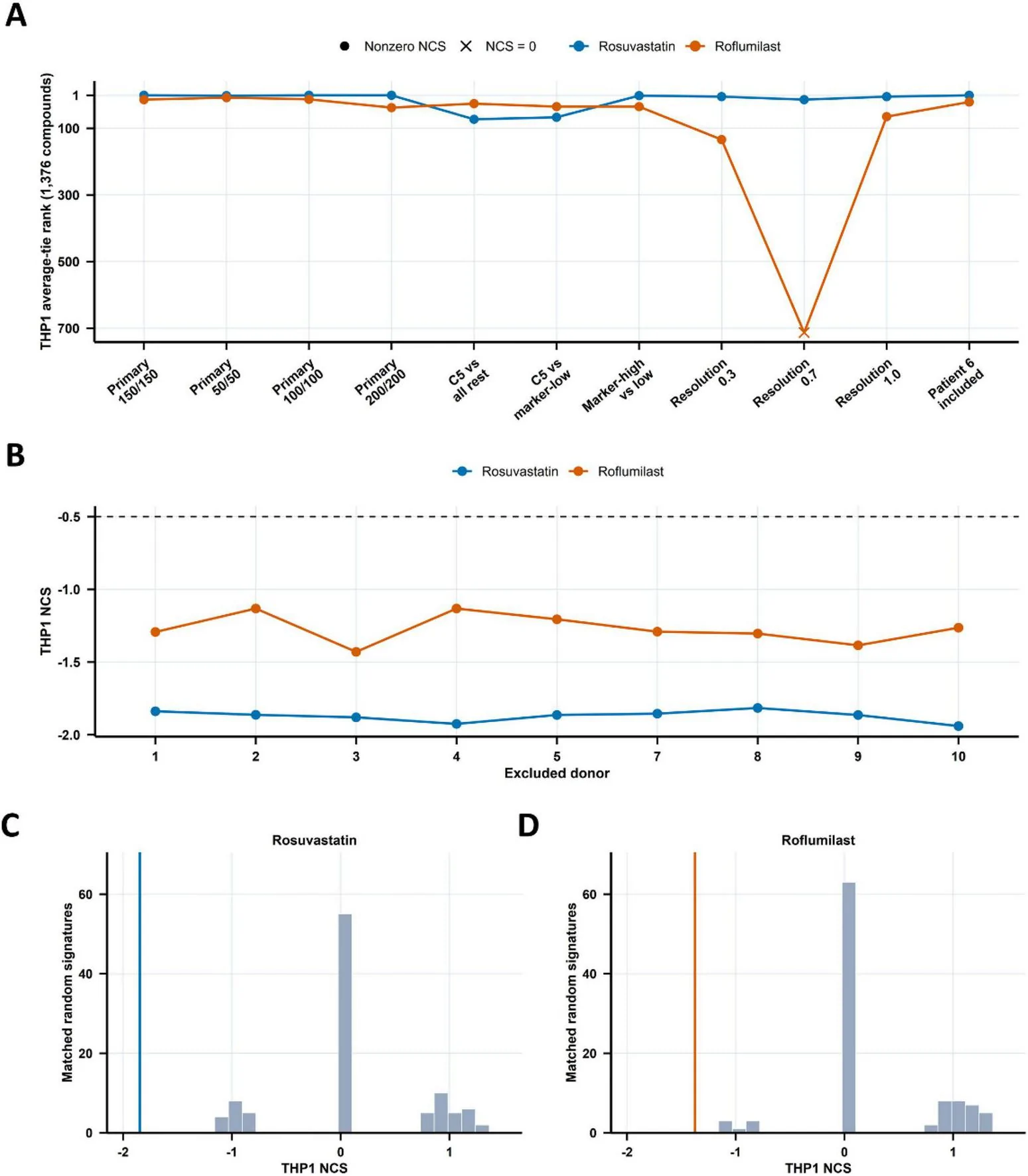
Sensitivity of candidate prioritization and comparison with expression-matched null signatures. **(A)** Query-specific average-tie THP1 ranks of rosuvastatin and roflumilast across the 11 core analyses, including signature-size, comparator, marker-definition, clustering-resolution, and Patient 6 sensitivities. Lower ranks indicate stronger relative inverse connectivity among 1,376 compounds. The marked zero result for roflumilast at resolution 0.7 is retained with its average tied rank. These are per-query ranks, not the integrated rank itself. **(B)** THP1 NCS values after each of the nine conditional donor omissions. The state labels were retained; the dashed line marks the operational NCS cutoff of −0.5. **(C)** Rosuvastatin and **(D)** roflumilast PRIMARY_150 NCS values, shown as colored vertical lines, against distributions from the same 100 expression-matched null queries. For each compound, no null score was as negative as the observed score; the plus-one lower-tail empirical probability is 1/101. These exploratory, post-selection comparisons are not adjusted for candidate selection or multiple comparisons and do not establish independent validation, disease specificity, IFN exclusivity, or therapeutic efficacy.

Across the nine conditional donor omissions, rosuvastatin retained ranks 1–2, with median NCS −1.864 (range, −1.940 to −1.816). Roflumilast retained ranks 10–77, with median NCS −1.291 (range, −1.430 to −1.132). Both met the operational NCS/FDR criteria in all nine queries (Figure 4B). These findings demonstrate downstream candidate consistency conditional on the retained state labels; they do not show that the complete state-discovery pipeline is stable to every donor omission. Candidate-level NCS, average-tie ranks, and saved WTCS FDR values for all nine donor omissions are provided in Supplementary Data 2, with the integrated top-20 robustness summary in Supplementary Table 5.

Neither compound’s observed PRIMARY_150 NCS was reached or exceeded in the negative direction by the 100 expression-matched null queries, giving a nominal lower-tail empirical probability of 1/101 (approximately 0.0099) for each compound (Figures 4C,D). This shows that the observed scores were more negative than the sampled matched-null scores for these selected compounds. It does not establish disease specificity, selectivity for IFN over other inflammatory programs, or superiority over every other compound. The empirical probabilities were not multiplicity-adjusted. Because the compounds were selected after inspection of the primary screen, these comparisons do not provide selection-adjusted significance or independent validation.

### 3.6 Conditional stability of the gene signature

Conditional leave-one-patient-out refitting showed overall stability of the within-iNPH cell-state signature to individual-patient exclusion while retaining the fixed cluster labels. Differential expression analysis was repeated nine times with one patient removed per iteration; each run retained 940–1,098 significant genes at FDR <0.05. Mean Jaccard similarity to the full nine-patient signature was 0.714 for upregulated genes and 0.733 for downregulated genes, yielding an overall mean of 0.724, relative to the plotted visual reference of 0.70, although individual leave-one-out mean similarities ranged from 0.658 to 0.766 (Supplementary Figure 5A). Gene-level persistence analysis focused on the original 150-gene upregulated component of the signature. Of these genes, 137 were recovered in the iteration-specific top-150 upregulated set in at least one leave-one-out analysis, and 107 were recovered in all nine iterations (Supplementary Figure 5B), supporting a substantial stable core within the upregulated interferon program. Because Harmony integration, clustering, and cluster identification were not repeated, this analysis evaluates conditional robustness of the differential signature rather than the stability of the entire single-cell pipeline.

### 3.7 Connectivity depends on the perturbational cell context

Rosuvastatin was represented in 22 LINCS2 cell lines and roflumilast in 15. Each compound showed its most negative PRIMARY_150 NCS in THP1, but inverse connectivity was not uniform across the other cell contexts (Figure 5). Rosuvastatin showed significant negative connectivity in six cell lines and significant positive connectivity in one; roflumilast showed significant negative connectivity in THP1 and two other lines and significant positive connectivity in three. These profiles demonstrate context-dependent computational connectivity rather than a universal compound effect.

**FIGURE 5:**
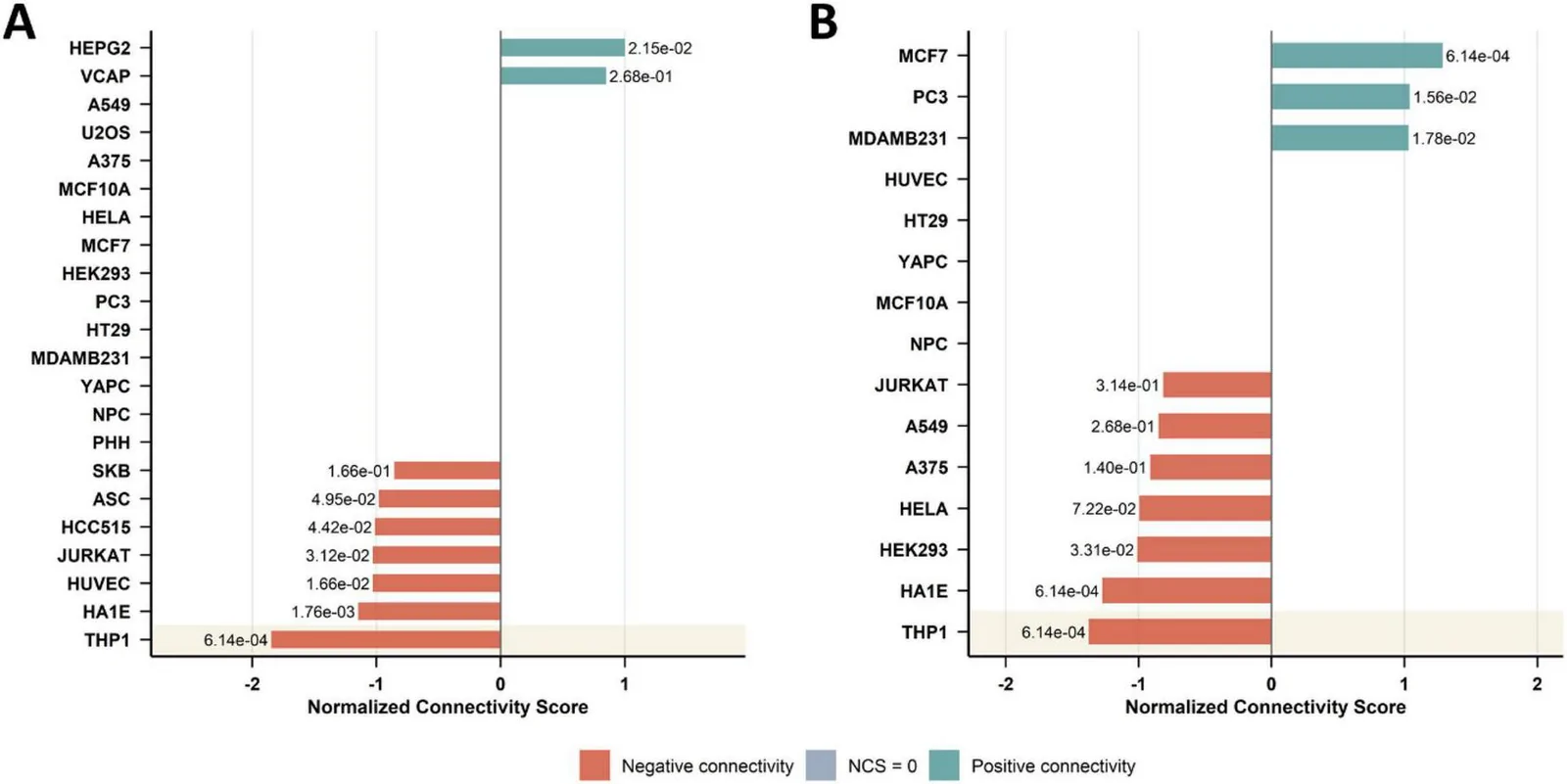
Cell-line dependence of rosuvastatin and roflumilast connectivity. PRIMARY_150 NCS values for panel **(A)** rosuvastatin (BRD-K82941592) across 22 cell lines and **(B)** roflumilast (BRD-K03194791) across 15 cell lines represented in the executed LINCS2 results. Negative and positive bars indicate inverse and positive transcriptional connectivity, respectively; exact zeros are displayed without inferring biological neutrality. Labels show the saved WTCS FDR for each nonzero connectivity score, including values ≥ 0.05. THP1, the transformed monocytic screening context, is highlighted. Both compounds have their most negative displayed score in THP1, but direction and significance vary across cell contexts. The results do not demonstrate drug responses in primary patient monocytes.

A separate *post ho*c review identified baricitinib, an approved JAK1/JAK2 inhibitor with potential relevance to IFNAR-associated JAK signaling. Baricitinib ranked 39th in the primary THP1 screen and showed significant inverse connectivity in THP1 (NCS = −1.23; FDR = 8.17 × 10^–4^; Supplementary Figure 6). Because it was identified outside the original top-30 review, baricitinib is presented as a *post hoc* mechanistically aligned secondary candidate, not as a primary candidate or evidence of JAK-inhibitor class enrichment.

### 3.8 Hallmark interferon-α pathway sensitivity shows compound-specific connectivity

The non-overlapping Hallmark IFN-α-response sensitivity used 49 LINCS-represented genes among the 54 genes remaining after exclusion of all overlap with PRIMARY_150 (Liberzon et al., 2015). Rosuvastatin and roflumilast retained significant negative connectivity (NCS = −2.243 and −1.250, respectively; FDR = 1.04 × 10^–4^ for each). Baricitinib had negative connectivity but did not meet FDR <0.05 (NCS = −0.777; FDR = 0.0844). Results varied among the selected JAK inhibitors and other reported compounds (Supplementary Table 6). This related-pathway sensitivity does not establish IFN exclusivity, class-wide activity, disease specificity, or independent validation.

### 3.9 The blood-derived program is detectable in ventricular-CSF monocyte candidates

The blood-derived program was detectable in ventricular-CSF candidate monocytes selected without IFN markers. Among the six matched donors with at least 30 recovered CSF candidates, all donor-median CSF-minus-PBMC transfer-score differences were positive (median difference, 0.04105; range, 0.03014–0.07069; Wilcoxon *V* = 21; nominal *P* = 0.0360). The complete nine-pair analysis also showed positive differences in every donor (median difference, 0.04130; *V* = 45; nominal *P* = 0.00915), but included donors with very low cell recovery, including one candidate cell from Patient 5 (Supplementary Figure 7A).

Clusters 1 and 13 met the classical-monocyte marker-selection criterion, although cluster 13 showed greater T-cell and natural-killer-cell marker-panel expression. Excluding cluster 13 retained the same six eligible donor pairs and positive differences in all six (median difference, 0.04223; nominal *P* = 0.0360). Removing S100A8, the only monocyte-selection gene overlapping the transferred signature, preserved CSF cell-score ordering closely (Spearman ρ = 0.99953 across 2,683 cells). This sensitivity assessed CSF score stability; modified CSF scores were not compared with unmodified PBMC scores.

Cell recovery remained markedly unequal, ranging from 1 to 2,069 candidates per donor. Patient 10 supplied 77.12% of selected CSF cells, corresponding to an effective donor number of 1.64 across the ten donors represented descriptively (Supplementary Figures 7B,C). The findings support detectability of the transferred program in selected CSF cells, while annotation uncertainty, unequal recovery, and differences in cell composition and assay context limit inference about prevalence, compartmental enrichment, or CNS origin. Annotation diagnostics, transferred gene sets, donor-level differences, and sensitivity results are provided in Supplementary Data 1.

## 4 Discussion

In this study, we characterized an IFN-response-high classical-monocyte state observed within an iNPH cohort and used its cluster-derived transcriptional contrast for computational compound prioritization. Most cluster-5 cells met the frozen marker-high rule, although cluster 5 captured only a subset of the broader marker-defined population. Sensitivity analyses evaluated alternative state definitions, comparators, signature sizes, clustering resolutions, Patient 6 inclusion, donor omission, and expression-matched null signatures. Cross-dataset analysis indicated lower IFN-response activity and burden than in SLE, while the healthy-control comparison was scoring-method-dependent. Taken together, these analyses connect a contextually qualified monocyte signal to experimentally testable drug hypotheses: LINCS2 mapping highlighted rosuvastatin and roflumilast, with stronger computational consistency for rosuvastatin, while baricitinib was retained as a *post hoc* secondary candidate.

This computational prioritization is driven by an important therapeutic gap in iNPH, as shunting remains the only established disease-directed treatment and no approved pharmacologic therapy addresses persistent symptoms. The PENS trial demonstrated that shunting improved gait velocity and gait and balance at 3 months in patients selected for responsiveness to temporary CSF drainage, whereas measured cognitive and urinary outcomes did not differ significantly at that time point (Luciano et al., 2025). This domain-specific result does not show that shunting fails overall or establish a CSF-independent cause of cognitive impairment, but it highlights an unmet need for adjunctive strategies addressing biological processes that may persist after CSF diversion. At this hypothesis-generating stage, the present study therefore identifies a testable inflammatory signature and candidate compounds without establishing that the state causes cognitive impairment or that the compounds will improve cognition.

Interpreting this therapeutic hypothesis first requires placing the underlying monocyte state in biological context. The identification of an IFN-response-high monocyte state is consistent with broader evidence implicating chronic interferon signaling in age-associated neurological dysfunction and with recent clinical reports of peripheral immune and inflammatory alterations in iNPH (Bissacco et al., 2024; Yang et al., 2025). The original iNPH single-cell study reported broader proinflammatory monocyte alterations associated with cognitive status; our analysis describes a specific interferon-associated state but does not establish that this state explains the reported cognitive association. Cross-dataset contextualization showed lower signature activity in iNPH cluster 5 and lower IFN-response-high burden across the full canonical iNPH monocyte population than in the corresponding SLE comparisons. The comparison with healthy controls was not stable across scoring methods and should not be used to infer either increased or decreased IFN-response-high burden in iNPH. These results establish descriptive context, not iNPH specificity.

The biological origin of this peripheral signal remains uncertain: it may reflect a response to CNS- or CSF-derived stimuli rather than an initiating process in iNPH. The source study described immune alterations in both compartments (Duy et al., 2025), and experimental work has linked aged-CSF signals to choroid-plexus IFN responses (Baruch et al., 2014). Our CSF analysis supports detectability of the blood-derived program at the CNS interface but cannot establish the direction of signaling or distinguish causal, amplifying, compensatory, and biomarker roles. Longitudinal sampling before and after CSF drainage or shunting, together with patient-derived monocyte experiments, will be needed to distinguish these possibilities.

Although its causal role remains unresolved, the state’s transcriptional profile provides a testable target for perturbational screening. The two highlighted candidates provide complementary but hypothesis-generating pharmacological signals. Rosuvastatin ranked first in the primary THP1 screen and first in the integrated robustness analysis (Figures 3B, 4 and Table 1). Its strongest displayed cell-line inverse connectivity occurred in THP1, although connectivity was not uniformly negative across cell contexts (Figure 5A). Statins have documented immunomodulatory effects in monocytes and can alter isoprenoid-dependent small-GTPase signaling, providing a rationale for testing rosuvastatin against the within-iNPH IFN-response-high signature in patient-derived cells (Funderburg et al., 2015; Oesterle et al., 2017). Roflumilast ranked 14th in the primary screen and 15th in the integrated analysis, with less consistent placement across the core analyses and variable cell-line connectivity (Figures 4, 5B). Studies of PDE4 inhibition provide context for possible anti-inflammatory and cognitive effects but do not establish efficacy in iNPH (Chen et al., 2025; Feng et al., 2019; Prickaerts et al., 2024; Spina, 2008). Baricitinib showed significant primary-query THP1 inverse connectivity but was selected through a separate *post hoc* mechanistic review and did not reach statistical significance in the Hallmark sensitivity analysis (Supplementary Figure 6 and Supplementary Table 6). Possible pathways through which rosuvastatin and roflumilast could influence IFN-associated transcription are summarized as a hypothetical model in Supplementary Figure 8. These findings nominate compounds for experimental testing rather than establish therapeutic efficacy, pathway causality, or class-wide pharmacological activity.

These interpretations are constrained by four main limitations. First, the within-iNPH cell-state signature was derived from a single publicly available dataset of nine patients. The cognitive stratification showed no evidence of a group difference (two-sided Wilcoxon rank-sum test, *P* = 0.556) and was based on a small cohort with a narrow MoCA score range clustered between 19 and 20 in the Low MoCA group; the cohort therefore approximates a dichotomous rather than ordinal cognitive comparison, a property of the source dataset rather than a choice in the present analysis. Individual patient-to-MoCA-score mappings are not reported in the source publication; accordingly, we did not perform a continuous MoCA correlation, and no association with cognition can be inferred from the group-level analysis.

Second, drug prioritization used LINCS profiles from THP1, a transformed leukemia-derived monocytic cell line, rather than primary monocytes from patients with iNPH. Source metadata recovered the exposure conditions and three constituent replicate profiles for each highlighted or secondary candidate. However, all three exemplar signatures had is_hiq = 0, and the rosuvastatin and baricitinib exemplars also had qc_pass = 0, indicating that fewer than half of their constituent replicate profiles passed the source processing-QC criteria. These reference-signature limitations qualify confidence in the compound predictions. Consistency across alternative queries does not resolve this limitation because the queries reuse the same reference signatures. Controlled perturbation experiments in donor-replicated primary monocytes are therefore required before therapeutic inference. Detection of the blood-derived program in CSF does not constitute drug-response validation.

Third, the cross-disease analyses compared independently generated and processed iNPH and Perez datasets. Both scoring methods yielded lower signature scores and lower estimated IFN-response-high fractions in iNPH than in SLE within the current analytical framework, whereas the iNPH-versus-healthy-control comparison differed between methods. We therefore treated the healthy-control comparison as exploratory and did not draw a directional biological conclusion from it.

Fourth, the robustness analyses test specified analytical choices but do not exhaust all possible integration, clustering, and state-definition procedures. The donor-omission analysis retained the labels and therefore measures conditional downstream stability rather than independent rediscovery of the state. The 100 expression-matched null queries provide exploratory comparisons for compounds selected after the primary screen; they do not provide selection-adjusted significance, independent validation, or specificity against all inflammatory programs. Finally, markedly unequal CSF candidate-cell recovery limits inference about compartmental enrichment, prevalence, and CNS origin.

## 5 Conclusion

To our knowledge, this study provides a systematic transcriptomics-driven compound-prioritization analysis focused on an IFN-response-high monocyte state observed in iNPH. By integrating marker-based state characterization, a cluster-derived query, alternative state and comparator definitions, signature-size and clustering-resolution sensitivities, Patient 6 inclusion, conditional donor-omission queries, matched-null comparisons, and blood–CSF contextualization, the analysis identifies rosuvastatin and roflumilast as experimentally testable candidates with predicted inverse connectivity. Computational support was more consistent for rosuvastatin. Future studies should determine whether the state is reproduced in independent iNPH cohorts, whether the nominated compounds modify the program in primary patient-derived monocytes, and whether the state changes after CSF drainage or shunting. These experiments are required before disease-specific, biomarker, cognitive, or therapeutic conclusions can be drawn.

## Data Availability

Publicly available datasets were analyzed in this study. The iNPH PBMC and ventricular-CSF single-cell RNA sequencing data (Duy et al., 2025) are available from the Gene Expression Omnibus under accession GSE292141. The systemic lupus erythematosus and healthy-control comparator data (Perez et al., 2022) are available from CZ CELLxGENE Discover (collection ID 436154da-bcf1-4130-9c8b120ff9a888f2). LINCS2 reference signatures were accessed through Bioconductor ExperimentHub (resource EH7297). LINCS2020 signature and compound metadata (siginfo_beta.txt and compoundinfo_beta.txt) are available from CLUE (https://clue.io/data). The Hallmark interferon-alpha response gene set (M5911) is available from MSigDB. All analysis code is available at https://github.com/KNU-BIST/iNPH-drug-repurposing and has been permanently archived in Zenodo at https://doi.org/10.5281/zenodo.21896723. No new data were generated in this study.

## Supplementary material

Supplementary Data 1 Ventricular-CSF contextualization of the blood-derived monocyte program: paired PBMC-CSF tests with six-pair and nine-pair sensitivity analyses, donor-level CSF summaries, cell-yield sensitivity with Wilson intervals, CSF cluster annotation diagnostics, selection-gene removal sensitivity, transferred gene sets and cell-level CSF scores.

Supplementary Data 2 Compound connectivity and candidate robustness: definitions, input genes and donor counts for all 121 queries; THP1 results for the 21 observed queries; all-compound and top-20 robustness summaries; donor-omission results; matched-null scores for rosuvastatin and roflumilast; LINCS2020 exemplar, condition and replicate metadata; and software and query-execution records.
