# Supplementary material for: Systems biology prioritizes drug candidates for an interferon-response-high monocyte state in idiopathic normal pressure hydrocephalus
Source: Front Aging Neurosci. 2026 Oct 23;18:1971059. doi: 10.3389/fnagi.2026.1971059 (PMC13756037; doi:10.3389/fnagi.2026.1971059)
Supplement: Supplementary file 1 [file Supplementary_file_1.pdf]

Supplementary Material

Supplementary Figures

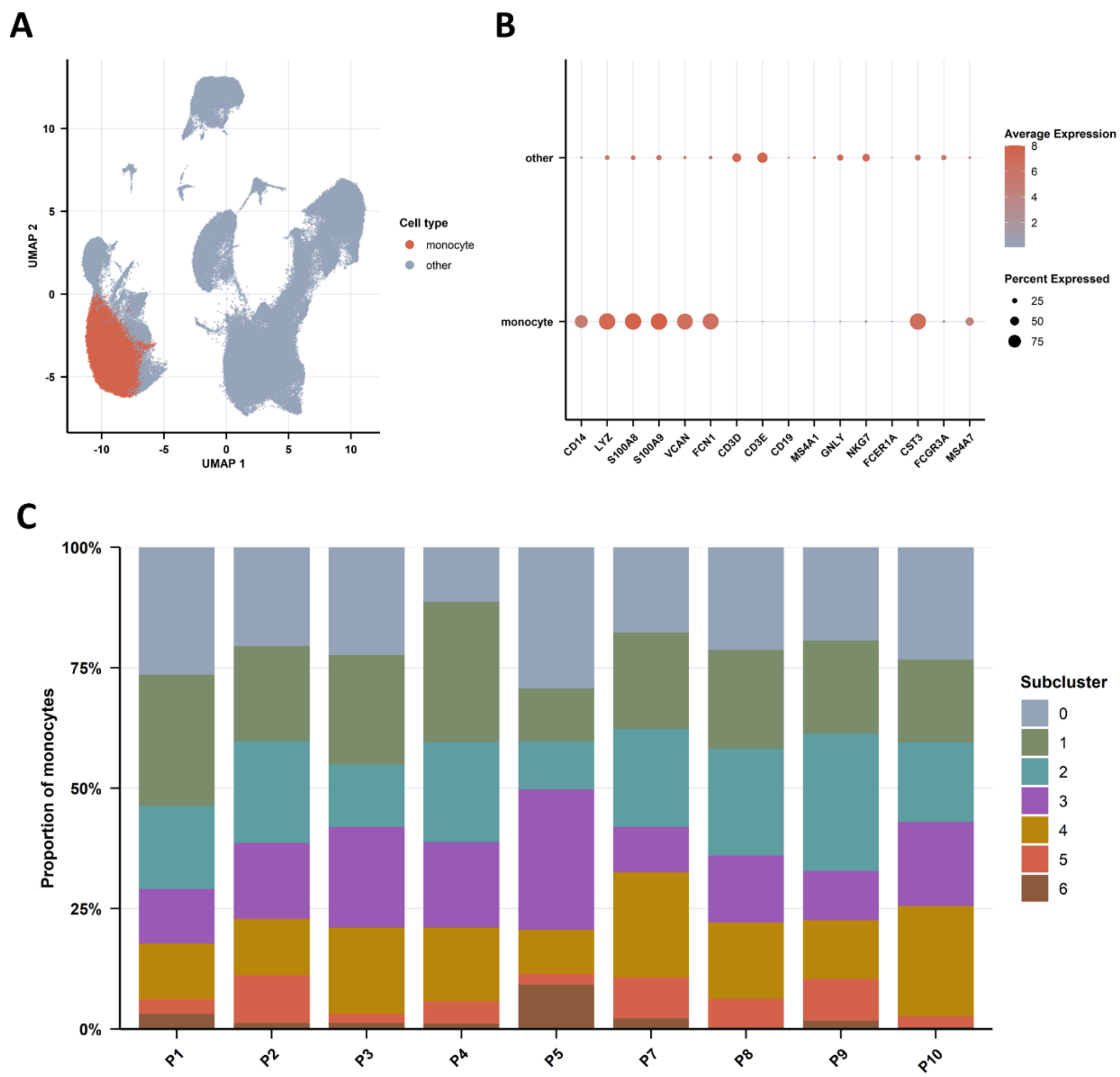

Supplementary Figure S1. PBMC annotation and donor-level monocyte subcluster composition. (A) Historical UMAP reconstruction of 122,724 PBMCs from nine iNPH donors after excluding cells with fewer than 200 detected genes or more than 20% mitochondrial transcripts. No three-median-absolute-deviation total-count filter was applied. The 22,197 exact canonical monocyte identifiers are highlighted; other cells are shown in gray. (B) Lineage-marker dot plot for canonical monocytes and the remaining cells. Dot size indicates the percentage expressing each gene, and color indicates average expression. (C) Relative composition of the seven monocyte subclusters within each donor, including cluster 5. Donor order and colors represent the displayed donor/subcluster composition and do not encode a separate MoCA grouping.

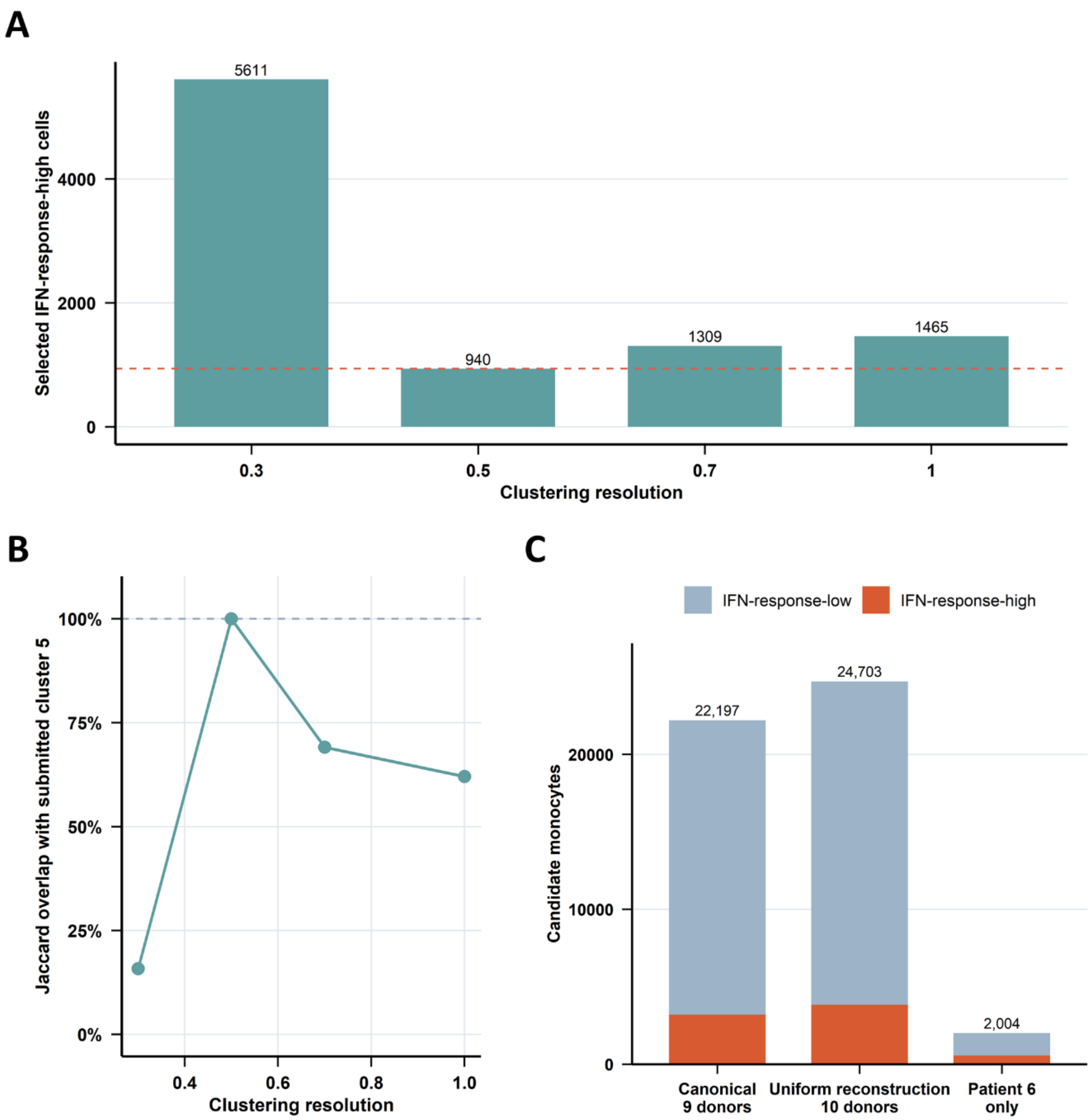

Supplementary Figure S2. Sensitivity of cell-state definition and candidate-monocyte reconstruction. (A) Number of cells assigned to the IFN-response-high cluster at Louvain clustering resolutions 0.3, 0.5, 0.7 and 1.0. The dashed horizontal line marks the 940-cell subcluster obtained at the resolution of 0.5. (B) Jaccard overlap between the cell population selected at each clustering resolution and subcluster 5. The dashed horizontal line marks complete overlap. These analyses evaluate the dependence of cluster membership on the selected resolution; the frozen marker-defined state remains the primary descriptive definition, while cluster 5 versus cluster 0 remains the source of the original drug query. (C) Numbers of marker-defined IFN-response-high and IFN-response-low candidate monocytes in the canonical nine-donor analysis, the uniform ten-donor reconstruction and Patient 6 alone. The uniform reconstruction contained 24,703 candidate monocytes and closely reproduced the canonical population among the nine shared donors (Jaccard = 0.955). Patient 6 contributed 2,004 candidates, of which 567 met the frozen IFN-response-high rule. Patient 6 inclusion is presented as a sensitivity analysis and does not retroactively alter the canonical nine-donor analysis.

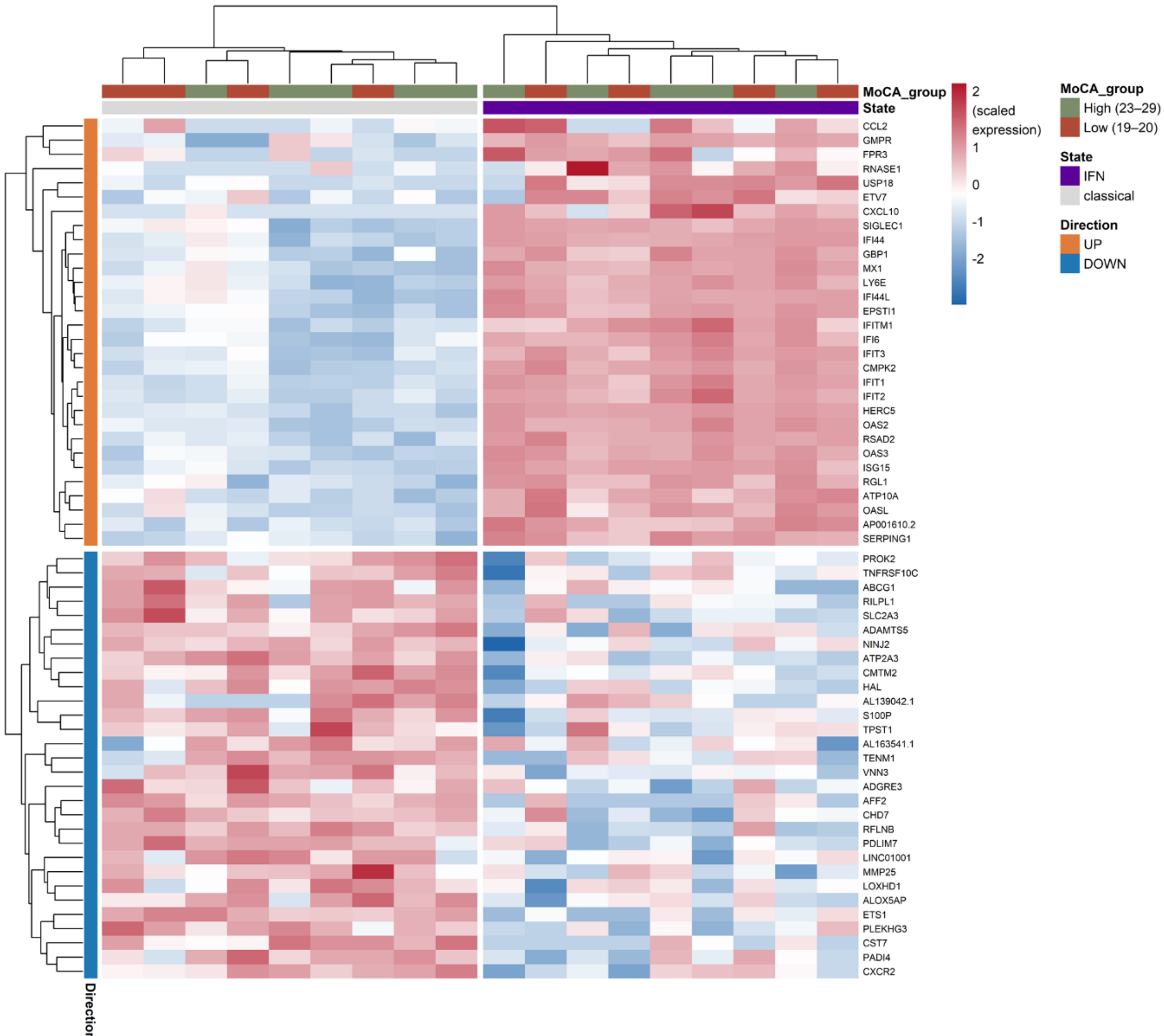

Supplementary Figure S3. Leading genes in the cluster-derived signature. Heatmap of the top 30 positive and top 30 negative genes ranked by shrunken log2 fold change in the cluster-5-versus-cluster-0 contrast. Columns represent the 18 donor-state pseudobulk samples from nine patients. Values are log-counts per million, scaled within each gene. Annotations indicate donor MoCA group, state label, and gene-set direction; hierarchical clustering is applied to rows and columns. The 'IFN' and 'classical' state annotations refer here to clusters 5 and 0, respectively. This is an in-sample description of the derived signature rather than an independent validation.

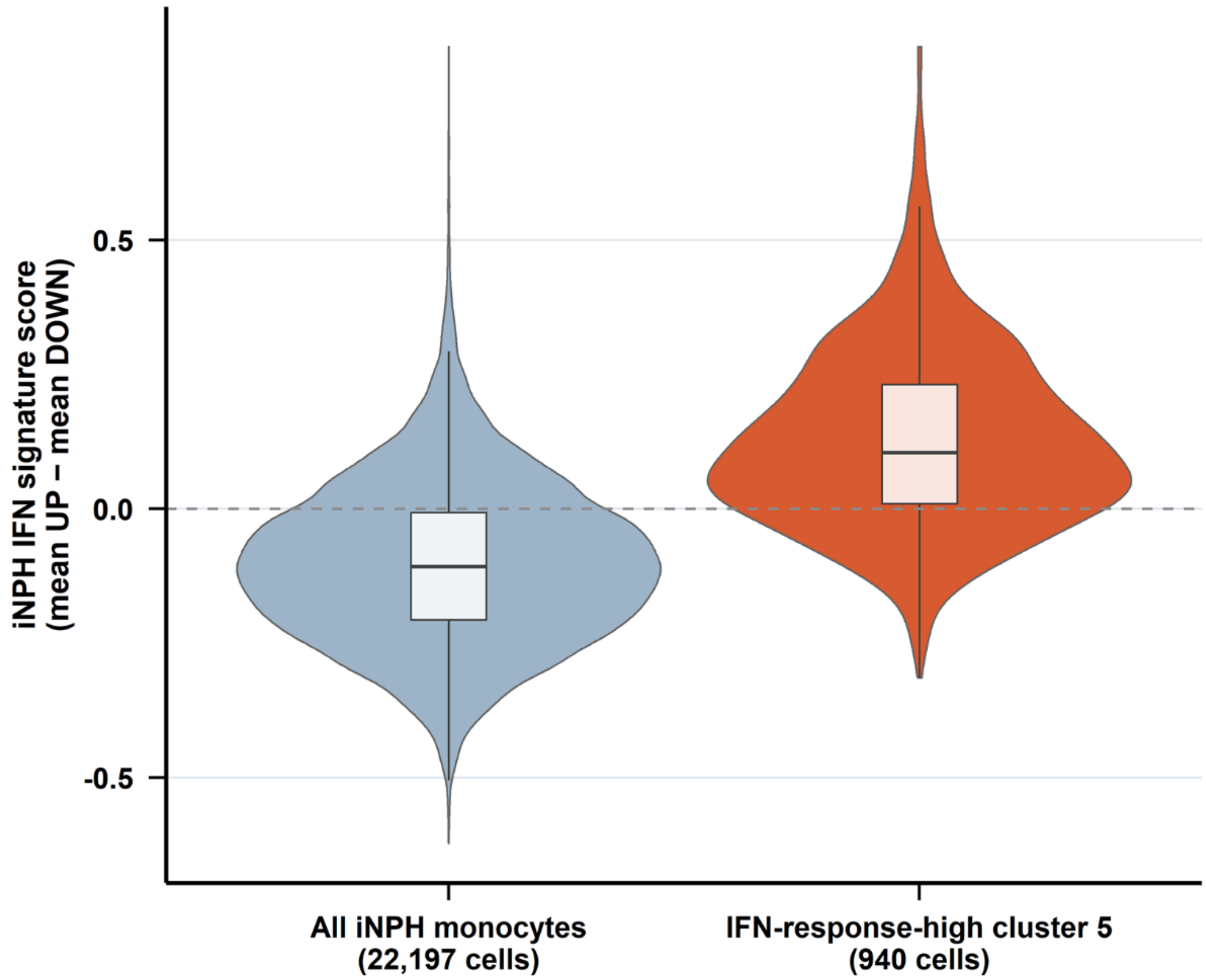

Supplementary Figure S4. Distribution of the signature score within canonical monocytes. The score is the mean log-normalized expression of the 150 positive signature genes minus that of the 150 negative genes. Distributions are shown for all 22,197 canonical monocytes and for the 940 cells in cluster 5, with means of −0.101 and +0.129, respectively. The all-monocyte population includes cluster 5; the displayed groups are therefore not independent. Violins show score distributions, boxes indicate medians and interquartile ranges, and the dashed line marks zero. Because cluster 5 contributed to signature derivation, the separation is descriptive and does not independently validate the state or demonstrate a requirement for discrete clustering.

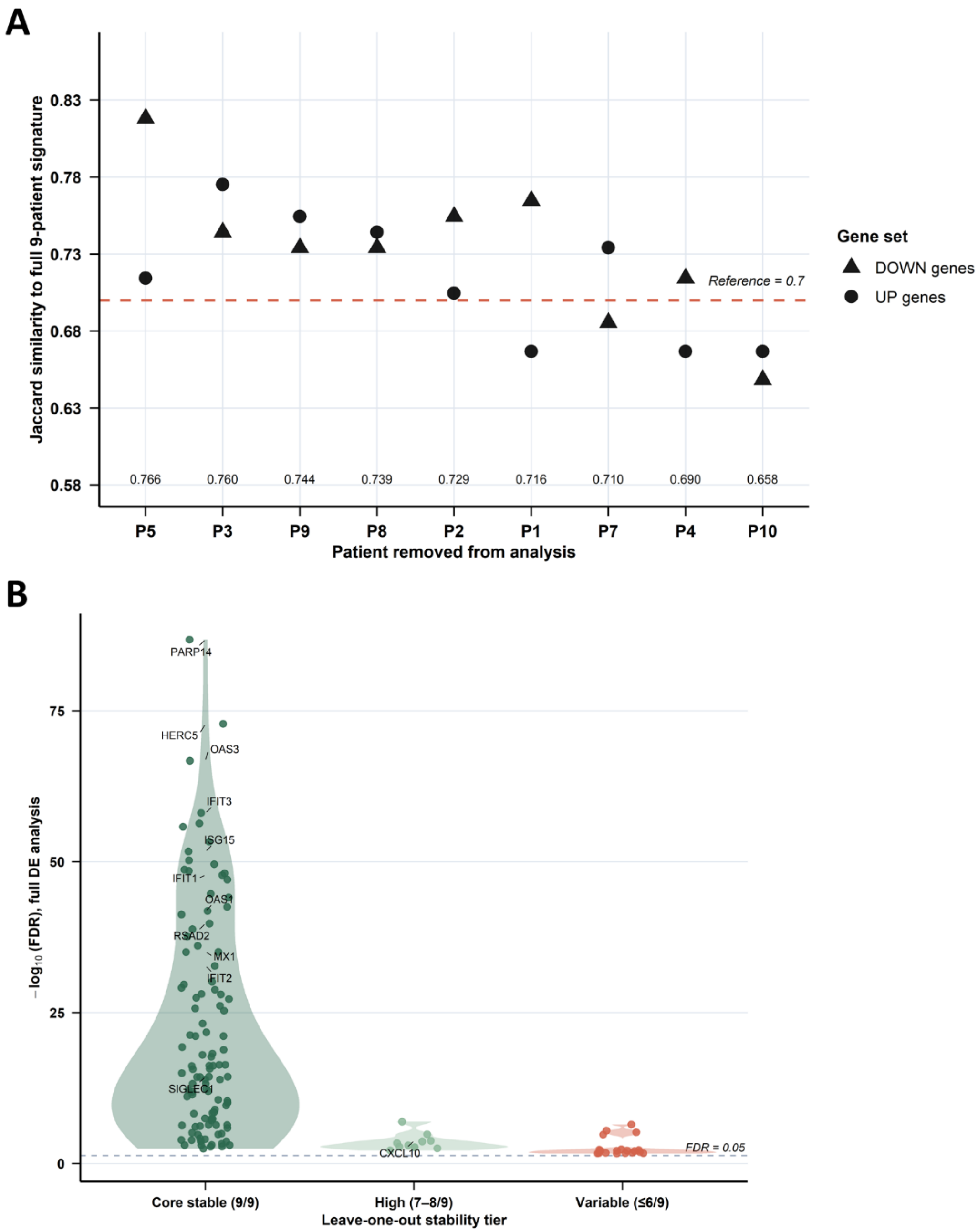

Supplementary Figure S5. Conditional donor-omission stability of the gene signature. (A) Jaccard similarity between each donor-omission signature and the full nine-donor signature, calculated separately for positive genes (circles) and negative genes (triangles). Cluster-5 and cluster-0 labels were retained while the paired pseudobulk model was refit. The dashed line at 0.70 is an operational reference; mean positive/negative Jaccard similarity for each omission is shown below the axis. Point colors do not encode MoCA groups. (B) Persistence of the original 150 positive signature genes across the nine donor omissions. In total, 137 genes appeared in at least one iteration-specific positive set and 107 appeared in all nine. The panel assesses persistence conditional on the original labels and does not evaluate complete pipeline reconstruction or persistence of the original negative genes.

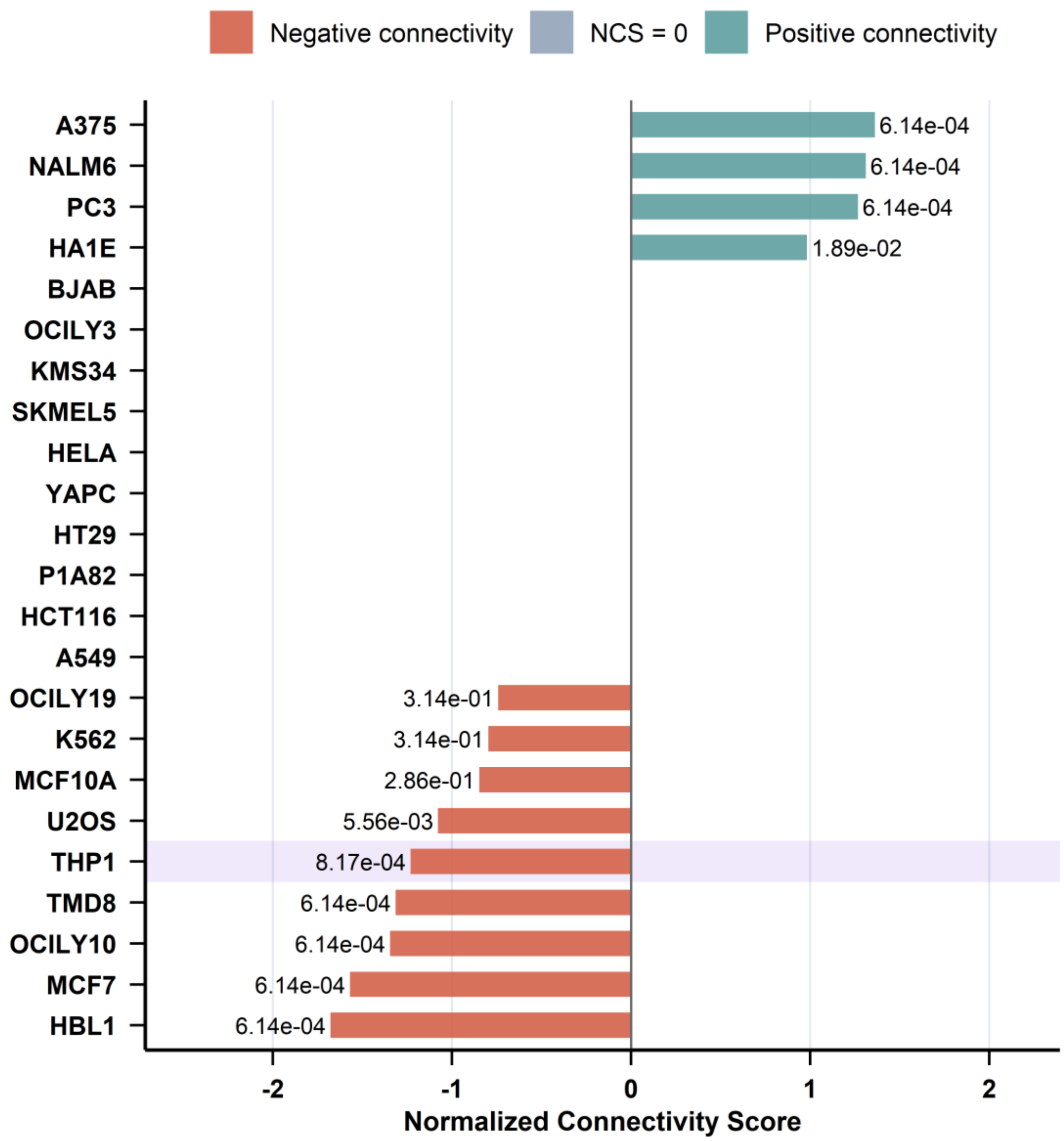

Supplementary Figure S6. Cell-line connectivity of the post hoc secondary candidate baricitinib. PRIMARY_150 NCS values across the cell lines represented in the executed LINCS2 results. Negative and positive scores indicate inverse and positive transcriptional connectivity; exact-zero scores are not interpreted as biological neutrality. Labels show saved WTCS FDR values for every nonzero score, including values ≥0.05. THP1, the transformed monocytic screening context, is highlighted. Baricitinib ranked 39th in the primary THP1 screen (NCS approximately −1.23; FDR = 8.17×10^{-4}) and was examined after the original top-30 review because of its JAK1/JAK2 pharmacology. Connectivity varies across cell contexts and does not establish patient-cell efficacy or JAK-inhibitor class enrichment.

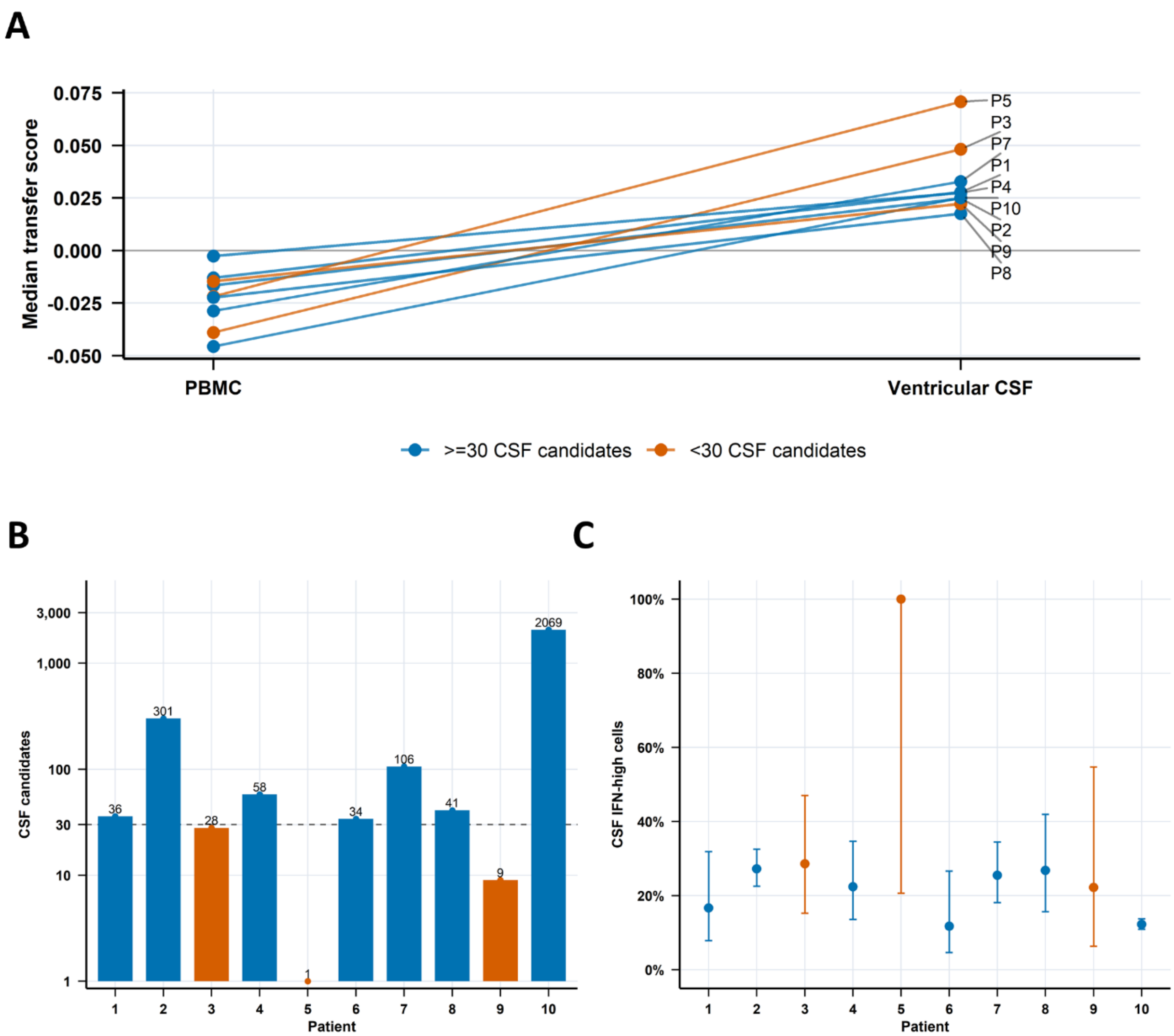

Supplementary Figure S7. Supportive ventricular-CSF contextualization of the blood-derived IFN-response-high program. (A) Paired median transfer scores in PBMC and ventricular-CSF candidate monocytes. The transfer score is AUCell enrichment of the selector-excluded positive blood signature minus enrichment of the original negative signature. CSF candidates were selected without IFN markers. Lines connect samples from the same donor; colors distinguish donors with at least 30 versus fewer than 30 recovered CSF candidate cells. Among the six pairs meeting the ≥30-cell criterion, all CSF-minus-PBMC differences were positive (median difference = 0.0410; range, 0.0301–0.0707; Wilcoxon V = 21; nominal unadjusted P = 0.036). All nine pairs also showed positive differences (median difference = 0.0413; V = 45; nominal unadjusted P = 0.009). Both two-sided Wilcoxon signed-rank tests used a normal approximation with continuity correction. These comparisons provide exploratory context for detectability of the transferred program in selected CSF cells, not evidence of compartmental enrichment. (B) Number of recovered ventricular-CSF candidate monocytes per donor, displayed on a logarithmic scale. The dashed horizontal line marks 30 cells. Recovery ranged from 1 to 2,069 cells; Patient 10 contributed 77.12% of all selected CSF candidates, corresponding to an effective donor number of 1.64 among ten donors. (C) Descriptive donor-level percentages of recovered CSF candidates exceeding the frozen IFN-response-high threshold. Points show donor estimates; error bars show 95% Wilson intervals conditional on recovered-cell counts.

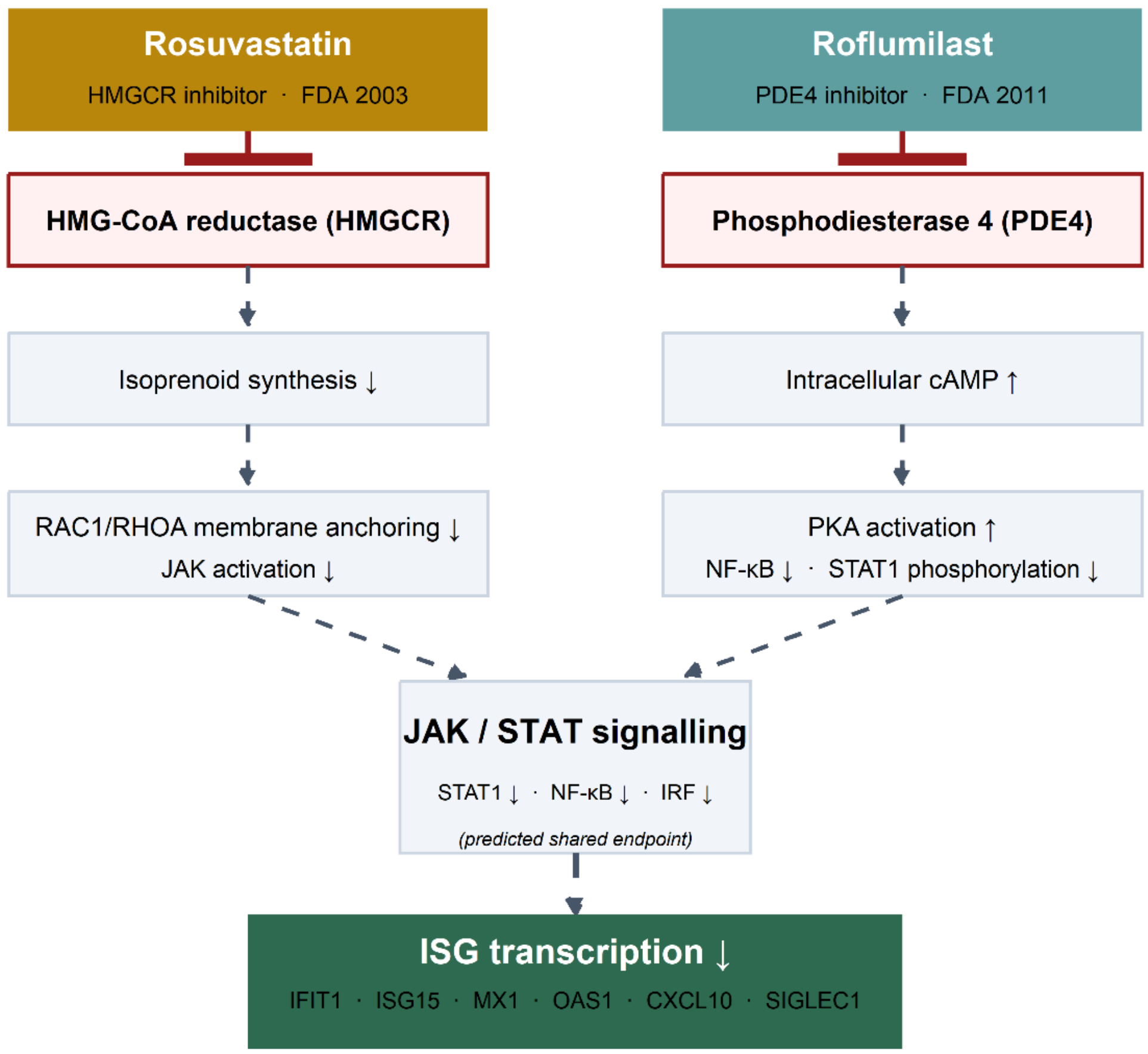

= enzyme inhibition · Arrows (→) = hypothesized downstream activation · ↑/↓ = relative change ·

Supplementary Figure S8. Hypothesis-generating mechanisms through which rosuvastatin and roflumilast could oppose IFN-associated transcription. The schematic summarizes literature-supported pharmacological targets and proposed downstream pathways that could converge on reduced JAK/STAT-, NF-κB- and IRF-associated signaling and lower interferon-stimulated gene transcription. Rosuvastatin inhibits HMG-CoA reductase (HMGCR), potentially reducing isoprenoid synthesis, RAC1/RHOA membrane anchoring and downstream inflammatory signaling. Roflumilast inhibits phosphodiesterase 4 (PDE4), increasing intracellular cAMP and protein kinase A activity, which may attenuate NF-κB activity and STAT1 phosphorylation. Blunt-ended bars denote established enzyme inhibition, dashed arrows denote hypothesized downstream relationships, and upward or downward arrows denote the proposed direction of change. The depicted downstream mechanisms were not experimentally tested in iNPH and should not be interpreted as evidence of therapeutic efficacy. U.S. regulatory approval refers to established non-iNPH indications.

Supplementary Tables

Supplementary Table S1. Publicly available donor characteristics and analysis-specific cell counts.

Source-reported MoCA groups and recovered cell counts are shown where they can be linked to donor identifiers. Exact age, sex, medication exposure, and MoCA values are marked unavailable when no reliable patient-level linkage was present in the reviewed public records. Patient 6 is retained in the uniform ten-donor sensitivity reconstruction and descriptive CSF analysis; unavailable MoCA information was not imputed. Canonical, reconstructed, and CSF counts refer to distinct documented analysis populations.

Supplementary Table S1A. Donor identifiers and publicly available characteristics.

Patient	CSF GSM	PBMC GSM	MoCA group	Exact MoCA	Public group-level MoCA values	Age, y	Sex	Relevant medications
1	GSM8851048	GSM8851049	High	NR	23; 23; 25; 27; 29 (group-level values; patient mapping unavailable)	NR	NR	NR
2	GSM8851050	GSM8851051	High	NR	23; 23; 25; 27; 29 (group-level values; patient mapping unavailable)	NR	NR	NR
3	GSM8851052	GSM8851053	High	NR	23; 23; 25; 27; 29 (group-level values; patient mapping unavailable)	NR	NR	NR
4	GSM8851054	GSM8851055	High	NR	23; 23; 25; 27; 29 (group-level values; patient mapping unavailable)	NR	NR	NR
5	GSM8851056	GSM8851057	High	NR	23; 23; 25; 27; 29 (group-level values; patient mapping unavailable)	NR	NR	NR
6	GSM8851058	GSM8851059	Unknown	NR	Not reported	NR	NR	NR
7	GSM8851060	GSM8851061	Low	NR	19; 20; 20; 20 (group-level values; patient mapping unavailable)	NR	NR	NR
8	GSM8851062	GSM8851063	Low	NR	19; 20; 20; 20 (group-level values; patient mapping unavailable)	NR	NR	NR
9	GSM8851064	GSM8851065	Low	NR	19; 20; 20; 20 (group-level values; patient mapping unavailable)	NR	NR	NR
10	GSM8851066	GSM8851067	Low	NR	19; 20; 20; 20 (group-level values; patient mapping unavailable)	NR	NR	NR

Supplementary Table S1B. Availability of patient-level clinical fields.

Patient	Age availability	Sex availability	Medication availability	Exact MoCA availability
1	Not reported at donor level	Not reported at donor level	Not reported at donor level	Exact values reported only as an unordered group-level set
2	Not reported at donor level	Not reported at donor level	Not reported at donor level	Exact values reported only as an unordered group-level set
3	Not reported at donor level	Not reported at donor level	Not reported at donor level	Exact values reported only as an unordered group-level set
4	Not reported at donor level	Not reported at donor level	Not reported at donor level	Exact values reported only as an unordered group-level set
5	Not reported at donor level	Not reported at donor level	Not reported at donor level	Exact values reported only as an unordered group-level set
6	Not reported at donor level	Not reported at donor level	Not reported at donor level	Neither exact score nor group publicly reported
7	Not reported at donor level	Not reported at donor level	Not reported at donor level	Exact values reported only as an unordered group-level set
8	Not reported at donor level	Not reported at donor level	Not reported at donor level	Exact values reported only as an unordered group-level set
9	Not reported at donor level	Not reported at donor level	Not reported at donor level	Exact values reported only as an unordered group-level set
10	Not reported at donor level	Not reported at donor level	Not reported at donor level	Exact values reported only as an unordered group-level set

Supplementary Table S1C. PBMC cell recovery and analysis populations.

Patient	Raw PBMC loaded	Post-QC PBMC	Uniform PBMC candidates	Canonical PBMC monocytes	Canonical marker-high	Canonical cluster 5	Primary nine-patient analysis
1	18,973	18,784	2,921	2,852	384	85	Yes
2	12,809	12,737	1,864	1,804	720	179	Yes
3	13,073	12,881	3,905	3,797	252	69	Yes
4	12,770	12,698	2,000	1,953	249	91	Yes
5	32,268	31,989	2,311	2,261	206	50	Yes
6	7,512	7,441	2,004	NR	NR	NR	No
7	9,028	8,864	1,442	1,416	337	120	Yes
8	7,181	7,137	2,282	2,230	401	132	Yes
9	7,441	7,353	1,367	1,343	397	116	Yes
10	10,504	10,281	4,607	4,541	249	98	Yes

Supplementary Table S1D. Ventricular-CSF candidate-cell summaries.

Patient	CSF candidates	CSF marker-high	CSF marker-high, %	Median transfer score	At least 30 CSF candidates
1	36	6	16.7	0.028	Yes
2	301	82	27.2	0.025	Yes
3	28	8	28.6	0.048	No
4	58	13	22.4	0.027	Yes
5	1	1	100	0.071	No
6	34	4	11.8	0.006	Yes
7	106	27	25.5	0.033	Yes
8	41	11	26.8	0.018	Yes
9	9	2	22.2	0.022	No
10	2069	254	12.3	0.025	Yes

NR, not reported or not publicly linkable at the patient level; GSM, Gene Expression Omnibus sample identifier; PBMC, peripheral blood mononuclear cell; CSF, cerebrospinal fluid.

Supplementary Table S2. FDR-significant genes in the within-iNPH cluster-5-versus-cluster-0 contrast.

Paired pseudobulk differential expression used DESeq2 design ~ patient + state with apeglm shrinkage, comparing cluster 5 (940 cells) with cluster 0 (4,904 cells) across nine donors. The table lists the 1,231 genes with FDR < 0.05, ordered by shrunken log2 fold change. The query selected the 150 largest positive and 150 most negative significant genes. baseMean, mean normalized count; lfcSE, reported log2 fold-change uncertainty; padj, Benjamini–Hochberg-adjusted P value. This table is restricted to significant genes and is not the complete set of tested genes.

Gene	baseMean	log2FC	lfcSE	p-value	padj
CXCL10	20.98	7.621	2.871	7.126e-06	1.690e-04
IFIT1	45.21	5.438	0.364	2.656e-51	1.589e-48
IFI44L	81.88	4.548	0.600	4.010e-19	4.451e-17
USP18	4.41	4.369	0.819	2.652e-09	1.190e-07
IFIT2	61.19	4.056	0.338	7.177e-36	1.840e-33
IFIT3	63.24	4.036	0.246	3.747e-62	8.070e-59
CMPK2	37.99	3.908	0.261	1.285e-51	8.142e-49
SIGLEC1	56.33	3.833	0.522	4.731e-17	4.245e-15
RSAD2	29.06	3.825	0.281	5.089e-43	1.712e-40
AP001610.2	3.79	3.781	0.725	7.770e-09	3.333e-07
RNASE1	3.41	3.662	0.901	5.412e-07	1.619e-05
OAS3	69.49	3.580	0.204	6.839e-71	1.841e-67
HERC5	64.16	3.573	0.194	2.658e-77	1.431e-73
RGL1	13.96	3.522	0.374	3.711e-22	5.124e-20
ISG15	201.60	3.482	0.229	1.964e-55	1.923e-52
MX1	196.61	3.465	0.281	3.271e-38	9.031e-36
ATP10A	17.34	3.077	0.312	3.466e-24	5.256e-22
IFI6	119.63	3.064	0.290	1.107e-28	1.987e-26
CCL2	7.77	3.059	0.673	1.017e-07	3.543e-06
LY6E	186.89	3.055	0.355	4.924e-21	6.094e-19
OAS2	64.41	2.995	0.185	2.543e-60	4.564e-57
OASL	35.01	2.918	0.227	2.975e-39	8.658e-37
IFI44	90.89	2.883	0.262	1.650e-30	3.352e-28
EPSTI1	107.35	2.836	0.251	7.373e-32	1.620e-29
ETV7	3.99	2.812	0.618	2.000e-07	6.488e-06
GMPR	8.84	2.768	0.459	1.892e-11	1.061e-09
GBP1	38.37	2.732	0.215	3.024e-38	8.570e-36
SERPING1	12.50	2.696	0.336	5.165e-17	4.559e-15
FPR3	2.52	2.599	1.022	3.203e-04	0.0047
IFITM1	46.07	2.594	0.222	3.090e-33	7.394e-31
EIF2AK2	88.04	2.487	0.158	3.892e-57	4.550e-54
SPATS2L	20.14	2.451	0.292	2.019e-18	2.051e-16
SORBS3	6.11	2.345	0.529	3.554e-07	1.100e-05
HESX1	2.38	2.247	0.809	1.824e-04	0.0029
SAMD9L	59.10	2.245	0.147	7.205e-54	5.968e-51
HERC6	15.69	2.220	0.276	5.160e-17	4.559e-15
AC004551.1	3.05	2.200	0.716	7.691e-05	0.0014
HLA-DQA1	28.58	2.106	0.276	6.898e-16	5.758e-14
XAF1	145.43	2.076	0.141	1.684e-50	9.065e-48
DDX60	24.26	2.029	0.224	8.145e-21	9.854e-19
LAP3	100.82	2.011	0.134	2.866e-52	2.057e-49
JUP	18.21	2.010	0.365	6.775e-10	3.228e-08
ABTB2	7.19	1.931	0.424	2.496e-07	7.929e-06
PARP14	175.32	1.890	0.094	1.468e-91	1.580e-87
NEXN	16.78	1.883	0.259	1.440e-14	1.041e-12
GBP4	24.69	1.857	0.236	1.416e-16	1.219e-14
MSR1	10.38	1.841	0.351	9.138e-09	3.859e-07
DDX58	45.85	1.835	0.157	9.246e-33	2.164e-30
ITGB5	2.41	1.791	0.798	8.773e-04	0.0109
IFIT5	12.12	1.779	0.315	8.583e-10	4.018e-08
OAS1	94.35	1.754	0.126	4.030e-45	1.446e-42
PNPT1	23.91	1.733	0.211	1.077e-17	1.035e-15
IRF7	98.32	1.714	0.119	4.571e-48	2.051e-45
DHX58	14.94	1.683	0.275	6.100e-11	3.173e-09
KLF12	6.39	1.665	0.527	4.760e-05	9.047e-04
PPM1K	14.35	1.625	0.315	1.135e-08	4.699e-07
HELZ2	31.66	1.615	0.193	2.363e-18	2.356e-16
PARP9	62.43	1.608	0.122	8.205e-41	2.524e-38
HEG1	5.54	1.595	0.533	9.803e-05	0.0017
CCR5AS	3.75	1.572	0.597	3.447e-04	0.0050
MX2	152.47	1.567	0.105	4.986e-52	3.356e-49
AC116407.2	2.71	1.564	0.723	5.828e-04	0.0078
SASH1	29.51	1.551	0.213	1.730e-14	1.242e-12
IFI35	63.65	1.529	0.134	3.758e-31	7.934e-29
KLHDC7B	5.80	1.517	0.434	1.906e-05	4.025e-04
SAMD9	34.31	1.516	0.178	6.629e-19	6.998e-17
DTNA	3.62	1.501	0.600	4.132e-04	0.0058
IFITM3	331.74	1.482	0.187	4.531e-17	4.100e-15
EPHB2	12.26	1.473	0.283	1.010e-08	4.217e-07
SAMD4A	22.83	1.465	0.244	8.239e-11	4.225e-09

Gene	baseMean	log2FC	lfcSE	p-value	padj
ISG20	24.94	1.455	0.244	1.076e-10	5.491e-09
NRIR	3.33	1.448	0.628	5.676e-04	0.0076
IFIH1	35.05	1.441	0.168	5.906e-19	6.297e-17
APOL6	58.05	1.435	0.126	4.829e-31	1.000e-28
AGRN	3.65	1.434	0.592	5.076e-04	0.0069
UBE2L6	104.26	1.428	0.100	2.065e-47	8.552e-45
LMNA	35.21	1.428	0.294	3.353e-08	1.256e-06
STAT1	176.92	1.416	0.106	4.780e-42	1.514e-39
ITGB7	4.92	1.416	0.474	1.134e-04	0.0019
SEPTIN5	3.22	1.401	0.732	0.0014	0.0161
GBP5	18.41	1.394	0.252	1.508e-09	6.881e-08
RNF144A	2.96	1.373	0.733	0.0017	0.0190
APOBEC3A	181.98	1.367	0.123	2.870e-30	5.617e-28
EXOC3L1	7.13	1.333	0.405	4.628e-05	8.883e-04
CALHM2	15.67	1.317	0.252	9.762e-09	4.090e-07
SLFN5	21.38	1.312	0.231	8.295e-10	3.900e-08
ZCCHC2	37.98	1.300	0.169	7.082e-16	5.866e-14
PTK2	11.76	1.288	0.331	4.771e-06	1.181e-04
FCGR3A	11.44	1.282	0.291	5.481e-07	1.635e-05
CHROMR	9.78	1.280	0.347	9.096e-06	2.102e-04
DTX3L	41.75	1.272	0.149	5.799e-19	6.244e-17
MT1E	7.59	1.264	0.403	8.112e-05	0.0014
CLEC10A	7.86	1.202	0.364	4.600e-05	8.853e-04
FANCL	7.43	1.192	0.430	2.240e-04	0.0035
SEZ6L	4.63	1.176	0.612	0.0014	0.0161
PPA1	31.18	1.169	0.171	4.512e-13	2.825e-11
CTSL	7.03	1.169	0.478	4.791e-04	0.0066
PML	42.46	1.161	0.157	7.927e-15	6.130e-13
HLA-DPA1	310.56	1.151	0.082	7.922e-46	3.047e-43
SDSL	3.99	1.151	0.610	0.0019	0.0207
TRIM22	126.24	1.148	0.117	5.133e-24	7.572e-22
AC006160.1	2.98	1.136	0.780	0.0032	0.0316
ATIC	9.20	1.135	0.326	2.508e-05	5.145e-04
EPS8	8.87	1.134	0.395	1.822e-04	0.0029
HLA-DPB1	281.49	1.128	0.082	1.569e-44	5.451e-42
ZNF496	8.15	1.118	0.361	8.753e-05	0.0015
TMEM123	108.32	1.118	0.096	3.358e-32	7.693e-30
DDX60L	100.15	1.116	0.108	3.755e-26	6.221e-24
RNF213	436.78	1.105	0.073	3.404e-53	2.618e-50
PARP12	55.44	1.103	0.162	6.811e-13	4.167e-11
MS4A4A	12.85	1.099	0.276	3.742e-06	9.548e-05
SNHG32	21.10	1.098	0.216	2.041e-08	8.080e-07
CNP	14.22	1.097	0.270	2.423e-06	6.426e-05
NLE1	2.25	1.092	0.928	0.0050	0.0451
CDK18	2.56	1.091	1.038	0.0052	0.0468
U62317.4	10.26	1.075	0.322	4.448e-05	8.630e-04
PARP3	2.53	1.075	0.966	0.0043	0.0402
CDKN1A	28.08	1.074	0.210	1.490e-08	6.011e-07
AL031123.1	4.88	1.054	0.521	0.0016	0.0182
LGALS3BP	4.91	1.045	0.536	0.0017	0.0190
MT2A	295.49	1.045	0.107	5.049e-24	7.551e-22
BST2	159.30	1.044	0.097	2.757e-28	4.867e-26
TRPM4	3.79	1.037	0.641	0.0030	0.0297
PLAC8	81.55	1.032	0.115	1.742e-20	2.084e-18
BLVRA	102.97	1.020	0.102	1.155e-24	1.802e-22
DNPH1	16.68	1.019	0.275	9.520e-06	2.167e-04
TRIM5	18.28	1.017	0.228	5.168e-07	1.550e-05
LIPA	38.76	1.008	0.154	3.824e-12	2.214e-10
HLA-DQA2	7.51	1.007	0.643	0.0021	0.0227
PLSCR1	114.13	1.000	0.108	1.046e-21	1.390e-19
NIT2	7.48	1.000	0.447	9.854e-04	0.0120
FABP5	8.18	0.997	0.552	0.0015	0.0170
TRIM14	45.10	0.993	0.149	1.457e-12	8.665e-11
CXXC5	12.30	0.992	0.376	2.895e-04	0.0043
FSCN1	3.02	0.990	0.738	0.0042	0.0392
SSB	55.55	0.986	0.132	5.605e-15	4.406e-13
STAT2	130.69	0.981	0.089	3.947e-29	7.328e-27
RNASE2	29.61	0.978	0.221	4.639e-07	1.399e-05
PI4K2B	16.63	0.975	0.251	5.185e-06	1.272e-04
HLCS	6.71	0.973	0.405	6.831e-04	0.0089
ZBP1	13.82	0.969	0.326	1.314e-04	0.0022
SH3PXD2B	11.46	0.961	0.393	5.315e-04	0.0072
MARCKS	237.18	0.956	0.121	1.336e-16	1.169e-14
GAS6	6.52	0.951	0.480	0.0017	0.0189
GAS5	53.23	0.950	0.133	5.337e-14	3.638e-12

Gene	baseMean	log2FC	lfcSE	p-value	padj
TLR7	16.08	0.944	0.251	9.444e-06	2.164e-04
ID2	72.21	0.940	0.134	1.200e-13	7.930e-12
IFI16	127.66	0.936	0.109	3.773e-19	4.276e-17
HLA-DRA	710.78	0.933	0.058	1.082e-59	1.664e-56
CPED1	12.83	0.930	0.283	5.081e-05	9.548e-04
CD2AP	19.20	0.927	0.225	2.298e-06	6.186e-05
HLA-DRB5	270.38	0.926	0.097	7.965e-23	1.129e-20
RTP4	7.06	0.918	0.494	0.0019	0.0210
TAP1	45.83	0.909	0.146	3.171e-11	1.733e-09
LILRB4	35.58	0.908	0.157	4.832e-10	2.323e-08
CALCRL	6.36	0.901	0.460	0.0019	0.0208
F13A1	81.70	0.901	0.123	1.368e-14	9.950e-13
HNRNPA1	183.65	0.899	0.069	6.840e-40	2.046e-37
LGALS9	159.36	0.895	0.080	2.921e-30	5.617e-28
MOV10	11.69	0.890	0.345	4.477e-04	0.0062
ABI3	28.03	0.887	0.219	2.775e-06	7.306e-05
COA1	36.00	0.878	0.167	9.344e-09	3.930e-07
RTN4IP1	3.99	0.875	0.659	0.0044	0.0408
TOR1B	16.02	0.870	0.315	2.530e-04	0.0038
TSPAN3	8.94	0.861	0.373	8.914e-04	0.0110
SP110	130.87	0.853	0.091	4.311e-22	5.803e-20
TAP2	31.61	0.850	0.176	9.999e-08	3.498e-06
HLA-DRB1	582.35	0.846	0.073	4.878e-32	1.094e-29
NFIX	9.43	0.840	0.340	6.621e-04	0.0087
CUL1	56.57	0.839	0.158	6.088e-09	2.633e-07
LDLR	13.21	0.839	0.304	3.164e-04	0.0047
EPB41L3	49.43	0.838	0.144	3.570e-10	1.747e-08
KIAA1958	30.80	0.835	0.177	1.616e-07	5.386e-06
DIPK1A	6.11	0.827	0.532	0.0037	0.0357
ZC3HAV1	75.10	0.826	0.115	4.740e-14	3.272e-12
ATF5	26.84	0.826	0.211	5.400e-06	1.318e-04
CD40	8.30	0.825	0.375	0.0013	0.0156
TNNT1	12.57	0.824	0.367	0.0010	0.0123
RPL3	667.16	0.823	0.052	2.714e-57	3.653e-54
PRLR	29.30	0.823	0.202	2.803e-06	7.360e-05
PID1	163.24	0.819	0.148	1.498e-09	6.863e-08
ITGA4	133.87	0.816	0.097	2.499e-18	2.469e-16
CMTR1	23.76	0.815	0.239	3.777e-05	7.477e-04
MARCO	21.32	0.814	0.247	5.266e-05	9.844e-04
EEF2	423.16	0.811	0.051	4.225e-57	4.550e-54
NME1	10.09	0.809	0.324	6.288e-04	0.0084
PSME2	186.64	0.807	0.098	1.948e-17	1.808e-15
ARL4C	5.16	0.805	0.657	0.0050	0.0453
TENT5A	60.11	0.797	0.126	2.110e-11	1.177e-09
CLECL1	3.62	0.793	1.129	0.0055	0.0488
CAMK2D	80.74	0.792	0.122	7.995e-12	4.531e-10
SPATA20	7.41	0.791	0.382	0.0018	0.0198
CHCHD4	6.30	0.790	0.468	0.0036	0.0346
CALR	63.32	0.790	0.126	2.481e-11	1.377e-09
ADAR	156.02	0.790	0.080	2.695e-24	4.146e-22
PEBP1	19.93	0.786	0.223	2.886e-05	5.874e-04
SHFL	38.12	0.786	0.170	2.430e-07	7.765e-06
ABCE1	11.73	0.784	0.285	3.502e-04	0.0051
HSPA8	144.31	0.783	0.085	2.795e-21	3.626e-19
PHF11	56.40	0.780	0.125	3.388e-11	1.833e-09
SLC18B1	12.67	0.774	0.279	3.173e-04	0.0047
TNFSF10	150.81	0.766	0.109	1.417e-13	9.247e-12
SCO2	78.99	0.763	0.151	2.916e-08	1.109e-06
NPM1	111.21	0.762	0.090	1.438e-18	1.489e-16
TRIM34	18.90	0.755	0.222	4.387e-05	8.558e-04
RHOC	16.69	0.754	0.278	3.944e-04	0.0056
AFF3	12.31	0.752	0.313	9.146e-04	0.0112
CCR2	38.84	0.752	0.157	1.310e-07	4.449e-06
HLA-DMA	151.41	0.750	0.091	2.300e-17	2.117e-15
SPINT2	37.46	0.748	0.183	2.878e-06	7.540e-05
RPL10A	461.07	0.742	0.070	3.725e-27	6.268e-25
WARS2	11.13	0.740	0.387	0.0026	0.0265
MTMR11	18.13	0.739	0.245	1.544e-04	0.0025
RAPH1	7.62	0.738	0.513	0.0050	0.0453
RPS18	1481.56	0.737	0.048	5.587e-55	5.014e-52
LGALS2	276.89	0.735	0.077	8.548e-23	1.195e-20
RPS6	916.42	0.733	0.051	9.748e-48	4.199e-45
HSPE1	30.77	0.732	0.180	3.782e-06	9.628e-05
TUBB1	38.24	0.732	0.239	1.077e-04	0.0018
CBR1	30.97	0.729	0.220	5.355e-05	9.975e-04
HLA-DQB1	161.99	0.725	0.111	6.098e-12	3.493e-10
BISPR	11.25	0.718	0.295	8.580e-04	0.0107

Gene	baseMean	log2FC	lfcSE	p-value	padj
RNF5	16.71	0.716	0.242	2.121e-04	0.0033
TUBA1B	89.00	0.715	0.103	3.635e-13	2.316e-11
PRDX2	24.97	0.714	0.214	6.080e-05	0.0011
SP140	9.69	0.711	0.349	0.0020	0.0219
SNHG29	150.69	0.708	0.092	1.189e-15	9.698e-14
EEF1A1	3152.76	0.704	0.039	2.326e-74	8.349e-71
SPRED1	8.64	0.699	0.386	0.0034	0.0332
NHSL1	25.75	0.698	0.199	3.496e-05	6.972e-04
IDH2	36.11	0.696	0.161	1.291e-06	3.669e-05
WARS	142.72	0.696	0.101	5.161e-13	3.194e-11
SLC1A3	29.91	0.695	0.254	4.330e-04	0.0060
ATP5F1B	127.45	0.694	0.096	3.491e-14	2.425e-12
IL1RN	33.69	0.691	0.185	1.350e-05	2.974e-04
RPL4	478.36	0.689	0.048	1.058e-48	4.953e-46
ZNF124	18.73	0.689	0.233	2.213e-04	0.0035
NDUFA8	19.75	0.689	0.219	1.170e-04	0.0020
ITSN1	40.25	0.689	0.204	4.764e-05	9.047e-04
ICAM2	24.37	0.687	0.220	1.229e-04	0.0020
ZNF782	6.91	0.683	0.431	0.0047	0.0429
CD74	2233.73	0.675	0.047	2.806e-47	1.119e-44
PSMB9	179.32	0.675	0.078	3.842e-19	4.310e-17
TYMP	738.34	0.669	0.078	5.696e-19	6.196e-17
RPL15	893.47	0.663	0.045	6.262e-51	3.549e-48
RAB13	10.93	0.663	0.364	0.0033	0.0321
DRAP1	127.01	0.663	0.084	2.312e-16	1.976e-14
BTN3A3	13.12	0.661	0.320	0.0021	0.0227
PNO1	9.05	0.660	0.387	0.0042	0.0395
MTSS1	96.83	0.656	0.149	8.032e-07	2.363e-05
AHCY	13.56	0.655	0.289	0.0015	0.0171
SRSF8	11.04	0.654	0.315	0.0023	0.0243
CCR1	56.05	0.653	0.139	2.288e-07	7.354e-06
CCDC50	17.24	0.652	0.234	3.750e-04	0.0054
RTCB	36.35	0.648	0.169	9.831e-06	2.229e-04
TCF4	25.67	0.645	0.187	4.436e-05	8.623e-04
IFT74	7.69	0.644	0.410	0.0052	0.0466
DDOST	40.15	0.640	0.152	2.201e-06	6.016e-05
EEF1B2	292.05	0.640	0.070	6.521e-21	7.980e-19
PATL1	27.56	0.638	0.198	9.734e-05	0.0017
NAPA	112.18	0.631	0.096	4.406e-12	2.537e-10
GP9	11.63	0.627	0.326	0.0031	0.0309
MYOF	37.52	0.627	0.158	6.453e-06	1.548e-04
CACNA2D4	19.90	0.627	0.241	6.634e-04	0.0087
BLMH	8.17	0.626	0.393	0.0054	0.0478
TGFBI	112.06	0.625	0.091	5.482e-13	3.373e-11
LINC02723	11.37	0.622	0.303	0.0026	0.0266
LDHB	44.35	0.621	0.152	4.294e-06	1.078e-04
TNFSF13B	227.83	0.619	0.067	4.448e-21	5.635e-19
AKR1B1	22.09	0.619	0.212	2.927e-04	0.0043
CREBL2	17.42	0.618	0.241	7.558e-04	0.0096
RPL36A	574.73	0.617	0.050	4.120e-36	1.082e-33
IRF9	71.18	0.610	0.136	7.502e-07	2.219e-05
PDIA6	37.82	0.608	0.154	7.647e-06	1.806e-04
SLC25A6	395.10	0.607	0.057	1.283e-27	2.192e-25
APEX1	32.25	0.602	0.199	1.889e-04	0.0030
ENG	16.64	0.602	0.295	0.0027	0.0276
LPAR6	24.32	0.601	0.241	9.537e-04	0.0116
MAFB	217.66	0.601	0.136	8.896e-07	2.589e-05
RPLP0	832.68	0.599	0.060	8.257e-25	1.308e-22
RPS2	1315.76	0.598	0.041	1.014e-48	4.953e-46
PABPC4	38.15	0.598	0.148	5.070e-06	1.247e-04
FBXO6	18.13	0.598	0.243	0.0010	0.0123
PPIA	374.43	0.596	0.054	6.572e-29	1.199e-26
TBC1D12	22.63	0.596	0.233	8.284e-04	0.0104
SMIM30	9.18	0.595	0.347	0.0051	0.0454
DDIT4	30.11	0.587	0.223	7.058e-04	0.0091
PSMA3	36.21	0.587	0.172	6.168e-05	0.0011
CD300C	24.20	0.586	0.219	6.032e-04	0.0081
TRIM25	69.79	0.583	0.120	1.001e-07	3.498e-06
NMI	45.79	0.582	0.148	8.975e-06	2.078e-04
SLC25A5	143.25	0.582	0.086	1.409e-12	8.432e-11
SNHG5	85.18	0.579	0.121	1.776e-07	5.813e-06
RPL37A	821.60	0.578	0.049	1.557e-33	3.810e-31
MRPL40	17.01	0.577	0.231	0.0010	0.0124
APOL1	10.09	0.576	0.333	0.0051	0.0460
EIF3F	165.88	0.575	0.080	8.902e-14	5.991e-12

Gene	baseMean	log2FC	lfcSE	p-value	padj
RPL27A	308.64	0.574	0.067	1.430e-18	1.489e-16
HSH2D	57.42	0.574	0.145	7.560e-06	1.789e-04
HSP90AB1	119.52	0.572	0.098	6.231e-10	2.982e-08
IMPDH2	14.63	0.571	0.315	0.0040	0.0379
STAC3	25.53	0.569	0.197	3.407e-04	0.0050
PSMB8	114.93	0.565	0.091	6.914e-11	3.579e-09
TRAPPC6A	20.35	0.561	0.213	7.508e-04	0.0096
PSMA5	52.28	0.554	0.130	1.974e-06	5.449e-05
RPL35	570.41	0.552	0.049	2.038e-30	4.063e-28
VCAN-AS1	17.96	0.551	0.249	0.0021	0.0224
ARF6	83.21	0.550	0.113	1.375e-07	4.656e-06
RPL23A	639.79	0.550	0.044	2.879e-36	7.749e-34
LINC02432	27.64	0.548	0.200	5.645e-04	0.0076
AL133415.1	48.84	0.548	0.144	1.626e-05	3.522e-04
JAK2	167.68	0.547	0.092	2.585e-10	1.283e-08
TUBB6	22.30	0.545	0.242	0.0021	0.0226
SLC35A4	16.46	0.538	0.244	0.0023	0.0243
MARVELD1	15.58	0.537	0.282	0.0042	0.0392
LYSMD2	35.15	0.537	0.186	3.689e-04	0.0053
NCOA7	27.85	0.536	0.188	4.252e-04	0.0060
APOBEC3G	13.05	0.534	0.284	0.0046	0.0422
GCH1	43.91	0.531	0.191	4.835e-04	0.0067
HLA-A	918.35	0.531	0.039	5.552e-43	1.812e-40
ERAP2	23.42	0.526	0.225	0.0017	0.0190
ST3GAL5	28.77	0.522	0.194	7.026e-04	0.0091
RPS27	1405.47	0.521	0.037	2.740e-45	1.017e-42
PEPD	40.55	0.520	0.158	1.252e-04	0.0021
AL627171.2	44.73	0.519	0.242	0.0024	0.0247
EIF5A	70.57	0.518	0.118	1.355e-06	3.820e-05
GCNT1	12.97	0.518	0.294	0.0056	0.0492
HLA-DMB	90.31	0.517	0.110	3.714e-07	1.140e-05
NRG1	109.00	0.515	0.124	3.598e-06	9.236e-05
CRELD2	13.95	0.515	0.283	0.0053	0.0473
NOP53	111.95	0.513	0.095	8.228e-09	3.516e-07
CYC1	46.69	0.512	0.142	3.722e-05	7.381e-04
TPT1	2491.34	0.511	0.035	1.282e-49	6.572e-47
GBP2	69.25	0.509	0.119	2.379e-06	6.349e-05
CAPN2	110.51	0.506	0.093	5.818e-09	2.526e-07
EPRS	28.73	0.505	0.190	7.892e-04	0.0100
RPS5	496.96	0.504	0.057	1.160e-19	1.373e-17
LDHA	72.90	0.502	0.121	4.489e-06	1.119e-04
C1QBP	35.44	0.501	0.163	2.341e-04	0.0036
RPS4X	999.58	0.500	0.046	3.042e-28	5.283e-26
CHMP5	34.16	0.500	0.166	2.996e-04	0.0044
VRK2	35.29	0.499	0.188	8.179e-04	0.0103
CCT6A	41.99	0.497	0.154	1.485e-04	0.0024
PARP10	43.45	0.497	0.149	1.115e-04	0.0019
BAG1	58.63	0.493	0.131	2.046e-05	4.285e-04
STOML2	21.25	0.492	0.205	0.0018	0.0198
CAMK1	34.25	0.491	0.182	7.318e-04	0.0094
CREG1	51.48	0.489	0.142	7.572e-05	0.0013
GPI	55.71	0.487	0.123	1.044e-05	2.342e-04
GNB4	51.78	0.486	0.138	4.997e-05	9.423e-04
RPS25	697.38	0.485	0.042	3.628e-31	7.814e-29
STOM	15.47	0.485	0.264	0.0056	0.0493
PYURF	50.57	0.485	0.130	2.372e-05	4.922e-04
RRAS	34.19	0.485	0.169	5.097e-04	0.0069
NME2	263.14	0.482	0.061	6.267e-16	5.272e-14
YIF1A	17.98	0.479	0.235	0.0041	0.0390
RPS29	473.13	0.478	0.049	1.373e-23	1.999e-21
TIAM1	74.44	0.478	0.138	6.723e-05	0.0012
GTF3C6	33.12	0.475	0.174	7.967e-04	0.0101
APOBEC3C	19.96	0.474	0.233	0.0042	0.0392
AL138963.4	70.11	0.473	0.146	1.454e-04	0.0024
CCT7	28.37	0.471	0.180	0.0010	0.0127
TMEM268	23.32	0.471	0.214	0.0028	0.0287
SNRPF	41.61	0.470	0.151	2.395e-04	0.0037
CNDP2	40.00	0.470	0.156	3.209e-04	0.0047
ZNRF2	40.10	0.469	0.152	2.549e-04	0.0039
LY86	110.33	0.468	0.112	4.174e-06	1.053e-04
SHISA5	68.27	0.465	0.118	1.015e-05	2.282e-04
UNC93B1	92.17	0.465	0.103	8.763e-07	2.557e-05
CD163	115.24	0.464	0.096	2.102e-07	6.796e-06
CBWD2	20.21	0.463	0.215	0.0035	0.0335
CASP4	79.07	0.460	0.111	4.782e-06	1.181e-04
TDRD7	19.19	0.459	0.233	0.0051	0.0455
CD14	392.11	0.458	0.066	4.284e-13	2.698e-11

Gene	baseMean	log2FC	lfcSE	p-value	padj
EIF3L	105.16	0.457	0.102	1.254e-06	3.573e-05
CD81	28.60	0.457	0.202	0.0027	0.0275
SPARC	31.04	0.455	0.215	0.0039	0.0369
MS4A4E	30.72	0.454	0.224	0.0044	0.0404
SLC25A39	26.73	0.450	0.188	0.0020	0.0214
RBM3	136.27	0.449	0.083	8.570e-09	3.633e-07
ADA2	196.54	0.449	0.072	6.058e-11	3.167e-09
SRM	28.32	0.446	0.182	0.0018	0.0198
ACTG1	715.77	0.446	0.053	3.223e-18	3.155e-16
EIF3D	70.66	0.445	0.118	2.441e-05	5.035e-04
RPL27	537.15	0.445	0.051	2.963e-19	3.394e-17
XRN1	71.71	0.445	0.117	2.253e-05	4.692e-04
RPSA	365.94	0.444	0.058	1.950e-15	1.544e-13
MDFIC	35.02	0.441	0.171	0.0013	0.0149
GIMAP4	123.42	0.441	0.090	1.673e-07	5.559e-06
PCGF5	44.91	0.441	0.160	8.026e-04	0.0101
ATP5F1A	88.62	0.440	0.101	2.060e-06	5.658e-05
GHITM	53.20	0.439	0.135	1.779e-04	0.0028
YBX1	305.55	0.439	0.063	4.571e-13	2.845e-11
RACK1	829.46	0.437	0.050	5.580e-19	6.131e-17
SRSF7	76.65	0.437	0.105	4.685e-06	1.165e-04
ODF3B	124.15	0.435	0.089	1.765e-07	5.807e-06
RPS16	794.25	0.434	0.042	6.913e-26	1.111e-23
EIF2A	43.54	0.434	0.180	0.0020	0.0213
EIF3E	139.75	0.434	0.082	1.939e-08	7.706e-07
EVL	26.45	0.431	0.187	0.0028	0.0285
HSP90AA1	184.09	0.429	0.077	5.640e-09	2.459e-07
MRPS7	24.92	0.429	0.197	0.0038	0.0362
DOCK10	67.74	0.428	0.117	4.063e-05	8.012e-04
DUS1L	32.28	0.428	0.176	0.0020	0.0218
PSMA4	61.71	0.427	0.131	1.714e-04	0.0028
ZFP36	340.62	0.425	0.083	5.425e-08	1.967e-06
PEA15	33.39	0.424	0.180	0.0025	0.0260
ATP8B4	44.55	0.424	0.154	8.861e-04	0.0110
EEF1G	426.61	0.421	0.059	1.199e-13	7.930e-12
SP100	157.03	0.418	0.082	6.022e-08	2.162e-06
NUB1	36.43	0.418	0.167	0.0018	0.0202
FBL	44.58	0.417	0.145	6.424e-04	0.0085
GPBAR1	43.43	0.417	0.154	0.0011	0.0127
ANKFY1	53.35	0.416	0.135	3.440e-04	0.0050
SFT2D2	45.36	0.415	0.144	6.193e-04	0.0083
VAMP5	130.73	0.413	0.090	7.647e-07	2.256e-05
EID1	119.56	0.413	0.100	6.074e-06	1.463e-04
UCP2	171.37	0.412	0.074	4.614e-09	2.028e-07
SAT1	1081.05	0.409	0.068	3.676e-10	1.783e-08
CASP1	125.56	0.408	0.082	1.064e-07	3.696e-06
SNHG6	101.03	0.408	0.109	2.917e-05	5.927e-04
MYD88	85.97	0.408	0.113	5.239e-05	9.812e-04
RNH1	133.41	0.405	0.087	6.208e-07	1.846e-05
HSP90B1	91.32	0.405	0.100	9.955e-06	2.252e-04
CRTAP	145.91	0.405	0.081	1.186e-07	4.079e-06
PSMB10	176.38	0.404	0.076	2.105e-08	8.195e-07
SDHD	38.24	0.403	0.157	0.0017	0.0190
PDK4	45.03	0.399	0.153	0.0015	0.0167
IMMP2L	37.43	0.398	0.154	0.0016	0.0182
ANXA2	363.09	0.396	0.062	3.250e-11	1.768e-09
RPL13	2756.57	0.396	0.038	5.798e-26	9.460e-24
PSMG2	45.40	0.395	0.160	0.0022	0.0232
SLC12A7	30.29	0.395	0.183	0.0047	0.0431
CSF1R	69.75	0.394	0.134	5.781e-04	0.0078
BANF1	61.27	0.393	0.122	2.271e-04	0.0035
APPL1	45.19	0.392	0.138	7.530e-04	0.0096
PRKAR2B	52.21	0.392	0.158	0.0022	0.0232
ARL4A	34.03	0.392	0.182	0.0049	0.0442
PSMA6	89.98	0.392	0.100	1.715e-05	3.663e-04
GRN	409.88	0.391	0.064	2.187e-10	1.095e-08
TLE5	85.77	0.391	0.111	9.093e-05	0.0016
NASP	28.08	0.391	0.184	0.0050	0.0451
BTF3	382.96	0.391	0.058	2.870e-12	1.671e-10
RPL38	414.79	0.390	0.053	2.615e-14	1.853e-12
RPL5	570.81	0.390	0.061	3.613e-11	1.935e-09
PPIB	157.69	0.390	0.076	5.134e-08	1.874e-06
SECTM1	85.66	0.390	0.115	1.340e-04	0.0022
C5orf56	38.49	0.390	0.154	0.0019	0.0211
IRF2	62.22	0.385	0.116	1.726e-04	0.0028

Gene	baseMean	log2FC	lfcSE	p-value	padj
CITED2	48.52	0.382	0.143	0.0013	0.0151
GADD45B	39.57	0.382	0.171	0.0041	0.0383
CD99	176.36	0.381	0.074	5.226e-08	1.901e-06
NPC2	287.61	0.381	0.057	6.278e-12	3.577e-10
AP1S2	300.74	0.380	0.063	3.038e-10	1.501e-08
PLD3	85.74	0.380	0.128	5.476e-04	0.0074
P2RY13	48.59	0.379	0.149	0.0019	0.0207
EIF4H	65.41	0.377	0.116	2.234e-04	0.0035
CD48	184.64	0.375	0.072	4.257e-08	1.571e-06
SNRPE	40.05	0.374	0.145	0.0018	0.0204
CCT8	48.20	0.374	0.138	0.0013	0.0154
SF3A1	40.63	0.373	0.146	0.0019	0.0205
CPVL	206.95	0.373	0.070	2.686e-08	1.025e-06
UBE2S	30.20	0.372	0.174	0.0055	0.0488
RSL1D1	77.40	0.370	0.126	6.702e-04	0.0088
UTRN	290.70	0.370	0.068	1.490e-08	6.011e-07
NHP2	44.62	0.369	0.144	0.0020	0.0213
FMN1	70.58	0.369	0.159	0.0036	0.0350
EIF4A1	403.54	0.369	0.054	1.401e-12	8.427e-11
PRKCA	41.25	0.368	0.147	0.0023	0.0242
ATP13A1	33.75	0.368	0.165	0.0047	0.0433
ATP5PO	117.67	0.368	0.087	4.747e-06	1.178e-04
ZNFX1	81.02	0.368	0.109	1.503e-04	0.0024
ZNF706	79.94	0.367	0.102	6.821e-05	0.0012
RPL18A	1647.33	0.366	0.041	1.540e-19	1.802e-17
VDAC3	33.38	0.364	0.169	0.0056	0.0493
RPL26	1814.81	0.361	0.041	1.581e-19	1.831e-17
ANP32B	118.07	0.361	0.088	9.215e-06	2.125e-04
SAMHD1	498.10	0.361	0.048	1.041e-14	7.733e-13
MYDGF	41.79	0.361	0.157	0.0042	0.0391
ESD	38.76	0.359	0.155	0.0037	0.0356
HLA-B	1789.95	0.359	0.039	3.049e-21	3.909e-19
NDUFA12	60.61	0.358	0.125	8.409e-04	0.0105
ERH	57.22	0.358	0.121	6.507e-04	0.0086
EIF3I	51.19	0.358	0.137	0.0018	0.0200
NUP62	32.36	0.358	0.165	0.0055	0.0486
FGL2	304.62	0.356	0.064	7.263e-09	3.128e-07
EIF4B	107.48	0.355	0.099	7.384e-05	0.0013
LAIR1	42.65	0.354	0.161	0.0054	0.0479
OXA1L	71.14	0.353	0.115	4.684e-04	0.0065
ATP5MC3	124.88	0.352	0.091	2.489e-05	5.125e-04
SLC25A3	167.88	0.352	0.089	1.854e-05	3.930e-04
CTSZ	220.39	0.352	0.067	3.529e-08	1.315e-06
PRDX1	76.95	0.352	0.119	6.996e-04	0.0091
TOMM20	69.94	0.351	0.117	6.012e-04	0.0080
PCBP1	214.98	0.348	0.066	3.294e-08	1.240e-06
NONO	62.25	0.348	0.124	0.0010	0.0124
CD63	129.79	0.348	0.092	4.104e-05	8.049e-04
HNRNPA0	54.36	0.347	0.134	0.0020	0.0218
CFL1	790.03	0.347	0.043	1.382e-16	1.200e-14
MCUB	81.43	0.345	0.114	5.553e-04	0.0075
UQCRC2	41.91	0.344	0.144	0.0034	0.0334
TUFM	66.56	0.343	0.131	0.0019	0.0211
HNRNPR	61.66	0.343	0.132	0.0021	0.0224
RPL17	800.69	0.342	0.041	1.142e-17	1.089e-15
SCIMP	59.19	0.340	0.120	0.0011	0.0132
EIF2S3	98.84	0.339	0.093	6.477e-05	0.0012
SAP18	90.44	0.338	0.102	2.395e-04	0.0037
RPL10	2661.35	0.338	0.047	2.131e-13	1.382e-11
HMOX1	60.05	0.338	0.127	0.0017	0.0195
ATP6V1F	152.90	0.337	0.093	7.400e-05	0.0013
FCGR1A	40.72	0.335	0.144	0.0043	0.0403
MYL12A	273.67	0.334	0.061	1.420e-08	5.791e-07
COMT	99.02	0.332	0.106	4.277e-04	0.0060
DYNLT1	129.43	0.332	0.095	1.212e-04	0.0020
RBCK1	65.95	0.331	0.120	0.0013	0.0157
VIM	1783.65	0.331	0.051	3.565e-11	1.920e-09
TRAM1	59.35	0.331	0.125	0.0017	0.0196
GSTK1	159.90	0.330	0.076	3.849e-06	9.775e-05
EIF4A2	45.64	0.330	0.141	0.0046	0.0419
PSMA2	62.83	0.329	0.121	0.0016	0.0185
SLC7A7	123.65	0.328	0.100	2.571e-04	0.0039
RPS20	195.04	0.327	0.077	5.553e-06	1.350e-04
HSPA1A	56.77	0.326	0.133	0.0035	0.0335
UBXN1	121.95	0.324	0.086	4.923e-05	9.301e-04
RPS23	1633.09	0.323	0.044	3.481e-14	2.425e-12
RAN	78.00	0.323	0.110	8.498e-04	0.0106

Gene	baseMean	log2FC	lfcSE	p-value	padj
PSMB2	46.50	0.321	0.140	0.0052	0.0463
MAF1	46.96	0.320	0.138	0.0048	0.0436
RPS9	1536.38	0.320	0.034	4.117e-22	5.612e-20
PARK7	103.62	0.320	0.096	2.401e-04	0.0037
CD68	239.75	0.318	0.063	1.769e-07	5.807e-06
RPL13A	722.29	0.318	0.048	1.348e-11	7.599e-10
PSMB6	78.56	0.317	0.105	6.684e-04	0.0088
C20orf27	89.14	0.317	0.107	8.471e-04	0.0106
NUCKS1	50.08	0.317	0.128	0.0034	0.0328
PHB2	71.53	0.313	0.126	0.0033	0.0322
ZFAS1	211.05	0.312	0.073	5.796e-06	1.403e-04
EIF3H	174.42	0.312	0.073	5.957e-06	1.438e-04
RPL14	786.61	0.309	0.043	3.221e-13	2.065e-11
LYZ	3421.60	0.309	0.042	2.748e-14	1.934e-12
CANX	102.13	0.307	0.094	3.414e-04	0.0050
ALDOA	204.41	0.306	0.086	1.106e-04	0.0019
IGSF6	126.11	0.306	0.091	2.372e-04	0.0037
PLEKHO1	187.65	0.305	0.072	6.933e-06	1.648e-04
RBBP6	61.01	0.305	0.125	0.0041	0.0384
GIMAP1	72.46	0.305	0.112	0.0018	0.0198
RPS19	1558.47	0.304	0.039	1.690e-15	1.358e-13
KCTD12	145.65	0.303	0.079	4.195e-05	8.212e-04
DNAJA1	72.01	0.301	0.124	0.0040	0.0382
GAPDH	1495.47	0.298	0.051	1.530e-09	6.951e-08
LMO2	94.35	0.298	0.111	0.0021	0.0221
ATP5F1C	78.31	0.297	0.110	0.0018	0.0202
SERPINA1	372.32	0.297	0.054	1.450e-08	5.891e-07
VPS35	56.98	0.297	0.127	0.0051	0.0460
PSAP	1107.69	0.295	0.035	1.310e-17	1.238e-15
NAP1L1	145.53	0.295	0.081	9.104e-05	0.0016
HLA-C	1113.00	0.295	0.039	1.921e-14	1.370e-12
CD52	371.22	0.294	0.058	1.730e-07	5.714e-06
COX6C	132.89	0.293	0.091	3.942e-04	0.0056
RPL29	1369.76	0.292	0.045	2.808e-11	1.543e-09
ZFP36L2	432.26	0.291	0.059	2.657e-07	8.364e-06
CAPG	167.07	0.288	0.079	8.231e-05	0.0014
HNRNPF	88.60	0.286	0.101	0.0014	0.0162
NDUFA4	122.35	0.285	0.088	3.880e-04	0.0055
UQCRFS1	85.81	0.285	0.101	0.0015	0.0175
DUSP6	274.94	0.283	0.084	2.695e-04	0.0041
AFF1	88.82	0.281	0.101	0.0018	0.0202
NACA	846.80	0.280	0.044	4.955e-11	2.602e-09
CLTA	134.54	0.277	0.080	1.979e-04	0.0031
NAGK	172.13	0.277	0.073	5.533e-05	0.0010
ARHGDIB	381.44	0.276	0.053	7.285e-08	2.589e-06
CTSB	251.71	0.276	0.072	4.216e-05	8.238e-04
PDIA3	78.55	0.275	0.116	0.0056	0.0493
CHCHD10	101.93	0.274	0.111	0.0045	0.0413
ITGB2	490.27	0.274	0.053	9.715e-08	3.419e-06
EIF3G	165.87	0.270	0.076	1.351e-04	0.0022
RPS3	1144.24	0.270	0.042	2.768e-11	1.529e-09
HADHA	105.14	0.269	0.101	0.0027	0.0273
RPL7A	1132.05	0.269	0.047	3.470e-09	1.538e-07
EIF3M	106.55	0.262	0.090	0.0013	0.0155
ATG3	96.82	0.261	0.100	0.0032	0.0314
RPS12	2455.46	0.260	0.048	2.991e-08	1.134e-06
YBX3	114.96	0.259	0.089	0.0013	0.0155
LAMTOR2	87.32	0.258	0.101	0.0038	0.0361
PRKCE	113.91	0.257	0.092	0.0019	0.0212
S100A10	1003.92	0.257	0.061	1.067e-05	2.385e-04
FYB1	476.04	0.256	0.055	1.308e-06	3.706e-05
RPL9	842.29	0.254	0.051	2.673e-07	8.392e-06
RPL6	1162.51	0.253	0.050	1.456e-07	4.898e-06
NDUFS7	80.85	0.252	0.102	0.0050	0.0454
HNRNPK	165.37	0.252	0.073	2.227e-04	0.0035
POMP	88.34	0.246	0.096	0.0042	0.0391
NCOA4	149.30	0.246	0.077	6.248e-04	0.0083
SCLT1	139.85	0.246	0.084	0.0014	0.0158
PSME1	220.42	0.243	0.074	4.360e-04	0.0061
ERP29	164.44	0.242	0.072	3.503e-04	0.0051
RPL31	426.71	0.241	0.053	2.254e-06	6.114e-05
AHR	220.20	0.240	0.086	0.0023	0.0240
HINT1	156.75	0.239	0.076	6.874e-04	0.0090
GDI2	130.14	0.239	0.083	0.0016	0.0183
CRIP1	759.77	0.239	0.059	2.343e-05	4.870e-04

Gene	baseMean	log2FC	lfcSE	p-value	padj
RPL37	1784.01	0.239	0.040	1.230e-09	5.711e-08
RPL24	663.28	0.238	0.048	3.626e-07	1.119e-05
CFP	224.00	0.238	0.069	2.743e-04	0.0041
RPS3A	1431.18	0.236	0.044	2.237e-08	8.666e-07
JUNB	279.71	0.234	0.076	8.749e-04	0.0108
COX5A	128.61	0.232	0.085	0.0027	0.0279
TMSB4X	4829.76	0.229	0.035	4.059e-11	2.164e-09
CALM1	245.57	0.227	0.062	1.059e-04	0.0018
RPL19	2000.37	0.226	0.040	1.099e-08	4.569e-07
ZEB2	775.17	0.225	0.049	2.563e-06	6.781e-05
ZYX	341.32	0.225	0.055	2.762e-05	5.643e-04
COX7C	282.23	0.224	0.058	5.416e-05	0.0010
LCP2	112.07	0.224	0.090	0.0053	0.0471
ZNF385A	122.74	0.222	0.085	0.0039	0.0370
CSDE1	149.24	0.221	0.077	0.0020	0.0219
RPUSD2	2.41	0.221	0.417	0.0039	0.0375
RPS17	285.88	0.219	0.062	1.899e-04	0.0030
EDF1	183.99	0.218	0.073	0.0013	0.0151
RPL23	307.99	0.218	0.060	1.540e-04	0.0025
GSTP1	606.55	0.218	0.046	1.420e-06	3.991e-05
TMBIM6	152.19	0.216	0.077	0.0023	0.0244
RPL21	1046.29	0.216	0.042	1.198e-07	4.095e-06
PABPC1	759.32	0.210	0.046	2.172e-06	5.951e-05
BRK1	148.02	0.209	0.077	0.0034	0.0328
IFI30	1425.75	0.209	0.042	2.607e-07	8.233e-06
ACTB	4939.24	0.208	0.038	2.668e-08	1.023e-06
MYL12B	175.33	0.201	0.071	0.0024	0.0249
RPS15	1643.83	0.200	0.052	7.775e-05	0.0014
RPL8	1259.11	0.195	0.042	2.308e-06	6.199e-05
NRP2	2.23	0.195	0.355	0.0050	0.0454
RPS8	2165.60	0.187	0.049	7.666e-05	0.0014
RPS14	2255.51	0.184	0.041	3.911e-06	9.909e-05
RPS11	578.92	0.184	0.049	9.250e-05	0.0016
DDX5	331.79	0.184	0.057	7.227e-04	0.0093
CARD16	202.35	0.180	0.066	0.0035	0.0335
PFN1	1109.43	0.180	0.034	8.611e-08	3.050e-06
MARCH1	213.01	0.179	0.063	0.0028	0.0283
RPS27A	1382.06	0.179	0.038	1.989e-06	5.478e-05
RPS15A	1709.91	0.177	0.037	1.322e-06	3.737e-05
VAMP8	273.10	0.176	0.063	0.0036	0.0344
RPL36	967.12	0.173	0.041	1.518e-05	3.302e-04
RPL32	2141.06	0.173	0.039	7.719e-06	1.819e-04
RPL18	1353.80	0.170	0.043	5.645e-05	0.0010
CST3	1702.62	0.169	0.043	4.676e-05	8.960e-04
MSN	247.06	0.165	0.061	0.0042	0.0391
CYBB	415.25	0.164	0.049	5.574e-04	0.0075
B2M	3203.99	0.162	0.049	6.337e-04	0.0084
FLNA	379.01	0.161	0.059	0.0042	0.0395
CLIC1	396.28	0.160	0.054	0.0019	0.0208
LGALS1	805.23	0.159	0.046	3.412e-04	0.0050
RPL41	3971.19	0.158	0.034	2.382e-06	6.349e-05
EIF1	1128.69	0.152	0.044	4.341e-04	0.0061
HLA-E	529.54	0.148	0.052	0.0026	0.0269
RPS24	2116.74	0.145	0.044	7.047e-04	0.0091
AHNAK	831.32	0.144	0.048	0.0021	0.0223
FCN1	1331.61	0.143	0.052	0.0042	0.0391
RPL35A	1384.14	0.142	0.036	8.689e-05	0.0015
TAGLN2	461.90	0.140	0.050	0.0038	0.0364
RPL11	1823.90	0.140	0.040	2.980e-04	0.0044
LAPTM5	611.26	0.137	0.050	0.0049	0.0445
PTPRC	555.54	0.131	0.043	0.0015	0.0174
RPS21	1062.32	0.130	0.038	5.500e-04	0.0074
UBA52	1036.95	0.129	0.045	0.0033	0.0323
RPL22	728.82	0.125	0.042	0.0023	0.0240
RPL12	1414.50	0.123	0.045	0.0053	0.0473
RPS13	2001.03	0.122	0.044	0.0048	0.0437
RPS7	1263.22	0.117	0.042	0.0042	0.0393
TMSB10	4180.12	0.108	0.035	0.0020	0.0216
SRGN	700.03	-0.123	0.045	0.0051	0.0457
FTH1	6291.29	-0.126	0.043	0.0037	0.0353
LYN	527.21	-0.134	0.044	0.0020	0.0219
FTL	9579.72	-0.135	0.031	1.107e-05	2.468e-04
EEF1D	489.49	-0.137	0.049	0.0040	0.0377
HBB	151.27	-0.142	0.276	0.0036	0.0346
TMA7	384.38	-0.144	0.052	0.0046	0.0422
SERF2	1375.42	-0.149	0.037	3.493e-05	6.972e-04
RPL39	2546.37	-0.152	0.047	8.277e-04	0.0104

Gene	baseMean	log2FC	lfcSE	p-value	padj
AKAP13	452.78	-0.152	0.046	7.416e-04	0.0095
LRMDA	366.82	-0.161	0.061	0.0052	0.0466
NCF1	626.80	-0.163	0.055	0.0023	0.0240
MT-ATP6	2916.50	-0.165	0.058	0.0027	0.0277
DOCK2	281.39	-0.166	0.058	0.0029	0.0289
MYL6	904.26	-0.168	0.039	1.373e-05	3.013e-04
LST1	622.70	-0.173	0.049	3.026e-04	0.0045
RNASEK	645.67	-0.174	0.050	3.515e-04	0.0051
PRKCB	400.86	-0.175	0.055	9.072e-04	0.0112
TSPO	795.86	-0.176	0.058	0.0015	0.0177
GAS7	252.31	-0.177	0.064	0.0034	0.0333
CTSD	402.39	-0.178	0.056	9.541e-04	0.0116
S100A4	2828.62	-0.180	0.035	1.836e-07	5.972e-06
BLOC1S1	216.78	-0.185	0.069	0.0043	0.0396
PGLS	213.29	-0.195	0.069	0.0027	0.0278
LRRFIP1	546.35	-0.195	0.046	1.464e-05	3.192e-04
FKBP5	623.45	-0.196	0.053	7.900e-05	0.0014
IL17RA	271.02	-0.196	0.064	0.0013	0.0150
DMXL2	248.01	-0.197	0.069	0.0025	0.0257
UBL5	253.80	-0.198	0.064	0.0012	0.0138
MT-ND5	1029.03	-0.198	0.048	2.024e-05	4.248e-04
RIN3	214.91	-0.199	0.068	0.0014	0.0165
ACAP2	297.91	-0.200	0.062	7.504e-04	0.0096
ARAP1	151.75	-0.202	0.079	0.0055	0.0488
PIAS1	177.70	-0.206	0.078	0.0045	0.0411
MYO1G	162.08	-0.209	0.082	0.0055	0.0486
TAOK3	204.48	-0.209	0.070	0.0013	0.0151
NEAT1	1262.47	-0.210	0.041	1.121e-07	3.881e-06
MT-ND3	2449.61	-0.212	0.042	3.371e-07	1.055e-05
RPS26	1294.44	-0.213	0.049	8.603e-06	2.001e-04
ADGRE5	399.50	-0.214	0.057	7.600e-05	0.0014
ARHGAP15	188.48	-0.215	0.074	0.0019	0.0205
CELF2	478.76	-0.217	0.060	1.374e-04	0.0023
MED13L	275.96	-0.218	0.064	3.483e-04	0.0050
IQGAP1	325.97	-0.220	0.055	3.196e-05	6.433e-04
DISC1	144.59	-0.220	0.082	0.0034	0.0334
MALAT1	4152.51	-0.221	0.046	1.001e-06	2.897e-05
GCA	159.11	-0.222	0.079	0.0023	0.0243
MRTFA	113.05	-0.225	0.087	0.0045	0.0417
CCNY	187.87	-0.225	0.071	7.568e-04	0.0096
CREBBP	130.56	-0.226	0.084	0.0034	0.0329
TBC1D22A	166.20	-0.227	0.082	0.0027	0.0274
PICALM	328.66	-0.228	0.068	4.244e-04	0.0060
TSC22D3	353.20	-0.228	0.063	1.454e-04	0.0024
DDX17	216.38	-0.228	0.069	4.544e-04	0.0063
LYST	319.77	-0.228	0.065	2.202e-04	0.0035
AGFG1	138.24	-0.229	0.083	0.0027	0.0273
KDM2A	176.70	-0.230	0.083	0.0026	0.0269
ANKRD44	230.89	-0.231	0.068	3.264e-04	0.0048
BAZ2B	140.27	-0.231	0.086	0.0030	0.0303
COP1	205.72	-0.231	0.067	2.793e-04	0.0042
PTPRE	298.35	-0.234	0.069	2.069e-04	0.0033
TNFAIP2	397.59	-0.235	0.058	2.504e-05	5.145e-04
ARHGEF1	144.53	-0.236	0.078	0.0011	0.0131
ATXN1	140.32	-0.236	0.083	0.0019	0.0211
FMNL1	240.32	-0.240	0.063	7.502e-05	0.0013
GMFG	341.74	-0.240	0.060	3.111e-05	6.284e-04
MAPK1	136.91	-0.242	0.087	0.0023	0.0244
ROCK1	236.15	-0.243	0.065	8.686e-05	0.0015
ERGIC1	94.25	-0.245	0.099	0.0054	0.0479
GRK3	167.03	-0.246	0.075	4.287e-04	0.0060
CAB39	113.65	-0.247	0.093	0.0033	0.0326
SEC62	94.06	-0.247	0.094	0.0033	0.0324
GRK2	233.24	-0.247	0.064	4.705e-05	8.998e-04
AKAP9	110.10	-0.248	0.089	0.0020	0.0219
AGTRAP	185.86	-0.248	0.071	1.841e-04	0.0029
EHBP1L1	189.87	-0.249	0.082	9.664e-04	0.0118
SP3	93.94	-0.249	0.099	0.0045	0.0416
JAML	300.63	-0.249	0.058	9.485e-06	2.164e-04
TBCA	113.55	-0.250	0.089	0.0021	0.0224
SGK3	123.69	-0.250	0.091	0.0025	0.0258
OST4	444.61	-0.250	0.054	1.756e-06	4.873e-05
TTC7A	160.44	-0.252	0.078	5.286e-04	0.0072
HK3	161.45	-0.253	0.080	5.901e-04	0.0079
FAM49B	264.97	-0.253	0.066	4.821e-05	9.124e-04

Gene	baseMean	log2FC	lfcSE	p-value	padj
SNX29	146.10	-0.254	0.083	9.046e-04	0.0111
STAT6	112.19	-0.254	0.090	0.0017	0.0190
TMTC2	119.57	-0.259	0.093	0.0020	0.0218
RIPOR2	471.82	-0.259	0.051	1.833e-07	5.972e-06
STK17B	429.32	-0.259	0.062	1.205e-05	2.658e-04
PLCB2	123.93	-0.259	0.091	0.0017	0.0193
NDUFA3	120.27	-0.260	0.087	0.0011	0.0127
FNIP1	79.32	-0.261	0.108	0.0056	0.0492
RBM6	126.23	-0.262	0.085	8.168e-04	0.0103
OSCAR	131.98	-0.262	0.089	0.0013	0.0150
USP32	106.94	-0.263	0.092	0.0018	0.0202
PIK3R5	213.32	-0.263	0.082	5.060e-04	0.0069
RCOR1	105.81	-0.265	0.099	0.0027	0.0272
LRRK2	231.53	-0.265	0.065	1.598e-05	3.469e-04
MARK2	75.60	-0.266	0.105	0.0041	0.0385
FGD4	171.17	-0.268	0.083	4.907e-04	0.0067
TNRC6B	149.94	-0.268	0.078	2.399e-04	0.0037
ICAM3	128.39	-0.269	0.093	0.0013	0.0156
FAM200B	85.11	-0.271	0.108	0.0042	0.0392
FYN	215.82	-0.272	0.072	5.789e-05	0.0011
PLXDC2	636.88	-0.274	0.060	1.802e-06	4.987e-05
ZFAND3	296.40	-0.274	0.058	1.004e-06	2.899e-05
MAML3	197.31	-0.275	0.072	4.753e-05	9.047e-04
TRAPPC9	99.48	-0.276	0.104	0.0027	0.0277
H2AFZ	101.96	-0.277	0.093	9.883e-04	0.0120
SLC12A6	75.63	-0.278	0.113	0.0045	0.0412
STIM1	82.27	-0.279	0.103	0.0024	0.0246
SNRNP70	108.03	-0.279	0.091	7.590e-04	0.0097
UQCR11	404.61	-0.279	0.052	2.292e-08	8.845e-07
AGTPBP1	210.21	-0.280	0.073	5.174e-05	9.705e-04
NFAM1	108.31	-0.280	0.100	0.0017	0.0190
AKNA	133.38	-0.280	0.090	6.374e-04	0.0085
BABAM2	86.90	-0.281	0.104	0.0023	0.0245
RCSD1	208.56	-0.282	0.074	4.481e-05	8.678e-04
FNDC3B	192.30	-0.283	0.082	1.892e-04	0.0030
MON2	70.36	-0.285	0.115	0.0041	0.0385
ZNF609	116.90	-0.285	0.095	8.420e-04	0.0105
SIK3	325.65	-0.285	0.060	8.193e-07	2.404e-05
SULT1A1	76.08	-0.285	0.108	0.0025	0.0262
TGFBR2	156.35	-0.285	0.086	2.938e-04	0.0044
ITGAM	127.48	-0.286	0.092	6.567e-04	0.0087
PKN2	140.92	-0.286	0.081	1.470e-04	0.0024
GAB2	268.87	-0.288	0.062	1.244e-06	3.554e-05
FBXW7	145.60	-0.290	0.079	8.705e-05	0.0015
CORO7	71.44	-0.290	0.119	0.0044	0.0410
PPP6R3	91.98	-0.291	0.110	0.0027	0.0275
TRIOBP	68.74	-0.291	0.115	0.0032	0.0318
RBM47	348.06	-0.292	0.066	2.934e-06	7.632e-05
SLC43A2	100.99	-0.293	0.098	9.482e-04	0.0116
MEGF9	319.44	-0.293	0.071	1.120e-05	2.492e-04
ATP2B1	276.37	-0.294	0.073	1.990e-05	4.185e-04
LENG8	105.42	-0.295	0.105	0.0015	0.0175
CYTH1	152.20	-0.295	0.082	1.159e-04	0.0019
PLCB1	326.08	-0.295	0.060	2.407e-07	7.712e-06
POU2F2	272.93	-0.297	0.067	2.919e-06	7.629e-05
STK10	224.57	-0.298	0.067	2.989e-06	7.756e-05
ELF2	145.27	-0.298	0.080	7.083e-05	0.0013
CPQ	103.29	-0.298	0.098	6.846e-04	0.0089
MPP7	114.05	-0.299	0.113	0.0023	0.0244
MICU1	111.54	-0.300	0.095	4.925e-04	0.0068
MT-CO1	8755.26	-0.301	0.052	2.521e-09	1.136e-07
BRAF	70.50	-0.302	0.118	0.0029	0.0289
JARID2	301.45	-0.302	0.069	4.239e-06	1.067e-04
RC3H1	123.23	-0.302	0.085	1.185e-04	0.0020
CYTH4	109.65	-0.302	0.099	6.489e-04	0.0086
ARL15	149.38	-0.303	0.089	1.972e-04	0.0031
NUMB	262.88	-0.303	0.060	1.191e-07	4.083e-06
SH3KBP1	219.34	-0.303	0.073	1.006e-05	2.266e-04
PHF20	103.90	-0.304	0.100	7.468e-04	0.0096
GPATCH8	77.73	-0.304	0.111	0.0019	0.0205
DNM2	119.74	-0.304	0.092	2.771e-04	0.0042
UBXN11	166.92	-0.304	0.080	5.007e-05	9.425e-04
SEMA4D	97.53	-0.304	0.100	6.701e-04	0.0088
CCDC69	125.20	-0.305	0.087	1.310e-04	0.0022
S100A6	3536.89	-0.305	0.031	7.342e-23	1.054e-20
USP4	119.59	-0.306	0.091	2.400e-04	0.0037
LAMTOR4	366.85	-0.306	0.057	3.222e-08	1.217e-06

Gene	baseMean	log2FC	lfcSE	p-value	padj
ARHGAP45	122.75	-0.307	0.088	1.486e-04	0.0024
SPECC1	64.19	-0.307	0.120	0.0030	0.0303
TBC1D8	89.48	-0.307	0.119	0.0028	0.0283
NLRP1	169.99	-0.308	0.084	7.256e-05	0.0013
CELF1	177.65	-0.308	0.085	9.572e-05	0.0016
COLGALT1	61.48	-0.308	0.134	0.0056	0.0493
FTX	139.49	-0.309	0.081	4.715e-05	9.002e-04
MT-ND1	3125.43	-0.310	0.041	8.284e-15	6.327e-13
ARGLU1	131.81	-0.311	0.094	2.797e-04	0.0042
MBP	176.08	-0.311	0.071	3.712e-06	9.494e-05
ASGR2	71.04	-0.314	0.117	0.0021	0.0221
CPD	79.05	-0.314	0.128	0.0037	0.0356
PPP2R5A	58.28	-0.314	0.125	0.0032	0.0313
MT-ND4	2701.78	-0.315	0.043	5.264e-14	3.610e-12
RELB	88.23	-0.315	0.110	0.0012	0.0138
DGKD	74.55	-0.315	0.112	0.0014	0.0161
LSM6	68.98	-0.316	0.117	0.0018	0.0204
MT-CO3	4637.67	-0.317	0.046	2.179e-12	1.275e-10
KDM6A	95.48	-0.317	0.102	5.052e-04	0.0069
ATG7	283.49	-0.318	0.063	1.401e-07	4.728e-06
ATP5F1E	1387.30	-0.318	0.037	1.458e-18	1.495e-16
CD55	153.32	-0.319	0.079	1.702e-05	3.650e-04
UBAP1	77.62	-0.321	0.110	9.284e-04	0.0114
DENND10	73.51	-0.321	0.110	9.757e-04	0.0119
SOD2	159.14	-0.321	0.086	5.326e-05	9.939e-04
PLBD1	169.50	-0.323	0.097	2.380e-04	0.0037
TLE4	104.02	-0.323	0.100	3.158e-04	0.0047
ARID3A	70.86	-0.325	0.118	0.0015	0.0177
MAP2K3	77.61	-0.325	0.113	0.0011	0.0127
TMCC1	57.93	-0.328	0.123	0.0020	0.0217
P2RY8	63.33	-0.328	0.133	0.0034	0.0329
CMIP	204.67	-0.328	0.074	2.711e-06	7.155e-05
TCIRG1	257.10	-0.328	0.080	1.067e-05	2.385e-04
ATG16L2	166.00	-0.329	0.082	1.919e-05	4.044e-04
HRH2	115.51	-0.329	0.092	9.361e-05	0.0016
MSRB1	78.84	-0.329	0.111	7.512e-04	0.0096
SIPA1L3	55.38	-0.329	0.135	0.0033	0.0323
VAV1	61.94	-0.329	0.135	0.0034	0.0334
KIF13A	147.22	-0.330	0.086	3.103e-05	6.280e-04
STAG1	110.10	-0.330	0.099	2.138e-04	0.0034
ENTPD1	114.05	-0.330	0.092	8.544e-05	0.0015
PARP8	120.26	-0.330	0.089	6.133e-05	0.0011
FOXP1	354.93	-0.330	0.060	1.139e-08	4.699e-07
MAP3K3	174.33	-0.331	0.073	1.668e-06	4.654e-05
PYGL	70.74	-0.331	0.119	0.0013	0.0155
KLHL18	49.70	-0.332	0.145	0.0050	0.0448
PLAUR	113.20	-0.332	0.096	1.433e-04	0.0023
STK40	63.44	-0.332	0.117	0.0011	0.0136
TOM1	130.21	-0.332	0.097	1.596e-04	0.0026
UBE4B	54.27	-0.333	0.134	0.0031	0.0310
RASSF3	129.82	-0.333	0.091	6.370e-05	0.0012
NFAT5	76.77	-0.333	0.113	8.155e-04	0.0103
SLC22A15	56.45	-0.333	0.130	0.0024	0.0252
CCNL2	45.19	-0.335	0.146	0.0046	0.0422
RNF144B	188.61	-0.335	0.089	4.574e-05	8.827e-04
HDAC4	104.66	-0.335	0.095	1.043e-04	0.0018
SIPA1L1	282.86	-0.336	0.082	9.299e-06	2.140e-04
SPAG9	102.27	-0.336	0.101	2.282e-04	0.0035
SEC14L1	83.37	-0.339	0.117	8.960e-04	0.0110
PAXBP1	42.25	-0.340	0.149	0.0049	0.0444
FBXO42	48.42	-0.340	0.140	0.0034	0.0334
FOXO3	157.29	-0.341	0.105	2.745e-04	0.0041
INPP5D	162.23	-0.341	0.085	1.643e-05	3.540e-04
KPNA6	43.00	-0.341	0.144	0.0041	0.0383
TREM1	76.01	-0.343	0.117	8.133e-04	0.0103
ADAM8	42.60	-0.345	0.149	0.0043	0.0403
CYP1B1	84.80	-0.345	0.112	4.837e-04	0.0067
MYO15B	79.11	-0.345	0.109	3.701e-04	0.0053
STAT5B	48.03	-0.346	0.140	0.0029	0.0293
GATAD2A	73.43	-0.349	0.112	4.273e-04	0.0060
ITGB2-AS1	68.73	-0.350	0.119	7.730e-04	0.0098
HGS	54.95	-0.350	0.141	0.0029	0.0288
ARHGAP24	184.75	-0.350	0.075	9.900e-07	2.873e-05
SMARCD3	93.76	-0.350	0.110	3.428e-04	0.0050
AGO2	47.95	-0.350	0.142	0.0029	0.0288

Gene	baseMean	log2FC	lfcSE	p-value	padj
IRAK3	332.97	-0.350	0.058	3.469e-10	1.705e-08
KLF7	53.85	-0.351	0.148	0.0036	0.0348
PPM1B	85.66	-0.351	0.111	3.834e-04	0.0055
NIN	115.62	-0.351	0.092	3.320e-05	6.656e-04
RAF1	86.63	-0.351	0.103	1.501e-04	0.0024
PPP2R2D	45.11	-0.353	0.143	0.0030	0.0296
MED15	65.26	-0.353	0.124	9.568e-04	0.0117
FOSL2	47.00	-0.354	0.151	0.0039	0.0373
MAPK14	113.11	-0.354	0.101	9.842e-05	0.0017
SULF2	135.70	-0.354	0.095	4.821e-05	9.124e-04
PREX1	235.31	-0.354	0.071	1.592e-07	5.323e-06
STXBP2	427.63	-0.354	0.056	7.541e-11	3.885e-09
PPM1F	66.80	-0.355	0.136	0.0019	0.0211
MYO1F	522.57	-0.355	0.046	1.271e-14	9.311e-13
TANGO2	39.34	-0.357	0.159	0.0048	0.0438
QSOX1	61.17	-0.358	0.137	0.0019	0.0207
ELOB	420.05	-0.359	0.050	2.964e-13	1.911e-11
NBPF19	59.35	-0.360	0.125	8.747e-04	0.0108
RASGRP2	77.61	-0.360	0.111	2.493e-04	0.0038
RSBN1L	67.47	-0.361	0.115	3.891e-04	0.0055
RETN	41.81	-0.362	0.168	0.0055	0.0488
DYSF	110.10	-0.362	0.097	3.995e-05	7.894e-04
ERICH1	132.66	-0.363	0.101	7.401e-05	0.0013
TLE3	43.15	-0.366	0.153	0.0031	0.0309
C5AR1	110.93	-0.367	0.097	3.698e-05	7.348e-04
MGRN1	66.58	-0.367	0.118	3.617e-04	0.0052
RPS6KA3	104.32	-0.367	0.097	3.242e-05	6.513e-04
KPNA1	37.60	-0.367	0.170	0.0054	0.0479
R3HDM2	127.11	-0.367	0.090	9.711e-06	2.206e-04
RNF145	68.36	-0.368	0.124	6.745e-04	0.0088
SMG6	41.43	-0.368	0.158	0.0037	0.0356
CUX1	290.61	-0.369	0.067	8.261e-09	3.516e-07
SMIM25	98.23	-0.370	0.105	9.213e-05	0.0016
PRKAG2	172.63	-0.370	0.090	7.839e-06	1.839e-04
WDR37	47.42	-0.372	0.152	0.0027	0.0276
ARRDC1	76.61	-0.372	0.128	7.500e-04	0.0096
AGO4	115.55	-0.373	0.094	1.706e-05	3.653e-04
TTLL3	33.28	-0.373	0.174	0.0055	0.0483
TYK2	84.91	-0.373	0.108	1.229e-04	0.0020
ABHD3	49.52	-0.374	0.142	0.0016	0.0184
ANKRD13D	128.19	-0.375	0.093	1.166e-05	2.588e-04
SSH2	485.76	-0.375	0.054	1.356e-12	8.202e-11
PPP6R2	69.32	-0.376	0.126	5.739e-04	0.0077
AC008105.3	47.24	-0.377	0.143	0.0017	0.0189
NDE1	55.74	-0.377	0.132	8.423e-04	0.0105
ELL	41.92	-0.377	0.163	0.0035	0.0340
CSF2RA	127.56	-0.379	0.090	5.597e-06	1.357e-04
CTBP2	110.09	-0.380	0.092	8.544e-06	1.991e-04
ARHGAP26	608.57	-0.380	0.046	2.349e-17	2.144e-15
TMEM91	56.87	-0.381	0.133	7.698e-04	0.0098
ST3GAL1	68.34	-0.381	0.124	4.216e-04	0.0059
TCF25	238.01	-0.382	0.072	2.065e-08	8.130e-07
DENND3	68.01	-0.384	0.117	1.990e-04	0.0031
BCL6	71.91	-0.385	0.126	4.156e-04	0.0059
FOXO1	59.35	-0.385	0.140	0.0011	0.0133
SYNE3	38.43	-0.386	0.157	0.0025	0.0256
CD93	122.92	-0.386	0.110	8.505e-05	0.0015
ARHGAP4	154.18	-0.386	0.078	1.285e-07	4.378e-06
PSTPIP1	113.33	-0.386	0.103	3.331e-05	6.667e-04
NFATC3	58.60	-0.387	0.142	0.0012	0.0140
MFSD14C	58.26	-0.387	0.138	9.456e-04	0.0116
ITGAX	108.67	-0.388	0.094	8.191e-06	1.913e-04
ATP11A	113.33	-0.388	0.091	4.401e-06	1.102e-04
TRAF3	85.91	-0.389	0.113	1.158e-04	0.0019
ZNF516	64.62	-0.389	0.124	3.401e-04	0.0050
ZNF407	46.11	-0.389	0.145	0.0013	0.0153
RAB11FIP1	272.55	-0.389	0.066	8.255e-10	3.899e-08
MT-ND4L	949.29	-0.390	0.051	6.714e-15	5.239e-13
LUC7L3	103.55	-0.392	0.101	2.059e-05	4.304e-04
TXNRD2	35.18	-0.392	0.167	0.0031	0.0310
TBL1X	137.61	-0.392	0.093	4.486e-06	1.119e-04
HDAC7	43.64	-0.392	0.166	0.0030	0.0303
VMP1	451.44	-0.393	0.054	8.070e-14	5.466e-12
MPHOSPH8	69.39	-0.394	0.116	1.341e-04	0.0022
RTN3	146.08	-0.395	0.095	6.241e-06	1.500e-04
RBM5	62.14	-0.395	0.121	2.269e-04	0.0035
YPEL3	175.53	-0.395	0.080	1.679e-07	5.563e-06

Gene	baseMean	log2FC	lfcSE	p-value	padj
GLT1D1	139.32	-0.396	0.089	1.750e-06	4.870e-05
SNW1	36.68	-0.398	0.166	0.0026	0.0270
MAN2A2	39.07	-0.398	0.156	0.0018	0.0203
AP3S2	59.28	-0.399	0.132	4.359e-04	0.0061
RFX1	28.19	-0.400	0.191	0.0055	0.0488
RABGEF1	113.58	-0.400	0.108	4.086e-05	8.029e-04
RLF	53.68	-0.401	0.133	4.623e-04	0.0064
ANKRD11	181.87	-0.403	0.076	2.345e-08	9.019e-07
ATP2B4	37.03	-0.409	0.175	0.0029	0.0288
LPCAT1	33.34	-0.409	0.191	0.0046	0.0419
SMG5	39.36	-0.409	0.161	0.0018	0.0200
PACS1	81.54	-0.409	0.111	4.402e-05	8.571e-04
USP48	56.56	-0.411	0.138	4.792e-04	0.0066
FRY	138.86	-0.411	0.085	2.495e-07	7.929e-06
PTPRJ	196.47	-0.411	0.074	4.813e-09	2.107e-07
CAMKK2	95.91	-0.413	0.101	8.098e-06	1.896e-04
PLXNC1	105.37	-0.413	0.102	9.474e-06	2.164e-04
DEF8	52.63	-0.414	0.131	2.841e-04	0.0042
CARD8	55.79	-0.414	0.139	5.065e-04	0.0069
SLC49A4	45.93	-0.419	0.148	7.052e-04	0.0091
CLASRP	72.04	-0.422	0.119	6.737e-05	0.0012
PHF21A	146.61	-0.422	0.088	3.858e-07	1.177e-05
RNF24	77.34	-0.423	0.116	4.503e-05	8.705e-04
VPS9D1	31.97	-0.424	0.184	0.0030	0.0303
IGF2R	133.73	-0.424	0.089	3.418e-07	1.067e-05
SLC16A6	23.34	-0.425	0.202	0.0047	0.0426
SOS2	93.87	-0.427	0.111	1.903e-05	4.025e-04
KIAA0319L	85.42	-0.429	0.112	2.157e-05	4.502e-04
PPP3CA	149.18	-0.429	0.082	4.051e-08	1.504e-06
PHKG2	34.28	-0.430	0.172	0.0018	0.0198
CDC42EP3	140.08	-0.431	0.083	4.259e-08	1.571e-06
ACTN1	78.00	-0.433	0.109	1.170e-05	2.592e-04
EPB41	66.71	-0.433	0.125	8.527e-05	0.0015
FAM126B	30.94	-0.433	0.179	0.0021	0.0226
MIR29B2CHG	35.39	-0.434	0.167	0.0014	0.0163
CREB5	152.25	-0.436	0.090	2.274e-07	7.333e-06
S100Z	88.47	-0.437	0.115	2.422e-05	5.006e-04
DOCK5	147.04	-0.438	0.103	3.440e-06	8.861e-05
SRPK2	187.28	-0.438	0.082	1.535e-08	6.166e-07
TSHZ3	35.30	-0.438	0.163	0.0010	0.0126
CWC25	45.58	-0.439	0.146	4.123e-04	0.0058
RAP1GAP2	130.62	-0.440	0.110	9.347e-06	2.146e-04
SH3GL1	35.19	-0.442	0.169	0.0013	0.0155
ST6GALNAC3	84.24	-0.442	0.113	1.382e-05	3.026e-04
PER1	139.85	-0.442	0.104	3.602e-06	9.236e-05
PPARD	24.57	-0.443	0.203	0.0037	0.0356
SH3TC1	72.89	-0.446	0.116	1.799e-05	3.836e-04
B4GALT1	73.53	-0.446	0.115	1.809e-05	3.850e-04
MYO9B	251.86	-0.447	0.065	7.715e-13	4.694e-11
ARHGEF18	54.20	-0.448	0.138	1.761e-04	0.0028
SLC25A29	23.84	-0.451	0.230	0.0056	0.0492
ATP6V0A1	55.15	-0.453	0.131	8.273e-05	0.0015
EPDR1	23.54	-0.454	0.203	0.0031	0.0308
HIF1A	154.85	-0.454	0.086	1.819e-08	7.283e-07
RAB27A	105.27	-0.455	0.095	2.581e-07	8.174e-06
ARID4A	46.29	-0.456	0.146	2.572e-04	0.0039
KLHL2	67.59	-0.456	0.124	3.603e-05	7.172e-04
DUS2	24.13	-0.458	0.204	0.0029	0.0290
MT-CYB	2688.57	-0.459	0.054	2.217e-18	2.231e-16
CYFIP2	22.50	-0.460	0.210	0.0034	0.0334
MT-ND2	2863.08	-0.461	0.054	3.979e-18	3.860e-16
GAPT	41.81	-0.461	0.150	2.859e-04	0.0043
PITPNC1	87.74	-0.461	0.102	1.036e-06	2.983e-05
RELL1	102.70	-0.461	0.103	1.176e-06	3.367e-05
S100A12	800.59	-0.462	0.090	3.359e-08	1.256e-06
PLCL1	19.09	-0.463	0.235	0.0053	0.0474
EXTL3	27.97	-0.465	0.195	0.0021	0.0224
MT-CO2	6446.12	-0.466	0.050	1.629e-21	2.140e-19
KDM4B	86.16	-0.466	0.111	4.035e-06	1.020e-04
CKLF	183.52	-0.469	0.082	1.399e-09	6.439e-08
CPEB4	41.85	-0.471	0.151	2.457e-04	0.0037
SLC2A6	36.04	-0.473	0.171	7.139e-04	0.0092
ABHD5	61.07	-0.473	0.137	7.764e-05	0.0014
CHST11	239.44	-0.478	0.064	7.970e-15	6.130e-13
ABCA7	42.27	-0.481	0.155	2.737e-04	0.0041

Gene	baseMean	log2FC	lfcSE	p-value	padj
SLC8B1	22.97	-0.482	0.213	0.0026	0.0266
KDM5B	20.46	-0.484	0.248	0.0049	0.0447
NBEAL2	121.81	-0.485	0.092	2.069e-08	8.130e-07
CARHSP1	32.78	-0.486	0.177	7.819e-04	0.0099
ANO10	33.94	-0.488	0.177	7.191e-04	0.0093
ZNF438	55.18	-0.489	0.138	5.438e-05	0.0010
RNF149	372.31	-0.490	0.065	8.564e-15	6.494e-13
LRCH4	62.95	-0.490	0.122	7.838e-06	1.839e-04
EPHB6	17.08	-0.490	0.244	0.0046	0.0420
IL2RG	17.14	-0.491	0.263	0.0056	0.0492
NEDD9	92.89	-0.493	0.109	8.564e-07	2.506e-05
LINC01619	103.83	-0.496	0.106	4.446e-07	1.345e-05
TMEM71	43.39	-0.496	0.147	9.837e-05	0.0017
FGD3	109.22	-0.497	0.094	1.919e-08	7.653e-07
CSF3R	561.91	-0.500	0.045	2.935e-29	5.544e-27
SEMA3C	19.96	-0.500	0.245	0.0040	0.0379
SNHG9	18.03	-0.501	0.249	0.0042	0.0392
IRS2	110.19	-0.501	0.119	3.140e-06	8.128e-05
MCTP2	27.15	-0.503	0.232	0.0029	0.0288
ASAP1	192.02	-0.505	0.071	1.399e-13	9.185e-12
TMCO3	26.26	-0.508	0.196	0.0011	0.0128
SLC24A4	79.89	-0.508	0.110	4.795e-07	1.442e-05
SLCO3A1	284.35	-0.509	0.068	9.470e-15	7.131e-13
ATG9A	16.05	-0.510	0.265	0.0049	0.0445
MAP2K7	32.43	-0.511	0.179	4.734e-04	0.0065
INPP5A	32.59	-0.511	0.174	3.891e-04	0.0055
BRF1	20.59	-0.512	0.228	0.0025	0.0258
MAP4K4	139.80	-0.513	0.088	8.039e-10	3.813e-08
CRTC3	37.58	-0.514	0.169	2.747e-04	0.0041
NAMPT	380.40	-0.515	0.069	1.056e-14	7.786e-13
CDK14	29.60	-0.516	0.227	0.0022	0.0229
CCNG2	17.57	-0.519	0.243	0.0031	0.0308
ACSL1	276.54	-0.520	0.072	9.754e-14	6.524e-12
PRAM1	216.31	-0.523	0.069	1.509e-15	1.222e-13
MARC1	34.82	-0.527	0.177	3.242e-04	0.0047
C19orf25	37.53	-0.533	0.186	4.252e-04	0.0060
MIR646HG	25.05	-0.533	0.233	0.0020	0.0213
ZBTB16	240.66	-0.536	0.093	1.020e-09	4.754e-08
DOT1L	28.52	-0.537	0.212	0.0011	0.0131
IL4R	36.94	-0.540	0.171	1.709e-04	0.0028
DPEP2	91.74	-0.541	0.106	4.393e-08	1.615e-06
PGS1	53.13	-0.542	0.134	6.479e-06	1.550e-04
SLC6A6	58.97	-0.543	0.129	2.926e-06	7.629e-05
SLC19A1	27.15	-0.551	0.211	8.869e-04	0.0110
IPCEF1	64.20	-0.554	0.138	6.885e-06	1.640e-04
C4orf48	219.39	-0.554	0.080	4.136e-13	2.620e-11
PCNX1	95.35	-0.554	0.106	2.107e-08	8.195e-07
ADAMTSL4-AS1	147.90	-0.556	0.092	2.382e-10	1.187e-08
LIPE-AS1	16.79	-0.565	0.259	0.0025	0.0257
NDEL1	33.74	-0.566	0.174	1.224e-04	0.0020
ABAT	26.35	-0.573	0.208	5.107e-04	0.0070
LINC00877	38.16	-0.574	0.166	5.892e-05	0.0011
MAPK8IP3	83.87	-0.574	0.117	1.183e-07	4.079e-06
PFKFB4	50.30	-0.575	0.147	9.975e-06	2.252e-04
SORL1	189.30	-0.578	0.075	1.007e-15	8.273e-14
METTL22	28.56	-0.579	0.193	2.542e-04	0.0039
FCAR	38.98	-0.581	0.171	6.688e-05	0.0012
VNN2	52.83	-0.584	0.137	2.216e-06	6.042e-05
MAST3	50.83	-0.585	0.147	6.806e-06	1.625e-04
FAM157C	50.43	-0.587	0.152	1.183e-05	2.617e-04
MINK1	54.08	-0.588	0.139	2.417e-06	6.426e-05
DIP2A	34.07	-0.591	0.173	6.690e-05	0.0012
DGKG	53.09	-0.594	0.140	2.267e-06	6.132e-05
CDA	130.43	-0.594	0.095	4.308e-11	2.285e-09
LINC00482	11.18	-0.597	0.327	0.0046	0.0423
RAE1	25.11	-0.598	0.208	3.703e-04	0.0053
MTMR3	107.34	-0.599	0.105	1.319e-09	6.094e-08
WLS	18.62	-0.600	0.242	0.0011	0.0127
KLHL36	18.04	-0.600	0.253	0.0014	0.0162
TESC	56.66	-0.603	0.130	3.882e-07	1.181e-05
CRISPLD2	59.35	-0.604	0.146	3.211e-06	8.292e-05
SFXN5	33.93	-0.605	0.176	5.684e-05	0.0010
S100A8	9142.31	-0.606	0.066	4.529e-21	5.670e-19
C22orf34	23.75	-0.607	0.279	0.0020	0.0214
DNMT3A	13.02	-0.609	0.357	0.0050	0.0450
NACC2	94.46	-0.613	0.110	2.274e-09	1.029e-07
BCL2A1	47.55	-0.614	0.152	5.233e-06	1.281e-04

Gene	baseMean	log2FC	lfcSE	p-value	padj
ABTB1	97.59	-0.618	0.102	1.428e-10	7.218e-09
NLRP12	79.41	-0.618	0.126	8.927e-08	3.152e-06
LPAR1	31.83	-0.620	0.182	5.933e-05	0.0011
ANPEP	111.57	-0.620	0.147	2.249e-06	6.114e-05
MED26	17.48	-0.627	0.246	8.460e-04	0.0106
FAM107B	77.72	-0.630	0.118	1.144e-08	4.700e-07
TOM1L2	28.94	-0.631	0.192	9.262e-05	0.0016
VSTM1	35.31	-0.632	0.177	3.143e-05	6.338e-04
ZHX2	14.82	-0.632	0.289	0.0020	0.0218
MTMR4	12.12	-0.633	0.316	0.0030	0.0302
AQP9	53.23	-0.634	0.137	3.442e-07	1.071e-05
GNG2	66.19	-0.635	0.122	2.108e-08	8.195e-07
CYP27A1	71.86	-0.641	0.129	5.964e-08	2.148e-06
CCDC88B	118.43	-0.644	0.109	3.595e-10	1.751e-08
ITPRIP	40.41	-0.648	0.153	2.273e-06	6.133e-05
P2RX1	55.69	-0.650	0.140	3.640e-07	1.120e-05
ZRANB1	15.60	-0.650	0.277	0.0014	0.0160
RCN3	26.02	-0.651	0.199	9.399e-05	0.0016
AC020916.1	87.52	-0.651	0.123	1.268e-08	5.193e-07
USP20	11.57	-0.659	0.386	0.0044	0.0408
MCEMP1	37.01	-0.660	0.176	1.644e-05	3.540e-04
SLC11A1	433.80	-0.665	0.055	2.590e-34	6.487e-32
SULT1B1	15.65	-0.669	0.278	0.0011	0.0128
SLC44A2	40.13	-0.670	0.171	8.664e-06	2.011e-04
SAP25	33.58	-0.674	0.181	1.647e-05	3.540e-04
RFX2	83.39	-0.676	0.147	3.798e-07	1.162e-05
AP002381.2	15.89	-0.682	0.267	7.696e-04	0.0098
RBP7	144.31	-0.683	0.114	1.576e-10	7.931e-09
AL132709.8	13.67	-0.686	0.300	0.0015	0.0167
TMEM154	94.30	-0.692	0.103	1.906e-12	1.122e-10
NR6A1	24.01	-0.693	0.235	2.290e-04	0.0035
TDRD9	9.16	-0.693	0.398	0.0045	0.0412
SVIL	80.01	-0.695	0.119	4.476e-10	2.161e-08
RGS2	193.16	-0.702	0.090	4.028e-16	3.415e-14
AC215522.2	12.02	-0.709	0.331	0.0019	0.0210
CCDC125	12.31	-0.711	0.309	0.0013	0.0155
LTB4R	45.54	-0.724	0.147	7.217e-08	2.573e-06
SLC25A37	103.79	-0.724	0.097	9.768e-15	7.304e-13
RRP12	82.93	-0.727	0.120	1.169e-10	5.937e-09
CAMK2G	30.22	-0.746	0.188	5.543e-06	1.350e-04
LIMK2	37.95	-0.752	0.165	4.257e-07	1.291e-05
LPAR2	23.87	-0.760	0.206	1.826e-05	3.878e-04
IGF1R	27.73	-0.760	0.212	2.403e-05	4.975e-04
CSGALNACT1	25.46	-0.763	0.260	2.137e-04	0.0034
STEAP4	19.06	-0.763	0.264	2.382e-04	0.0037
DLEU7	8.26	-0.766	0.383	0.0025	0.0256
PADI2	22.89	-0.766	0.231	6.667e-05	0.0012
LINC00937	117.37	-0.766	0.114	1.510e-12	8.935e-11
AC087741.1	15.54	-0.770	0.281	4.214e-04	0.0059
DTNB	13.05	-0.771	0.294	5.312e-04	0.0072
AC015912.3	19.95	-0.771	0.246	1.240e-04	0.0021
TECPR2	27.50	-0.772	0.207	1.374e-05	3.013e-04
ULK1	50.68	-0.780	0.142	3.186e-09	1.418e-07
DGAT2	10.95	-0.783	0.319	8.655e-04	0.0107
BASP1	50.93	-0.794	0.168	1.568e-07	5.260e-06
DACH1	15.30	-0.795	0.274	2.426e-04	0.0037
ADAM19	26.04	-0.821	0.207	4.801e-06	1.183e-04
LIN7A	29.07	-0.823	0.224	1.425e-05	3.111e-04
MBOAT2	8.64	-0.824	0.397	0.0019	0.0212
EHD1	28.18	-0.840	0.196	1.483e-06	4.158e-05
PTPN7	8.79	-0.842	0.425	0.0022	0.0233
LUCAT1	36.95	-0.865	0.176	7.142e-08	2.555e-06
AL356356.1	8.77	-0.876	0.427	0.0019	0.0207
TP53I11	8.35	-0.877	0.385	0.0013	0.0151
FSTL3	9.21	-0.881	0.355	7.232e-04	0.0093
IL1R2	43.37	-0.886	0.277	8.881e-05	0.0015
EEPD1	27.02	-0.900	0.209	1.061e-06	3.047e-05
ATP2A3	27.47	-0.902	0.197	3.450e-07	1.071e-05
LINC01001	38.14	-0.909	0.182	4.506e-08	1.650e-06
SLC2A3	114.86	-0.920	0.112	1.494e-17	1.399e-15
AL163541.1	30.61	-0.922	0.223	2.334e-06	6.251e-05
CXCR2	5.43	-0.945	0.538	0.0031	0.0309
PDLIM7	35.59	-0.945	0.175	4.230e-09	1.867e-07
ADGRE3	10.05	-0.946	0.352	3.889e-04	0.0055
NINJ2	14.82	-0.950	0.283	4.604e-05	8.853e-04

Gene	baseMean	log2FC	lfcSE	p-value	padj
TNFRSF10C	14.07	-0.963	0.299	7.489e-05	0.0013
ADAMTS5	5.15	-0.974	0.588	0.0032	0.0318
HAL	22.77	-0.998	0.237	1.584e-06	4.431e-05
RFLNB	37.88	-1.001	0.206	5.606e-08	2.026e-06
PLEKHG3	13.67	-1.013	0.298	4.086e-05	8.029e-04
TPST1	21.93	-1.026	0.297	3.040e-05	6.165e-04
ALOX5AP	43.96	-1.070	0.197	2.902e-09	1.297e-07
AL139042.1	7.30	-1.071	0.651	0.0054	0.0478
PADI4	66.94	-1.118	0.183	4.396e-11	2.320e-09
MMP25	14.60	-1.154	0.335	2.876e-05	5.865e-04
CHD7	12.43	-1.176	0.395	9.775e-05	0.0017
VNN3	7.75	-1.243	0.444	2.384e-04	0.0037
TENM1	5.30	-1.305	0.529	6.390e-04	0.0085
LOXHD1	10.16	-1.348	0.372	1.632e-05	3.529e-04
AFF2	2.32	-1.406	1.030	0.0038	0.0367
PROK2	13.18	-1.447	0.333	6.640e-07	1.970e-05
CMTM2	7.61	-1.498	0.433	2.596e-05	5.314e-04
ABCG1	4.52	-1.538	0.630	6.814e-04	0.0089
ETS1	2.36	-1.547	0.869	0.0029	0.0295
CST7	2.50	-1.741	1.050	0.0025	0.0263
RILPL1	2.50	-1.742	0.854	0.0032	0.0314
S100P	21.70	-1.974	0.260	1.715e-15	1.368e-13

Supplementary Table S3. Summary of donor-level external contextualization and scoring sensitivities.

The table summarizes comparisons between complete iNPH cluster 5 and externally selected IFN-high classical monocytes from the Perez atlas (Perez et al., 2022). Signature scoring excluded the 15 IFN-selector genes from the positive set and used AUCell on raw counts or mean-positive-minus-mean-negative expression on log-normalized counts. The selected-cell score analyses excluded donors with fewer than ten qualifying cells; the retained donor numbers were score-specific. Burden analyses used the full monocyte populations and the shared donor-balanced healthy-control-anchored selector threshold. Cluster concordance is the fraction of cluster-5 cells meeting the marker rule, not proof of equivalent populations or independent validation. Two-sided Wilcoxon rank-sum P values are nominal and unadjusted. Diagnosis is confounded with dataset, cell selection differs between iNPH and the external groups, and the same healthy-control dataset calibrates and evaluates the shared threshold. The results are descriptive; the healthy-control comparison was method-dependent and does not establish an iNPH disease effect.

Engine	Analysis	Group	n (donors)	Value	P (vs iNPH)	Note
AUCell (primary)	Population-level score	iNPH cluster 5 (complete, unfiltered)	9	0.0307	-	Reference group
AUCell (primary)	Population-level score	SLE, interferon-high	143	0.0702	6.42×10^{-6}	
AUCell (primary)	Population-level score	Healthy control, interferon-high	99	0.0252	0.213	
AUCell (primary)	IFN-high burden (%)	iNPH	9	13.5	-	Reference group
AUCell (primary)	IFN-high burden (%)	SLE	162	91.5	1.29×10^{-5}	
AUCell (primary)	IFN-high burden (%)	Healthy control	99	25.0	0.0128	Scoring-method-dependent — see interpretation note
AUCell (primary)	Cluster 5 / IFN-high overlap	Cluster 5 (n = 940 cells)	-	95.5% of cluster 5 is IFN-high	-	Shared donor-balanced threshold = 0.0973
Mean-difference (sensitivity)	Population-level score	iNPH cluster 5 (complete, unfiltered)	9	0.0725	-	Reference group
Mean-difference (sensitivity)	Population-level score	SLE, interferon-high	139	0.1886	1.48×10^{-6}	
Mean-difference (sensitivity)	Population-level score	Healthy control, interferon-high	99	0.0599	0.356	
Mean-difference (sensitivity)	IFN-high burden (%)	iNPH	9	30.1	-	Reference group
Mean-difference (sensitivity)	IFN-high burden (%)	SLE	162	91.8	0.00098	
Mean-difference (sensitivity)	IFN-high burden (%)	Healthy control	99	25.0	0.491	Not significant — scoring-method-dependent
Mean-difference (sensitivity)	Cluster 5 / IFN-high overlap	Cluster 5 (n = 940 cells)	-	99.5% of cluster 5 is IFN-high	-	Shared donor-balanced threshold = 0.2517

iNPH showed consistently lower interferon-response activity and burden than SLE across both scoring methods. The iNPH-versus-healthy-control comparison was scoring-method-dependent and is therefore exploratory rather than a definitive disease effect. The high fraction of cluster-5 cells meeting the marker rule supports its IFN-response-high annotation; because cluster 5 contains only a subset of the broader marker-defined population, the two definitions should not be treated as equivalent.

Supplementary Table S4. Primary THP1 top-30 compound ranking and post hoc candidate annotations.

Compounds are ordered by PRIMARY_150 NCS, with more negative values indicating stronger inverse connectivity. Baricitinib is included separately at primary rank 39. BRD identifiers preserve the identity of the scored entries; unresolved identities remain labeled by identifier. Regulatory annotations refer to the specified active ingredient, formulation, jurisdiction, and source, and do not imply approval or suitability for iNPH. Candidate reporting categories were assigned post hoc. The primary ranking is distinct from the integrated robustness ranking in Supplementary Table S5. WTCS, weighted connectivity score; FDR, saved Benjamini–Hochberg-adjusted WTCS P value. Exact-zero scores are retained in the complete numerical screening data even though this top-ranked subset contains nonzero values.

Rank	Compound	BRD ID	NCS	WTCS	FDR	Regulatory status	Pharmacologic class	Candidate tier	Verification source / note
1	Rosuvastatin	BRD-K82941592	−1.845	−0.487	6.14×10^{-4}	Approved (FDA)	HMG-CoA reductase inhibitor (statin)	Highlighted post hoc candidate	Marketed statin
2	GDC-0068	BRD-K43002773	−1.710	−0.451	6.14×10^{-4}	Investigational	AKT inhibitor	Not FDA-approved	= ipatasertib; Phase II–III oncology
3	Methyl-fasudil	BRD-K12683773	−1.509	−0.398	6.14×10^{-4}	Investigational	ROCK inhibitor (fasudil analog)	Not FDA-approved	Parent fasudil approved only in Japan/China.
4	BRD-K92317137	BRD-K92317137	−1.496	−0.395	6.14×10^{-4}	Excluded	Not identified	Excluded (unidentified)	No name in any local database or public search
5	Oglemilast	BRD-K92303087	−1.495	−0.394	6.14×10^{-4}	Investigational	Phosphodiesterase-4 (PDE4) inhibitor	Not FDA-approved	Phase II only (asthma/COPD)
6	Ethinyl estradiol	BRD-A02367930	−1.477	−0.390	6.14×10^{-4}	Approved (FDA; ingredient)	Estrogen (synthetic)	Other approved predicted reversal candidate	FDA label 021676s020
7	Indoximod	BRD-K93255255	−1.469	−0.387	6.14×10^{-4}	Investigational	IDO-pathway inhibitor	Not FDA-approved	Phase 2 (NCT04049669)
8	Metoclopramide	BRD-K75641298	−1.442	−0.380	6.14×10^{-4}	Approved (FDA)	Dopamine D2 receptor antagonist	Other approved predicted reversal candidate	DailyMed
9	AM-24	BRD-K59013864	−1.408	−0.371	6.14×10^{-4}	Investigational	Lipoxygenase inhibitor	Not FDA-approved	= Bobel-24; Broad metadata lists Phase 2
10	ZM-39923	BRD-K40624912	−1.399	−0.369	6.14×10^{-4}	Investigational	JAK1/3 inhibitor	Not FDA-approved	Research-use-only; never entered clinical development
11	T-5224	BRD-K84925803	−1.384	−0.365	6.14×10^{-4}	Investigational / discontinued	AP-1 (c-Fos) inhibitor	Not FDA-approved	Phase 2; did not lead to approval
12	GW-5074	BRD-K07859598	−1.381	−0.364	6.14×10^{-4}	Preclinical	c-Raf inhibitor	Not FDA-approved	Research use only
13	Bemegride	BRD-K14993104	−1.380	−0.364	6.14×10^{-4}	Historical / unclear	CNS stimulant (analeptic)	Not FDA-approved	No current FDA/EMA product identified
14	Roflumilast	BRD-K03194791	−1.375	−0.363	6.14×10^{-4}	Approved (FDA)	Phosphodiesterase-4 (PDE4) inhibitor	Highlighted post hoc candidate	Marketed PDE4 inhibitor
15	XMD-1150	BRD-K01436366	−1.374	−0.362	6.14×10^{-4}	Preclinical	Kinase inhibitor (specific target not verified)	Not FDA-approved	Research compound
16	AV-608	BRD-K96615647	−1.367	−0.361	6.14×10^{-4}	Investigational / discontinued	NK1 receptor antagonist	Not FDA-approved	Phase 2; discontinued
17	Troglitazone	BRD-A13084692	−1.350	−0.356	6.14×10^{-4}	Withdrawn	PPAR-γ agonist (thiazolidinedione)	Not FDA-approved	Approved 1997; withdrawn 2000 (hepatotoxicity)
18	Altanserin	BRD-K00610438	−1.318	−0.348	6.14×10^{-4}	Investigational	5-HT2A receptor antagonist	Not FDA-approved	PET ligand; Phase 2
19	Irbesartan	BRD-K60038276	−1.312	−0.346	6.14×10^{-4}	Approved (FDA)	Angiotensin II receptor blocker	Other approved predicted reversal candidate	DailyMed
20	Guanadrel	BRD-A14262390	−1.310	−0.346	6.14×10^{-4}	Discontinued (US)	Adrenergic neuron blocker	Not FDA-approved	Formerly Hylorel; discontinued
21	Mebendazole	BRD-K77987382	−1.299	−0.343	6.14×10^{-4}	Approved (FDA)	Benzimidazole anthelmintic (microtubule inhibitor)	Other approved predicted reversal candidate	FDA-approved since 1974; currently marketed as Emverm
22	Ischemin	BRD-K82181189	−1.297	−0.342	6.14×10^{-4}	Preclinical	CBP/p300 bromodomain inhibitor	Not FDA-approved	Research use only
23	CHEMBL-374350	BRD-K59962020	−1.294	−0.341	6.14×10^{-4}	Investigational	Not verified	Not FDA-approved	Obscure screening compound; no trade name
24	Ataluren	BRD-K94830329	−1.289	−0.340	6.15×10^{-4}	Not authorized (EMA non-renewal)	Nonsense-mutation readthrough agent	Not FDA-approved	Translarna EU authorization not renewed (Mar 2025)
25	BRD-K59019422	BRD-K59019422	−1.283	−0.339	6.18×10^{-4}	Excluded	Not identified	Excluded (unidentified)	No name in any local database or public search
26	Clomifene	BRD-K29950728	−1.282	−0.338	6.18×10^{-4}	Approved (FDA)	Selective estrogen receptor modulator	Other approved predicted reversal candidate	FDA-labeled clomiphene citrate
27	GSK-2334470	BRD-K70642949	−1.281	−0.338	6.18×10^{-4}	Preclinical	PDPK1 (3-phosphoinositide-dependent protein kinase-1) inhibitor	Not FDA-approved	Research use only

Rank	Compound	BRD ID	NCS	WTCS	FDR	Regulatory status	Pharmacologic class	Candidate tier	Verification source / note
28	Histamine	BRD-K01674964	−1.274	−0.336	6.36×10^{-4}	Approved (EMA formulation only)	Histamine receptor agonist	Borderline — not in final candidates	Ceplene (+IL-2, AML); LINCS entity may not match formulation
29	Linifanib	BRD-K99749624	−1.273	−0.336	6.36×10^{-4}	Investigational / unsuccessful	Multi-target RTK inhibitor (VEGFR/PDGFR)	Not FDA-approved	Phase 3 HCC; failed primary endpoint
30	C-646	BRD-K68548958	−1.271	−0.335	6.40×10^{-4}	Investigational	p300/CBP HAT inhibitor	Not FDA-approved	Research use only
39	Baricitinib	BRD-K53581288	−1.231	−0.325	8.17×10^{-4}	Approved (FDA/EMA)	Janus kinase 1/2 (JAK1/2) inhibitor	Post hoc secondary candidate	= Olumiant; RA 2018, alopecia areata 2022

Supplementary Table S5. Top-20 integrated candidate-robustness summary.

Compounds are ordered by median average-tie rank percentile across the 11 core THP1 analyses, with the documented tie-breaking rules. Columns report primary-query NCS and WTCS FDR, numbers of negative and exact-zero core scores, numbers meeting the operational NCS/FDR rule across the core and conditional donor-omission queries, and nominal compound-specific matched-null probabilities. Lower-tail empirical probabilities are not FDR values and were not adjusted for selecting candidates or testing multiple compounds. Curated names and identity qualifications are to BRD identifiers; the ranking reflects computational consistency rather than clinical suitability.

Supplementary Table S5. Integrated ranking and robustness metrics.

Integrated rank	BRD ID	Compound	PRIMARY_150 THP1 NCS	PRIMARY_150 WTCS FDR	Negative core analyses	Zero core analyses	Operational core hits	Operational LOO hits	Nominal matched-null lower-tail P
1	BRD-K82941592	rosuvastatin	-1.845	6.14×10^{-4}	11/11	0	11/11	9/9	0.0099
2	BRD-K43002773	GDC-0068	-1.710	6.14×10^{-4}	11/11	0	11/11	9/9	0.0099
3	BRD-K94830329	ataluren	-1.289	6.15×10^{-4}	11/11	0	11/11	9/9	0.0099
4	BRD-A02367930	ethinyl-estradiol	-1.477	6.14×10^{-4}	11/11	0	11/11	9/9	0.0099
5	BRD-K59013864	AM-24	-1.408	6.14×10^{-4}	11/11	0	11/11	9/9	0.0099
6	BRD-K12683773	methyl-fasudil	-1.509	6.14×10^{-4}	10/11	1	10/11	9/9	0.0099
7	BRD-K99749624	linifanib	-1.273	6.36×10^{-4}	10/11	1	10/11	9/9	0.0099
8	BRD-K60038276	irbesartan	-1.312	6.14×10^{-4}	11/11	0	10/11	9/9	0.0099
9	BRD-A51820102	econazole	-1.193	1.38×10^{-3}	10/11	1	10/11	9/9	0.0198
10	BRD-K40624912	ZM-39923	-1.399	6.14×10^{-4}	11/11	0	11/11	9/9	0.0099
11	BRD-U86686840	(R)-AZD-7762 stereoisomer	-1.233	8.17×10^{-4}	10/11	1	10/11	9/9	0.0297
12	BRD-K59962020	CHEMBL-374350	-1.294	6.14×10^{-4}	10/11	1	10/11	9/9	0.0099
13	BRD-K93255255	indoximod	-1.469	6.14×10^{-4}	11/11	0	11/11	9/9	0.0099
14	BRD-K92303087	oglemilast	-1.495	6.14×10^{-4}	10/11	1	10/11	9/9	0.0099
15	BRD-K03194791	roflumilast	-1.375	6.14×10^{-4}	10/11	1	10/11	9/9	0.0099
16	BRD-K70642949	GSK-2334470	-1.281	6.18×10^{-4}	10/11	1	10/11	9/9	0.0297
17	BRD-K07859598	GW-5074	-1.381	6.14×10^{-4}	6/11	5	6/11	7/9	0.0099
18	BRD-K84925803	T-5224	-1.384	6.14×10^{-4}	11/11	0	10/11	9/9	0.0099
19	BRD-A13084692	troglitazone	-1.350	6.14×10^{-4}	10/11	1	9/11	9/9	0.0099
20	BRD-A72711497	lasalocid	-1.225	8.94×10^{-4}	9/11	0	7/11	8/9	0.0594

Supplementary Table S6. Non-overlapping Hallmark IFN-α-response sensitivity in THP1.

Compounds were queried against LINCS2 using the 49 represented genes among the 54 genes from the MSigDB Hallmark interferon-α-response set M5911 (Liberzon et al., 2015) that did not overlap the iNPH signature. Of the 97 Hallmark genes, 42 overlapped the positive iNPH signature and one overlapped the negative signature, giving 43 unique exclusions. This is a non-overlapping-gene interferon-pathway sensitivity analysis, not a positive control or independent validation. NCS, Normalized Connectivity Score; FDR, Benjamini–Hochberg-adjusted P value. Compounds without a listed NCS/FDR were either not profiled in THP1 despite being present in the LINCS2 screening library or were not identified in the available reference data; these categories are distinguished in the THP1 profiling-status column without assigning an unverified reason.

Compound	Pharmacologic category	THP1 profiling status	NCS	FDR	Connectivity direction	Interpretation / note
Rosuvastatin	Highlighted candidate (PRIMARY_150 query)	Profiled	−2.2427	1.043×10^{-4}	Negative connectivity	Significant reversal; most negative NCS in this report set
Roflumilast	Highlighted candidate (PRIMARY_150 query)	Profiled	−1.2500	1.043×10^{-4}	Negative connectivity	Significant reversal
Baricitinib	Secondary candidate (PRIMARY_150 query, post hoc)	Profiled	−0.7772	0.08445	Negative connectivity	Negative connectivity without statistical significance in this Hallmark query; the primary-query THP1 result was significant.
Tofacitinib	JAK inhibitor (approved)	Profiled	−1.0656	2.255×10^{-4}	Negative connectivity	Significant reversal; a second LINCS2 entry for this compound was not profiled in THP1
Ruxolitinib	JAK inhibitor (approved)	Profiled	−1.2454	1.043×10^{-4}	Negative connectivity	Significant reversal
Upadacitinib	JAK inhibitor (approved)	Absent from the available reference data	-	-	-	Not identified in the available LINCS2 reference data
Filgotinib	JAK inhibitor (approved)	Not profiled in THP1	-	-	-	Not identified in the available THP1 reference data
Methotrexate	Conventional DMARD	Not profiled in THP1	-	-	-	Not identified in the available THP1 reference data
Sulfasalazine	Conventional DMARD	Not profiled in THP1	-	-	-	Not identified in the available THP1 reference data
Leflunomide	Conventional DMARD	Profiled	+1.2074	1.043×10^{-4}	Positive connectivity	Significant positive connectivity
Hydroxychloroquine	Conventional DMARD	Profiled	+0.9130	2.171×10^{-3}	Positive connectivity	Significant positive connectivity
